# A Comprehensive Review of Group-III Nitride Light-Emitting Diodes: From Millimeter to Micro-Nanometer Scales

**DOI:** 10.3390/mi15101188

**Published:** 2024-09-25

**Authors:** Xinye Fan, Jiawang Shi, Yiren Chen, Guoqing Miao, Hong Jiang, Hang Song

**Affiliations:** 1 Key Laboratory of Luminescence Science and Technology, Chinese Academy of Sciences & State Key Laboratory of Luminescence and Applications, Changchun Institute of Optics, Fine Mechanics and Physics, Chinese Academy of Sciences, Changchun 130033, China; 2 Department of Optical Science and Engineering, School of Information Science and Technology, Fudan University, Shanghai 200438, China; 3 School of Optoelectronics, University of Chinese Academy of Sciences, Beijing 100049, China

**Keywords:** light-emitting diodes, group-III nitrides, micro-LEDs, nano-LEDs, luminescence

## Abstract

This review describes the development history of group-III nitride light-emitting diodes (LEDs) for over 30 years, which has achieved brilliant achievements and changed people′s lifestyles. The development process of group-III nitride LEDs is the sum of challenges and solutions constantly encountered with shrinking size. Therefore, this paper uses these challenges and solutions as clues for review. It begins with reviewing the development of group-III nitride materials and substrates. On this basis, some key technological breakthroughs in the development of group-III nitride LEDs are reviewed, mainly including substrate pretreatment and p-type doping in material growth, the proposal of new device structures such as nano-LED and quantum dot (QD) LED, and the improvement in luminous efficiency, from the initial challenge of high-efficiency blue luminescence to current challenge of high-efficiency ultraviolet (UV) and red luminescence. Then, the development of micro-LEDs based on group-III nitride LEDs is reviewed in detail. As a new type of display device, micro-LED has drawn a great deal of attention and has become a research hotspot in the current international display area. Finally, based on micro-LEDs, the development trend of nano-LEDs is proposed, which is greener and energy-saving and is expected to become a new star in the future display field.

## 1. Introduction

**Figure 1:**
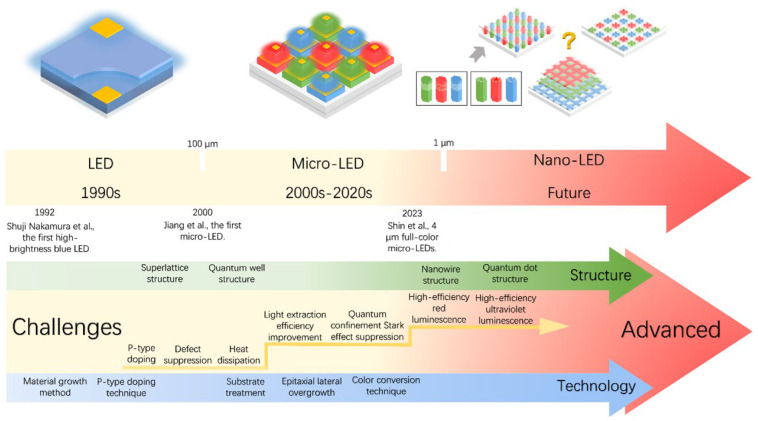
From millimeters to micrometers to nanometers scale in group-III nitride LEDs [3,4,13].

From millimeters to micrometers to nanometers, the quest for smaller-scale manipulation in semiconductors seems to remain unchanged. In the field of semiconductors, it is well-known that arsenide, phosphide, nitride, and their alloys can be widely used in LEDs. However, in the past, once upon a time, arsenides and phosphides, which were used to make red LEDs and green LEDs, respectively, had long been produced, but materials that made high-brightness blue LEDs were lacking. Today, direct wide-bandgap group-III nitride materials are considered ideal optoelectronic materials due to their high thermal conductivity, considerable carrier mobility, small dielectric constant, and stable physical and chemical properties, which can be used to fabricate blue LEDs for high-efficiency lighting, but no one had any illusions about it until the 90s [1]. Since Professor Shuji Nakamura successfully developed the world’s first GaN-based high-brightness LED in the early 1990s with a single chip area of 0.6 × 0.5 mm^2^, humanity has officially entered the door of solid-state lighting [2,3]. Subsequently, with the continuous improvement in the manufacturing process level of photoelectric devices, the first micro-LED array with an area of 0.5 × 0.5 mm^2^ was born in 2000. It was reported by Jiang et al. in 2001, including 10 × 10 pixels, and each chip size is 12 µm [4]. Since then, research on micro-LED has attracted many people and developed rapidly. New ideas continue to emerge, such as the three-terminal LEDs reported by Memon et al., the mass-transferable LED wafers reported by Chang et al., and the exciton luminescence-based nanowire LEDs reported by Pandey et al. [5,6,7]. As Figure 1 illustrates, more and more LEDs are manufactured for the same area; the shrinking of the single-pixel size from several hundred microns in the initial LED to several microns in micro-LEDs has brought new advancements, including functions and applications. At the same time, manufacturing technology has also ushered in new challenges. The advancement of micro-LED in functions such as brightness, power consumption, lifetime, response time, and resolution has led to the development of entirely new application fields such as wearable smart devices, augmented and virtual reality displays, visible light communication (VLC), and medical applications, which creates enormous economic benefits [8,9,10,11]. Spontaneously, the enormous economic benefits brought about by the advances in the field of micro-LED have attracted numerous research institutions to invest in it. Relevant data show that the market value of micro-LEDs is expected to reach 17 billion US dollars by 2025, and the number of related publications from 2016 to 2019 has nearly tripled [12].

In addition, from another angle, how about the challenges? At present, group-III nitride LEDs are focusing on solving the problems of high-efficiency p-type doping, defect suppression, quantum confinement Stark effect (QCSE) suppression, light extraction efficiency (LEE) improvement, heat dissipation, etc., brought by the challenge of high-efficiency luminescence in the ultraviolet (UV) and red bands. Responding to challenges to make it more advanced, researchers continue to innovate in the structure and technology of LED. The structures of superlattices, quantum wells, nanowires, QDs, etc., are developed. Technological breakthroughs continue to be represented by improvements in material growth methods, novel p-type doping, substrate treatment, epitaxial lateral overgrowth (ELOG), color conversion, and other technologies. As depicted in Figure 1, the continuous solution of problems and the emergence of new scientific research ideas have prompted people to put forward higher requirements for the performance of micro-LEDs, which has also promoted the development of LEDs in a more advanced direction, resulting in new challenges that will continue to grow in the future. Faced with these challenges and inspired by the tremendous benefits of LEDs due to their size reduction to the micrometer scale, the question is raised as to whether continuing to reduce device size to the nanometer scale would solve the micro-LEDs problem and bring more gains. For this reason, the research on nano-LEDs has been increasing gradually in recent years.

Credibly, you and I have one common curiosity about how to solve the above problems, so let us join forces and explore, beginning with the research progress, including the material, structure, and technology of LED, then comprehend the advancement of micro-LEDs in size effect, quantum efficiency improvement, spectral characteristics, and the application and development of micro-LEDs in various fields. Finally, this paper discusses the existing problems of nano-LEDs for the future and tries to find a new way to solve the issues so as to make a reasonable expectation of its prospects. Above all, I hope you find this review helpful and have a better idea of the future in the field in which I work.

## 2. Group-III Nitride Materials and Substrates

### 2.1. Properties of Group-III Nitride Materials

#### 2.1.1. Crystal Structure of Group-III Nitride Materials

**Figure 2:**
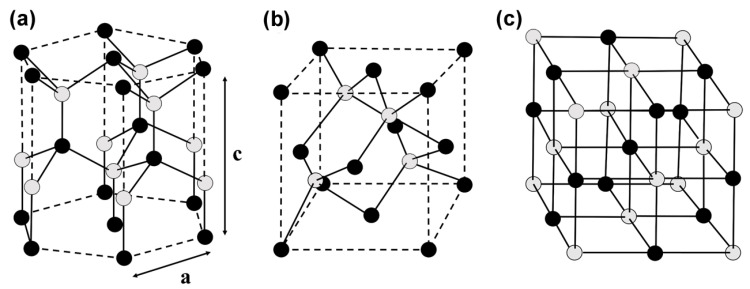
The diagram shows the crystal structure of group-III nitrides. (**a**) Wurtzite structure. (**b**) Zinc-blende structure. (**c**) NaCl structure.

The successful synthesis of the first GaN single crystal in 1969 opened the prelude to research on the properties of group-III nitride semiconductors [14]. Group-III nitrides usually have two crystal structures: wurtzite and zinc-blende. The wurtzite structure consists of alternating hexagonal layers of nitrogen and group-III metal atoms. The term “cubic zinc-blende” refers to a space complex structure that consists of two types of atomic crystal structures: The face-centered cubic structure composed of two kinds of atoms is translated by a quarter along each autogenous diagonal. In most cases, group-III nitrides are hexagonal wurtzite structures, and at low temperatures, heterostructure epitaxy forms zinc-blende structures. They also form sodium chloride (NaCl) structures under extremely high pressure [15]. The three crystal structures are shown in Figure 2a, Figure 2b, and Figure 2c, respectively. The zinc-blende structure makes it clear that there is one atom on each face center, while the NaCl structure is a regular square structure. The wurtzite structure is represented by three a-axes with the bottom surface at 120° from each other and a c-axis perpendicular to all three a-axes. The lattice constant (a, c) of the group-III nitride alloy material ($A_{x}B_{1-x}N$) of the wurtzite structure is according to the following formulas:

(1) $$a^{A_{x}B_{1-x}N}=x\cdot a^{AN}+\left(1-x\right)\cdot a^{BN}$$

(2) $$c^{A_{x}B_{1-x}N}=x\cdot c^{AN}+\left(1-x\right)\cdot c^{BN}$$

#### 2.1.2. The Polarity of Group-III Nitride Materials

Previously, the polarization effect of group-III nitrides has been explained by others, but it needs to be more convenient and easier to understand. This section will start with the most basic knowledge of atomic arrangement and covalent bonds and introduce the polarization effect of group-III nitrides in detail.

Polarity is a unique property of the group-III nitride material system that results from the asymmetric central crystal structure of wurtzite and zinc-blende structures. Even more so in wurtzite, the covalent bond along the c-axis is longer than the other three covalent bonds, and the crystal structure is more asymmetric. This polarity results in piezoelectric and spontaneous polarization in group-III nitriding materials. Take GaN material as an example. GaN material is arranged alternately by the Ga atomic and N atomic layers. Arranged along the directions [0001] and [000$\bar{1}$] parallel to the c-axis, the corresponding N atom is at the bottom layer, and the Ga atom is at the bottom, respectively (Refer to Figure 3 in Ref. [16] for easier understanding). After the epitaxial growth of the material, two polar planes are formed along the two directions. These are called Ga plane polarity and N plane polarity, respectively.

**Figure 3:**
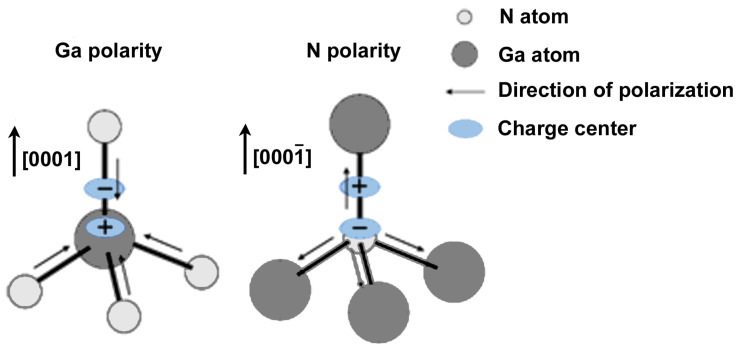
Polarization of GaN.

Under the action of external forces, the crystal deformation separates the positive and negative charge centers, which results in generating an internal electric field and polarizing charges on the surface, known as the piezoelectric polarization effect. In particular, the wurtzite structure of group-III nitride materials has lower non-central symmetry, longer bond length along the c-axis, and no coincidence of positive and negative charge centers in the absence of external force, resulting in a polarization effect called spontaneous polarization. At the microscopic level, taking GaN as an example, due to the difference in electronegativity between Ga atoms and N atoms (N atoms are larger than Ga atoms), when a Ga atom and an N atom form a covalent bond, the shared electron pair is more inclined toward the N atom. The direction of polarization on the covalent bond is that the N atom points to the Ga atom. At this time, the centers of positive and negative charges are separated. Figure 3 depicts the center distribution of positive and negative charges. Therefore, judging from the above, the spontaneous polarization is along the direction [000$\bar{1}$]. When the spontaneous polarization effect occurs, because the polarization direction is from negative charge to positive charge, there is a negative bound charge on the Ga polar surface and a positive bound charge on the N polar surface. When the epitaxial film is subjected to tensile stress, taking the Ga plane as an example, the angle between the covalent bond formed by three N atoms and Ga atoms and the direction of the c-axis increases, resulting in the weakening of their co-polarization along the guide [0001], which is equivalent to the generation of piezoelectric polarization in the direction [000$\bar{1}$], which is the same as spontaneous polarization. Similarly, taking the Ga surface as an example, when the epitaxial film is subjected to compressive stress, the angle between the covalent bond formed by three N atoms and Ga atoms and the direction of the c-axis decreases, resulting in the enhancement of their co-polarization along the direction [0001], and the direction of piezoelectric polarization is opposite to the direction of spontaneous polarization. The polarization effect of group-III nitrides is a double-edged sword. Reasonable utilization of the polarization effect of group-III nitride materials can improve the transport behavior of carriers (refer to [17]) and hole concentration (this will be explained in Section 3.4.3). However, the QCSE generated by the polarization effect will affect the performance of the LED (this will be explained in detail in Section 3.2.2).

#### 2.1.3. Group-III Nitride Alloy Materials and Luminescence

Currently, the main direct band gap III-nitride materials used for luminescence are AlN, GaN, and InN and their ternary and quaternary alloys. The optical band gaps of AlN and GaN at room temperature are around 6.2 eV and 3.4 eV, respectively [18,19]. However, the optical band gap of InN at room temperature has been controversial for a long time, and the currently accepted value is about 0.7 eV. The study of the InN optical band gap can be referred to in a review article by Butcher et al. [20]. Their ternary alloy and quaternary alloy material band gaps meet the following formula:

(3) $$E_{g}^{{Al}_{x}{In}_{y}{Ga}_{1-x-y}N}\left(x,y\right)=x\cdot E_{g}^{AlN}+y\cdot E_{g}^{InN}+\left(1-x-y\right)\cdot E_{g}^{GaN}-b_{AlGaN}\cdot x\cdot \left(1-x\right)-b_{InGaN}\cdot y\cdot (1-y)$$

where $E_{g}^{AlN}=6.2\;eV$, $E_{g}^{InN}=0.7\;eV$, $E_{g}^{GaN}=3.4\;eV$, $b_{AlGaN}$, and $b_{InGaN}$ are bowing parameters, which can be deduced by fitting the bandgap of the alloy determined by PL or by absorption measurements. The x (0 ≤ x ≤ 1) and y (0 ≤ y ≤ 1) factors are on behalf of components of aluminum and indium in the alloy, respectively.

The peak wavelength of the LEDs’ luminescence spectrum with their alloy material as the active region can be estimated by the following formula:

(4) $$\lambda_{0}=\frac{1240}{E_{g}^{{Al}_{x}{In}_{y}{Ga}_{1-x-y}N}\left(x,y\right)}\left(nm\right)$$

This means that by adjusting the content of each component of the group-III nitride alloy, their LEDs can emit light in a range from ultraviolet to infrared (around 200 nm to 1771 nm). However, the actual luminous peak may deviate slightly. For example, the quantum-confined Stark effect on LED luminescence due to polarization will be introduced in the following chapters.

### 2.2. Substrates

Currently, sapphire (Al_2_O_3_), silicon (Si), and silicon carbide (SiC) are often used as substrates for LEDs, which have both advantages and disadvantages. It is necessary to select the appropriate substrate as required.

#### 2.2.1. Sapphire

**Figure 4:**
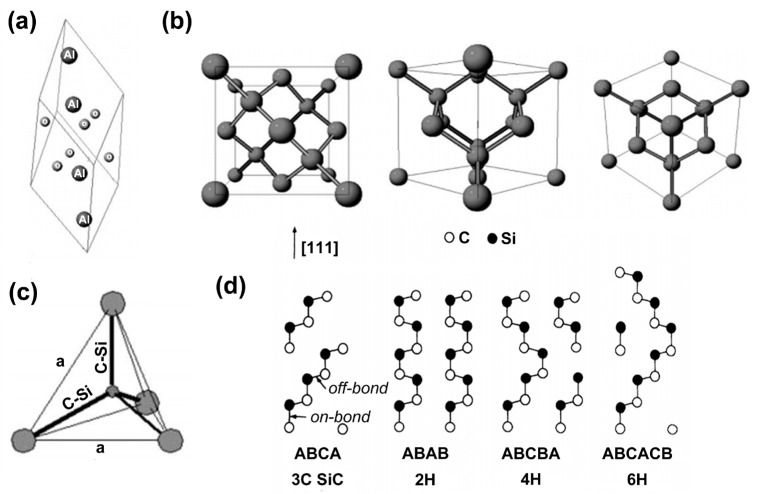
(**a**) The unit cell of sapphire. (**b**) Perspective views of Si along various directions: [001], [011], and [111]. (**c**) Single-molecule SiC model. Reproduced with permission [22]. Copyright 2002, Elsevier Publishing. (**d**) Structure diagrams of 3C-SiC, 2H-SiC, 4H-SiC, and 6H-SiC in the hexagonal (10$\bar{1}$0) plane. Reproduced with permission [23]. Copyright 1994, APS Publishing.

Sapphire is a hexagonally wurtzite-structured material (as shown in Figure 4a) that has high thermal stability and strength. The main component is α-Al_2_O_3_, which is used as an epitaxial growth substrate for group-III nitride materials. Because of its large band gap, it can be used as a substrate for ultraviolet radiation C (UVC) and longer-wavelength LEDs without causing light absorption [21].

On the opposite side, large lattices and thermal mismatches (1000 °C) exist between the c-plane sapphire and epitaxial layer materials (AlN, GaN). This means that with the continuous change in temperature during the material growth process, the stress on the epitaxial layer will cause a large number of defects and, more seriously, even lead to the appearance of cracks, which will eventually lead to a significant decrease in LED performance. Second, due to the poor thermal conductivity of the sapphire substrate, the useless Joule heat converted from a part of the electric energy will be dissipated very slowly when the LED is working, and the continuous accumulation of heat will cause the power consumption to rise and the lifespan to be shortened. Third, since the refractive index of air and sapphire is smaller than that of GaN, it is well known that when light enters from an optically denser medium into an optically thinner medium, a part of the light greater than the critical angle will be totally reflected and confined in the device. Even more frightening is that this part of the light will be converted into other forms of energy, such as heat, which further negatively affects the performance of the LED. In aiming to solve these problems with sapphire substrates, replacing or stripping the substrate is an optional method.

#### 2.2.2. Silicon

Silicon (Si) is the second most abundant element in the Earth’s crust, and the preparation technology of high-purity single-crystal silicon is very mature, which is the cornerstone of the human information industry. The silicon crystal has a diamond structure, as shown in Figure 4b. In LED manufacturing, Si substrates have a higher thermal conductivity than sapphire substrates (about four times that of sapphire), a lower cost, and the preparation of large-scale and high-quality Si substrates has been industrialized, making them suitable for mass production of high-power LEDs. However, similar to the sapphire substrate, the silicon substrate also suffers from a high lattice mismatch and thermal mismatch (1000 °C) with epitaxial materials. In detail, the lattice mismatch of the silicon substrate is slightly higher, and the thermal mismatch is less than that of the c-plane sapphire substrate. Besides this, due to the nature of the material itself, Si and GaN will melt at high temperatures to form a Si-Ga alloy so that the GaN epitaxial layer is severely etched. Finally, it cannot be ignored that the band gap of silicon of 1.12 eV results in the theoretical absorption of light with wavelengths below 1107 nm, which is one of the main reasons for the obvious decrease in the external quantum efficiency (EQE) of LEDs.

#### 2.2.3. Silicon Carbide

As a representative material of the third-generation semiconductor, silicon carbide (SiC) is often used as a substrate in the LED field. A single SiC molecule has a tetrahedral structure as in Figure 4c. According to the different lattice arrangement orders, the common types in the [0001] direction are the ABCB-arranged 4H-SiC substrate and the ABC ACB-arranged 6H-SiC substrate, with slightly different properties. The crystal structures of 4H-SiC and 6H-SiC are shown in Figure 4d [23]. In comparison to sapphire and silicon substrates, SiC has excellent thermal conductivity and smaller lattice and thermal mismatches with GaN. SiC’s thermal conductivity is roughly sixteen times that of sapphire and three times that of silicon. Both 4H-SiC and 6H-SiC are smaller than c-plane sapphire and silicon substrates in terms of lattice mismatch and thermal mismatch (1000 °C) with epitaxial materials. It can be said that the application of SiC can significantly reduce the heat dissipation, lattice mismatch, and thermal mismatch problems of other substrates. In addition, the refractive index of SiC is similar to that of GaN and its alloy materials, so the light emitted from the active area rarely has total reflection, and SiC hardly absorbs light with wavelengths above 415 nm, which avoids the additional loss of the emitted light, significantly improving the EQE and light extraction efficiency (this will be explained in detail in subsequent sections) of the device. However, although pure SiC is colorless and transparent, there are many impurities in the material in industrial manufacturing, which reduce the transmittance of SiC and cause the brightness of LEDs to decrease. Moreover, the current manufacturing of high-quality SiC large-size wafers has always been difficult, resulting in a low yield rate, which greatly increases the cost of SiC substrates and hinders industrialization. And it cannot be ignored that SiC has a strong absorption of UV light, so it is not suitable for the substrate of UV LEDs. For the curious, more detailed information about the physical properties of sapphire, Si, and SiC substrates can be found in the review article by Liu et al. [22].

#### 2.2.4. Other Substrates

With LEDs’ increasingly demanding performance requirements and the need for new application scenarios, other material substrates represented by metals, oxides, etc., have appeared and developed continuously. Compared with the traditional substrates (sapphire, Si, and SiC) above, metal substrates have great advantages in thermal conductivity. They are specially used to control the junction temperature and prolong the life of LEDs. Moreover, the high reflectivity of the metal substrate can improve the LEE of the LEDs, and the fact that the electrodes can be fabricated directly on the metal substrate can improve the production efficiency of the LEDs. However, the high thermal mismatch between the metal substrate and group-III nitride epitaxial material and the interface reaction under a high-temperature growth environment restrict the high-quality growth of epitaxial material and seriously affect the performance of the device. Choosing metal substrates with a slight thermal mismatch (such as Mo or W) and realizing low-temperature material epitaxially via pulsed laser deposition technology is a good solution to address these issues. In addition to metal substrates, some oxide substrates (such as ZnO or LiGaO_2_) have also been extensively studied. These oxide substrates have advantages in lattice mismatch, thermal mismatch, and avoidance of QCSE. However, the high-temperature interfacial reaction caused by metal–organic chemical vapor deposition (MOCVD) and molecular beam epitaxy (MBE) and the manufacturing difficulties of large substrates are the main factors restricting the high performance of LEDs on oxide substrates. More detailed information about metal and oxide substrates, such as material epitaxy and related parameters, can be found in the review articles by Li et al. and Wang et al. [24,25].

With the advancement in LED industrialization and the development of wearable devices, in order to pursue larger substrate manufacturing to reduce manufacturing costs and meet the requirements of flexible transferability of LED chips, amorphous substrates, represented by quartz, have emerged. It is obvious that the growth of crystalline GaN on an amorphous substrate has a negative effect on the epitaxial layer quality, so researchers have developed a variety of material growth methods. For example, GaN crystals can be grown using graphene as a buffer layer, ZnO nanowalls as an intermediate layer, or graphene as a buffer layer combined with pulsed sputtering deposition, plasma-assisted molecular beam epitaxy, and other material growth technologies to finally achieve crystal GaN growth [26,27,28]. Furthermore, GaN epitaxy by growing pyramidal arrays on amorphous substrates is also reported [29].

### 2.3. Material Growth Methods

The growth of high-quality materials is the basis for ensuring the high performance of LEDs. Over the years, researchers have mastered a variety of growth methods for group-III nitride materials. At present, the growth methods of group-III nitride materials mainly include MOCVD, MBE, and hydride vapor phase epitaxy (HVPE). These three growth methods have their own advantages and disadvantages. In general, HVPE technology can achieve a high growth rate while retaining the benefits of simple growth technology, but the quality of the film grown by HVPE technology is generally not outstanding. However, if the material quality requirements are high, MBE and MOCVD growth methods can be considered. MBE technology can grow ultrahigh-quality thin films and complex structures using plasma- and laser-assisted methods, but its growth cost is expansive, and its growth rate is the slowest of the three. It can be said that HVPE and MBE have their own distinct advantages and disadvantages. Compared with HVPE and MBE growth technologies, MOCVD can guarantee film quality while taking a high growth rate and low cost into account. Its advantages of a widely controllable growth rate, accurate control of multiple alloy material components, simple equipment maintenance, and low cost are commonly used in industrial production and scientific research. Of course, under some special requirements, HVPE and MBE technologies are still mainstream, and it has also been developed to grow high-quality thin films by using them together with MOCVD.

Initially, GaN thin films were grown on sapphire substrates by the HVPE method in 1969 [30]. With the improvement of people’s requirements for film quality, MBE technology was first applied by Yoshida et al. in 1983 to grow an AlN buffer layer to adjust the stress of sapphire and GaN [31]. The growth of GaN thin films by the MOCVD method was achieved by Amano et al. in 1986 by inserting an AlN insertion layer [32]. However, due to insufficient material growth methods, blue, high-brightness LEDs were not successfully developed. Fortunately, this changed in 1990 when Nakamura Shuji et al. developed a new key technology for GaN growth [33]. They changed single-flow MOCVD to dual-flow MOCVD, which has two gas pipes containing the main gas flow parallel to the substrate carrying the reactive gas and a sub-current of inert gas perpendicular to the substrate. A continuous GaN thin film is thus obtained, creating conditions for the success of subsequent high-brightness blue LEDs. After decades of development, the growth of III-group nitride materials has become more mature. Finally, the relevant research on using different technologies to grow nitride materials on various substrates in recent years is summarized as a reference in Table 1. And the relevant material growth parameters can be found by references in the table.

### 2.4. The Source of Challenges

It can be said that the challenges of LED come from the nature of the materials themselves. No matter what materials are chosen for the epitaxial layer and substrate, between the substrate and the epitaxial layer and between different materials of the epitaxial layer, common challenges are faced. It suffers from negative piezoelectric effects, defects caused by lattice mismatch and thermal stress mismatch, and decreased luminous efficiency due to weak electron confinement in the light-emitting region. And the poor electrical conductivity of the substrate causes uneven current distribution, which results in the area junction temperature being high. The opaque electrode produces a blocking effect on the outgoing light, and the light absorption of the material itself reduces the LEE. As mentioned above, the sapphire substrates’ thermal conductivity is poor, which inhibits the heat dissipation of the chip and shortens its life, and the Fresnel reflex and even the total reflection phenomenon reduce the LEE. For silicon substrates, the Si-Ga alloy etch-back phenomenYeon and visible light absorption affect its performance. For SiC substrates, the reason for the high cost is attributed to the manufacture of the substrate itself. Some of these issues have been effectively addressed, and unresolved issues remain challenging. What deserves more consideration is that there are some special problems with LEDs that still need to be improved and cannot be ignored. The first is the difficulty of p-type doping of group-III nitride materials, especially AlGaN alloy materials with high aluminum components. The second is the difficulty of light extraction of AlGaN materials. Finally, the quality of group-III nitride materials with high aluminum and indium components is poor.

The above problems are the main obstacles that affect the luminous efficiency of LEDs during the manufacturing process. At present, through some material structure and technical innovations, as well as size reduction, which will be analyzed in detail in Section 3 and Section 4, some of the above problems have been well solved, especially with high-efficiency blue and green LEDs. However, the situation is not ideal when it comes to high-efficiency UV and red LEDs.

## 3. Ever-Improving Structures and Technologies

### 3.1. Substrate Treatment

#### 3.1.1. Beveled Substrate

Proper pretreatment of substrates can improve the quality of material growth. Correspondingly, a beveled substrate operation that reduces dislocation density without introducing additional growth steps is a good option. Hao et al. systematically analyzed the effect of chamfering the sapphire substrate on its subsequent material growth and found that the quality of the GaN buffer layer improved with the chamfer angle from 0° to 0.5°, and when the sapphire substrate is chamfered at the same angle, the residual film internal compressive stress of GaN on the c-biased m-plane is smaller than that on the c-biased a-plane [61]. And it has also been reported that slant cutting of the substrate sidewall is beneficial for improving the light extraction of LEDs to show better performance [62,63].

#### 3.1.2. Patterned Sapphire Substrate

Using etching technology to etch periodic three-dimensional convex or concave nano- or micro-scale structures on the surface of the substrate is another effective and popular means to improve the growth quality of materials, which is called the “patterned sapphire substrate” (PSS). The principle that a patterned substrate improves material quality and luminous efficiency by suppressing the non-radiative recombination centers, which are formed by dislocation defects, stems from its ability to promote the lateral growth of the epitaxial layer, which can twist the dislocations and form closed dislocation loops to suppress dislocation density (the details will be explained in Section 3.2.3). Relevant studies have shown that different three-dimensional shapes on the patterned substrate surface and the same shape have different depths, cone inclination angles, and distances between the shapes, which will affect the luminous efficiency of LEDs to varying degrees. In more detail, some typical studies are listed. The study of the hole-like PPS with a depth ranging from 0.5 µm to 1.5 µm found that the EQE improved the most when the depth was set at 1.5 µm [64]. In the study of the tapered pattern, it was found that the dislocation density of the buffer layer decreases with the decrease in the tilt angle [65]. When the distance and size between the patterns were investigated, it was found that the light output power increased with the decrease in the distance of the tapered pattern, and the luminous efficiency increased with the reduction in the pattern size within an appropriate range, where too small a size would reduce crystal quality [66,67,68]. Relevant data and rules can be found in Table 2. Presently, PSS and beveled substrates have been used as convenient means to improve the epitaxial quality of materials. There is a belief that a new idea for combining these two methods is already on the way. In addition to suppressing defects, the principle of reducing the pattern pitch and proper size to improve the luminous efficiency is to improve the external light emission (light extraction efficiency, LEE). In more detail, when the light generated by the LED strikes the sapphire substrate, the light can be scattered in various directions due to the existence of the three-dimensional pattern. Then, when the scattered light reaches the interface between the LED material and the external environment, the part of the scattered light whose incident angle is less than the critical angle will not be affected by the total reflection phenomenon and will successfully exit the LED to improve the luminous efficiency. Nowadays, in order to reduce the experimental cost and improve efficiency, the PPS-related simulation based on the TracePro optical analysis software is widely used to qualitatively and quantitatively obtain the optimal shape and its geometric parameters. For more research progress and prospects on applying patterned sapphire substrate to group-III-nitride LEDs, please refer to the review article by Zhou et al. [69].

Of course, similar to the principle of PPS improving the performance of LEDs, studies on patterned silicon substrates have also been reported [70]. However, related studies have shown that although patterned silicon substrates are of great help in improving the quality of the material, they have residual stress compared to the structure of the LED material grown on a sapphire substrate [71].

#### 3.1.3. AlN Template

**Figure 5:**
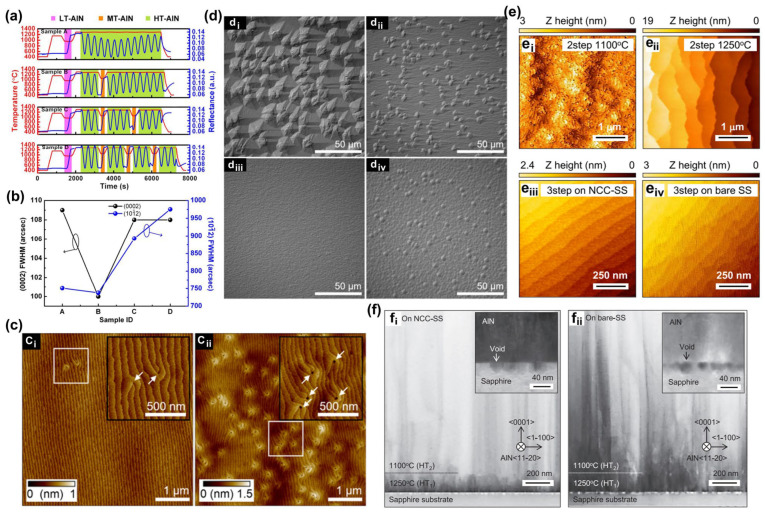
(**a**) The in situ monitoring curves vs. time sequence for AlN templates. The left ordinate is the process temperature, and the right ordinate is the short-wavelength reflectance. (**b**) Comparison of FWHM between the two-step growth method AlN template (sample A) and the three-step growth method AlN with different interlayer thicknesses (samples B, C, and D). Reproduced with permission [72]. Copyright 2018, RSC Publishing. (**c**) Atomic force microscope (AFM) diagram of AlN template face-to-face annealed sputter-deposited on the beveled (0.2°) sapphire substrate (**c_i_**) and the AlN template grown by MOCVD (**c_ii_**). (**d**) Nomarski microscopy images of the AlGaN layer surface were grown on the face-to-face annealed sputter-deposited AlN template with the sapphire substrate at different beveled angles (0.2° (**d_i_**), 0.6° (**d_ii_**), and 1.0° (**d_iii_**)) and on the MOCVD-AlN template (**d_iv_**). Reproduced with permission [56]. Copyright 2020, AIP Publishing. (**e**) AFM images of AlN templates grown by the two-step growing method at different temperatures ((**e_i_**) for 1100 °C and (**e_ii_**) for 1250 °C) and the AlN templates grown by the three-step growing method on PPS (**e_iii_**) and conventional sapphire substrates (**e_iv_**). (**f**) Structure diagram of AlN templates grown on PPS (**f_i_**) and conventional substrate (**f_ii_**) observed by cross-sectional transmission electron microscopy. Reproduced with permission [73]. Copyright 2017, AIP Publishing.

The AlN template is pre-grown on the substrate mainly to solve the difficulty of direct growth of AlGaN material, which also plays a role in stress relief. To obtain a high-quality AlN template and give full play to its role in stress relief, related research continues to progress. Regarding the AlN template, we have to mention the two-step growth method pioneered by Amano et al. in 1986 [32]. The so-called “two-step growth method” is first to adjust the growth parameters to grow the three-dimensional island AlN nucleation center conducive to stress release and then change the growth parameters to grow the two-dimensional layered AlN to bend and annihilate the dislocation inside so as to achieve the purpose of high-quality materials finally. Based on the two-step growth method, the three-step growth method was developed. The so-called “three-step growth method” inserts an intermediate epitaxial layer between the low-temperature three-dimensional AlN nucleation layer and the high-temperature two-dimensional growth layer to release the stress further. Table 3 lists some comparative parameters of AlN templates obtained by two- and three-step growth methods. The results show that the quality of the AlN template is further improved by this method compared with the two-step method [72]. Figure 5a,b show the variation in their growth temperature and the comparison of their growth quality, respectively. The growth of AlN templates based on the three-step growth method on the patterned sapphire substrate is reported. The full width at half maximum (FWHM) of the (0002) plane and the (10$\bar{1}$2) plane are 50 arcsec and 250 arcsec, respectively [73] (Figure 5c,d show the relevant experimental results). In recent years, the face-to-face annealing sputtering method for growing AlN templates has been widely used and achieved good results. Uesugi et al. grew high-quality AlN templates with the FWHMs of 13 arcsec and 123 arcsec for the (0002) plane and the (10$\bar{1}$2) plane, respectively, by face-to-face annealing sputtered AlN on a beveled substrate [56]. Figure 5e,f show the relevant experimental results.

### 3.2. Planar Layer Structure

By combining one or several materials of AlN, AlGaN, GaN, InGaN, and InN into the corresponding layer material structure and controlling the composition ratio of Al or In in their own alloy material (AlGaN, InGaN), defect suppression, stress regulation, and luminescence enhancement can be achieved. With the development of group-III nitride material epitaxy methods and for the purpose of improving performance, the material structure of LEDs has evolved from a single material with a single PN junction as the active area to multiple materials with multiple quantum wells (MQWs) as the active area. In general, in order to release stress and restrain defects, the intermediate buffer layer design of LEDs from the substrate to the active region follows the principle of component gradient, which can adopt several constant component layers and component gradient layers. The lattice parameter gradient in the composition gradient layer has a positive effect on stress relief, and its induced piezoelectric polarization in group-III nitride materials can also optimize carrier transport behavior [17]. The electron barrier layer is also applied to inhibit electron leakage, which will be mentioned in the following content. Based on the simple planar layer structures mentioned above, more complex systems, semiconductor superlattices (SLs), and MQWs structures are reported in detail here.

#### 3.2.1. Semiconductor Superlattice

**Figure 6:**
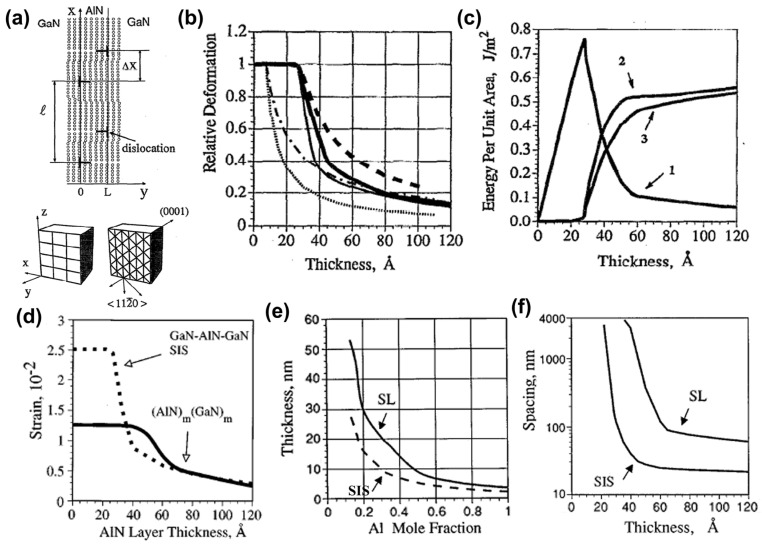
(**a**) GaN-AlN-GaN SIS structure diagram with mismatched dislocation and schematic diagram of isotropic slip (**lower left**) and hexagonal slip (**lower right**) system. (**b**) Function diagram of relative deformation along the interface and AlN thickness. (**c**) Energy per unit area as a function of AIN film thickness. 1—uniform contribution within the AIN film; 2—nonuniform contribution without the core energy; 3—tore energy. Reproduced with permission [78]. Copyright 1995, AIP Publishing. (**d**) Strain as a function of AlN layer thickness in (GaN)_m_(AlN)_m_ SLs (solid line) and GaN-AlN-GaN SIS structures (dashed line). (**e**) The critical thickness as a function of Al concentration in (GaN)_n_(Al_x_Ga_1−x_N)_n_ SLs (solid line) and in GaN-AlN-GaN SIS (dashed line). (**f**) The spacing between dislocations for (GaN)_n_(AlN)_n_ superlattice (upper curve) and GaN-AlN-GaN SIS structure (lower curve) as a function of the layer thickness. Reproduced with permission [79]. Copyright 1997, AIP Publishing.

A semiconductor superlattice is a periodic planar layer structure composed of the alternating growth of different semiconductor materials with a thickness smaller than the electron mean free path. The world’s first semiconductor superlattice was specifically discussed and successfully fabricated on gallium arsenide in 1970 [74,75]. Subsequently, the first group-III nitride-based semiconductor superlattice was reported in 1989 [76]. Based on the above, people are intensely interested in studying semiconductor SLs’ relevant characteristics and mechanisms. The mechanism of superlattice regulation of lattice mismatch is inferred from the results of Bykhovski et al.’s research on the GaN-AlN-GaN semiconductor–insulator–semiconductor (SIS) structure of thin AlN layers. That is regulation by internal strain rather than misfit dislocations [77]. Subsequently, the study of the elastic strain relaxation mechanism caused by misfit dislocations in the same SIS structure found that the initial AlN thickness of the relaxation process was 30 Å. And when the AlN thickness varies from 30 Å to 100 Å, the contribution of the elastic strain tensor decreases by order of magnitude as the spatial variation of misfit dislocations contributes to the relaxation becoming larger [78]. It can be said that the study of SIS structure has laid the foundation for the study of superlattice-related properties (Figure 6a–c show the relevant experimental results). Following the above research, in 1997, Bykhovski et al. conducted symmetric (m = n) and asymmetric (m ≠ n) (GaN)_m_(AlN)_n_, (GaN)_m_(Al_x_Ga_1−x_N)_n_, and (GaN)_m_(In_y_Ga_1−y_N)_n_ superlattice strain relaxation studies. It is found that the strain relief in the superlattice originates from the interaction between dislocations of different heterointerfaces, accompanied by the reduction in the associated deformation energy. At any m/n ratio in (GaN)_m_(AlN)_n_, the critical thickness L_c_ at which relaxation occurs ranges from 25 Å to 37 Å. For symmetric (GaN)_m_(Al_x_Ga_1−x_N)_n_, when 0.12 < x < 1, the critical thickness L_c_ is reduced by 30% to 50% and is equivalent to (GaN)_m_(In_y_Ga_1−y_N)_n_ where y = 2.33 × x [79] (Figure 6d–f show the relevant experimental results). For GaN/AlN superlattice growth, the Ga/N ratio has a key influence on the surface morphology of the superlattice, and excess GaN plays an essential role in the relaxation process. The alternating growth of hetero-material layers in the GaN/AlN superlattice induces strain fluctuations that favor the formation of V-type pits that support strain relaxation in GaN-based devices. The strain state of the superlattice after 10 to 20 cycles of growth will not be affected by the underlying material [80,81,82,83,84,85]. For the GaN/Al_x_Ga_1−x_N superlattice, with the increase in Al composition, the dislocation density increases, the mismatch relaxation is enhanced, and there is a periodic fluctuation of strain relaxation in the lattice. Unlike the fluctuations associated with plastic deformation in GaN/AlN SLs, the fluctuations in GaN/Al_x_Ga_1−x_N SLs are related to the different elastic responses of excess Ga to the two materials [86,87].

**Figure 7:**
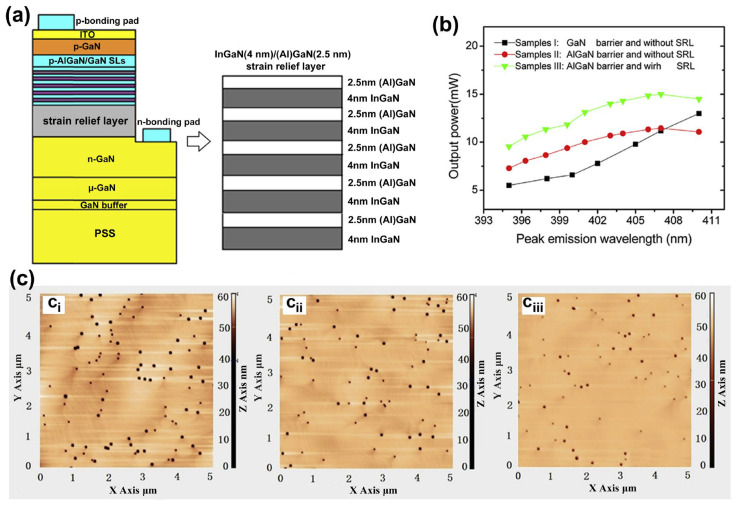
(**a**) The schematic diagram of near-UV LED samples and InGaN/AlGaN SLs strain relief layer (SRL) for samples III (AlGaN as barrier and SLs as SRL). (**b**) The relationship between output power and luminous wavelength of different LED samples. (**c**) AFMimages of In_0.04_Ga_0.96_N/GaN MQWs for sample I (**c_i_**), sample II (**c_ii_**), and sample III (**c_iii_**). Reproduced with permission [90]. Copyright 2016, Elsevier Publishing.

**Figure 8:**
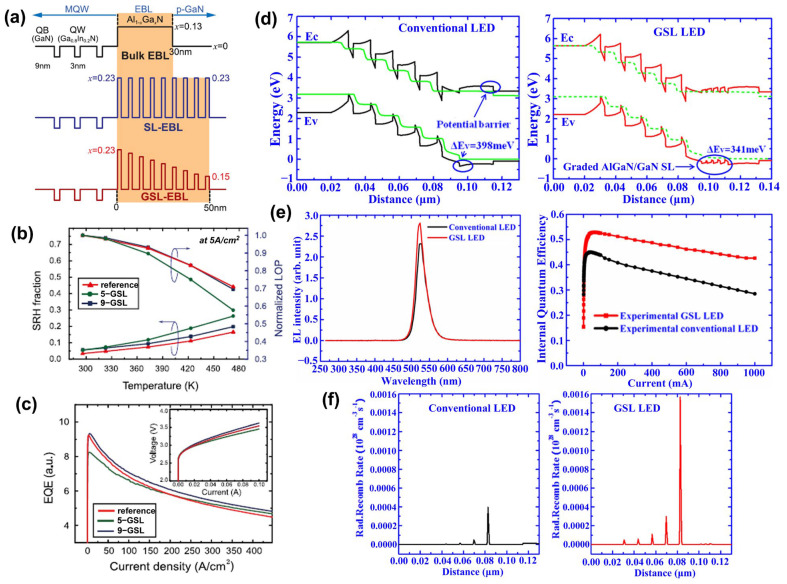
(**a**) Schematic conduction band diagrams showing the three different EBL structures: bulk EBL, SL EBL, and gradient SL (GSL) EBL. (**b**) The ShockleyReadHall (SRH) fraction of the total recombination as estimated from the AB+f(n) model fit and normalized light output power (LOP) as a function of temperature for the reference LED, the 5-period GSL EBL LED, and the 9-period GSL EBL LED. (**c**) EQE as a function of injection current for the LEDs with three different EBL structures. The inset shows the I–V characteristics. Reproduced with permission [91]. Copyright 2013, AIP Publishing. (**d**) Calculated energy band diagrams of the conventional (**left**) and GSL (**right**) LEDs at a current density of 100 A/cm^−2^. (**e**) EL spectrums of conventional and GSL LEDs at a current density of 100 A/cm^−2^ (**left**). Experimental IQE as a function of current of conventional and GSL LEDs (**right**). (**f**) Radiative recombination rates of the conventional (**left**) and GSL (**right**) LEDs at a current density of 100 A/cm^−2^. Reproduced with permission [95]. Copyright 2013, AIP Publishing.

Semiconductor SLs have a significant impact on improving the performance of optoelectronic devices. More and more superlattice structures are emerging in pursuit of higher-performance LEDs and various applications [88]. For example, as described above by the superlattice mechanism, its most important application and research is as a stress relaxation layer. When the thermal and lattice mismatches between the substrate and the LED structure material are too large, such as in AlGaN-based UV LEDs, introducing semiconductor superlattice will further alleviate the defects and improve the crystalline quality of the active region and piezoelectric effects caused by the stress [89,90] (Figure 7 shows the relevant experimental results). Elsewhere, Al-graded materials such as GaN/Al_x_Ga_1−x_N or Al_x_Ga_1−x_N/Al_y_Ga_1−y_N SLs can be used as an electron block layer (EBL) to achieve p-type induced doping and prevent electron overflow from the active region. Compared with the traditional high-Al composition AlGaN EBL, which has difficulty in p-type doping and is weakened by the polarization effect to block the electron barrier, applying the compositionally graded superlattice achieves high EQE that reduces the efficiency drop and low operating voltage [91,92] (Figure 8 shows the relevant experimental results). In addition, in order to improve the electrostatic discharge performance of LEDs and reduce the losses caused by short circuits in LED devices in industrial production, a modulation-doped semiconductor superlattice structure is used, that is, periodically alternating single or combined doping (combined doping may include intrinsic doping) of p-type or n-type, which can effectively improve crystal quality and reduce leakage paths [93,94].

#### 3.2.2. From Visible Light LEDs to Ultraviolet LEDs with MQWs Structure

In contrast with the superlattice structure, for the MQWs structure, the width of the potential barrier is thicker than that of the potential well so that the electron wave functions in the adjacent potential wells cannot overlap, and the width of its potential well is thin enough to be comparable to the de Broglie wave of electrons. The high-density electron holes with significant quantum effects are gathered in the wells, and with the increase in the number of quantum wells, the overlap of the wave functions of electrons and holes also increases, which leads to an increase in the radiative recombination luminous efficiency of LEDs. For the effect of polar and non-polar surfaces on luminescence, nitride quantum wells based on non-polar planes have advantages in enhanced radiative recombination and internal quantum efficiency (IQE) compared to quantum wells grown on polar planes [96]. At room temperature, the radiative recombination mechanism of LEDs based on non-polar surface quantum wells is dominated by exciton recombination, while the radiative recombination mechanism in LEDs based on polar surface quantum wells is dominated by localized uncorrelated electron–hole recombination, and the radiative recombination rate depends on the product of the respective densities of electrons and holes [97].

##### Visible LEDs with InGaN/GaN MQWs

Because their light-emitting wavelengths cover the visible light region (780–400 nm) entirely, LEDs with InGaN MQWs structure as the device’s light-emitting region are widely used in the lighting and display fields. By increasing the indium content of InGaN/GaN MQWs, LEDs could theoretically glow blue, green, and red in turn.

From a structural point of view, the QCSE present in InGaN/GaN MQWs is considered to be one of the reasons why the luminous efficiency droops when visible LEDs emit light in long waves (especially when greater than the 470 nm wavelength range, which is called the green-gap effect). In terms of materials, with the increase in the indium component, the dissociation temperature of InGaN material decreases, so the growth temperature of the materials has to be reduced. However, the decrease in growth temperature is not conducive to the diffusion of adsorbed atoms, which makes the material tend to grow like an island and leads to the formation of polycrystalline structures with multi-grain boundaries and eventually leads to the increase in material defects [98]. At the same time, indium segregation could cause an uneven distribution of indium components in the material [99]. In addition, N vacancies are easily formed in InGaN materials with a high indium component, which further reduces the quality of the material [100]. On the contrary, high growth temperatures will reduce indium content and also deteriorate material quality due to the destruction of the In-N bond [101]. The non-radiative recombination caused by defects decreases the luminous efficiency, and the uneven distribution of indium decreases the quality of the beam. It should be noted here that not all defects have a negative impact on LEDs; some, such as V-shaped pits, have a positive effect in some aspects, which will be detailed later.

Traditionally, the poor quality of the high-indium component InGaN material and the QCSE in its MQWs have been thought to be the reason why it is difficult for visible LEDs to emit long waves, which makes it a challenge for LEDs to achieve high-efficiency red-light emission with group-III nitrides. In recent years, with the deepening of relevant research, a completely new perspective has been proposed. That is, the localization of carriers is caused by electric fields due to indium component fluctuations and the uneven structure of MQWs. The electric field restricts electrons or holes to the narrowest band gap, and thus, the coincidence of electron and hole wave functions is reduced, the participation of carriers in radiation recombination decreases, and its non-radiative Auger recombination is increased (bandgap changes due to component fluctuations) [102,103,104,105,106].

Currently, the research hotspots and challenges based on InGaN MQWs LED are (1) high-indium component InGaN/GaN MQWs material quality improvement, (2) QCSE inhibition, (3) the study of V-shaped pits, (4) the mitigation of carrier localization in InGaN/GaN MQWs due to indium component fluctuations. It is obvious that the above four points are all aimed at alleviating the efficiency droop so as to realize the color conversion for the highly efficient red emission of the group-III nitride LEDs. The content of color conversion to achieve high-efficiency red light emission and efficiency droop will be further reported in the subsequent thematic sections (Section 4.4.1 and Section 4.2.1), and the above points will be summarized in this section.

This chapter on improving material quality is also valid for InGaN materials, such as substrate treatment and ELOG. The growth of GaN/In_x_Ga_1−x_N MQWs requires periodic control of the indium source, and the growth temperature of the potential barrier and the potential well are also different. In general, the growth temperature of barrier GaN is around 800 °C, and the temperature of growth well InGaN decreases as the indium composition increases. Further details on the MQWs growth of InGaN materials (especially InGaN materials with a high indium component) can be found in the review article by Tan et al. [107].

Research on quantum wells has been intensively studied over the past three decades. What cannot be ignored when it comes to the MQW is that, due to the spontaneous polarization and piezoelectric polarization effects of group-III nitride materials, a QCSE affects the light-emitting characteristics of LEDs in the MQWs structure. The lattice mismatch between different materials in the MQWs structure is the main reason for QCSE. When the piezoelectric polarization electric field in the MQW structure is greater than the spontaneous polarization electric field, the wells’ energy band and the barriers’ energy band are inclined in opposite directions, and the band gap between the wells is narrowed to red-shift the luminescence peak wavelength of the LED. In addition, the electron–holes in the well are separated into two sides, the probability of wave function coincidence decreases, the restriction effect of the quantum wells on the carrier is weakened, electron leakage increases, and the luminous efficiency of the LED decreases. If the current injected into the LED gradually increases, the carrier density separated by the piezoelectric polarization field on both sides would also increase. The built-in electric field generated by the piezoelectric polarization field in the opposite direction will gradually increase, and the band gap between the wells will become wider, resulting in a blue shift of the LED luminescence peak. In summary, this is the principle and phenomenon of the QCSE. In most cases, QCSE needs to be restricted, and some effective methods have been applied so far. One of the easiest ways to think of it is to grow device structures on non-polar or semi-polar surfaces [108,109]. For more detailed information, please refer to the review article by Zhao et al. [110]. The second is to change the crystal phase growth of the group-III nitride material, but this method has proven to be very difficult without degrading the crystal quality [111,112,113,114]. It is found that the QSCE of quantum well structures grown on nanometer-sized patterned sapphire substrates is further suppressed with the decrease in pattern spacing on sapphire substrates, which suggests a good approach [115]. Doping-induced generation of free carriers to generate an electric field with a shielding polarization effect is another effective means to confine QCSE. Currently, the doping methods are mainly based on doping quantum barriers (Si, Ge, Mg) and polarization-induced doping caused by the compositionally graded MQW structure [116,117,118]. Impressively, the additional growth of barrier layers on both sides of the active region for band modification has been known as Internal-Field-Guarded-Active-Region Design (IFGARD) in recent years [119]. Due to advances in growth methods, suppression of QCSE using some of the methods described above has now been extended to operate on nanowire structures [120,121].

**Figure 9:**
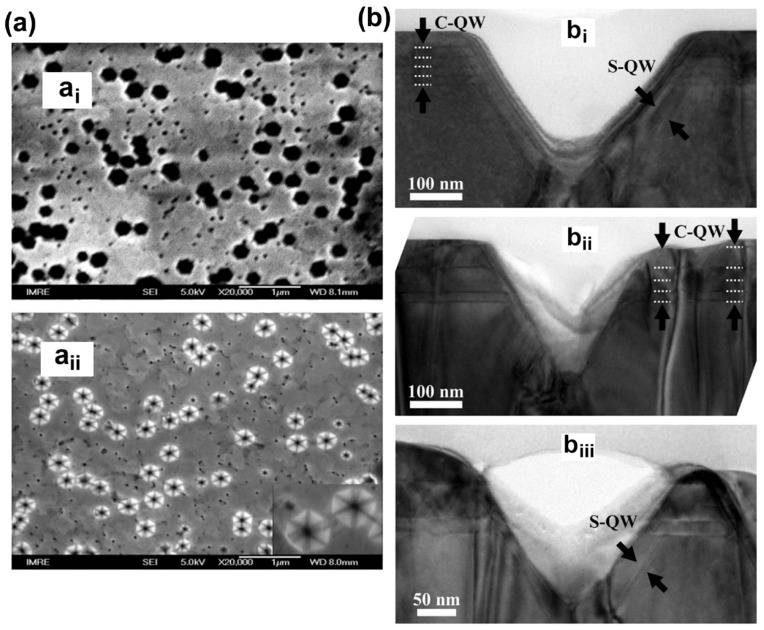
(**a**) Scanning electron microscopy images of the V-shaped pit surface for sample A (22 nm-thickness low-temperature (LT) GaN buffer (**a_i_**)) and sample B (15 nm-thickness LT GaN buffer (**a_ii_**)). (**b**) Cross-sectional transmission electron microscopy (TEM) images of the MQW regions with large V-pits, taking along the [10$\bar{1}$0] zone axis (**b_i_**) of sample A, the [11$\bar{2}$0] zone axis (**b_ii_**) of sample B, and taking near the [11$\bar{2}$0] zone axis (**b_iii_**) of sample B. Reproduced with permission [122]. Copyright 2008, AIP Publishing.

As a defect that has a far-reaching influence on the luminous performance of InGaN MQWs LEDs, V-shaped pits deserve extensive research. V-type pits are inverted pyramid-shaped defects with six sides, which widely exist in group-III nitride quantum wells [122]. The morphology of the V-shaped pit is shown in Figure 9. Its sidewall has an entire c-plane quantum well period, where indium atoms are unevenly distributed [123]. Compared with the c-plane quantum well, the thickness of the sidewall quantum well is also thinner [124]. The dislocations formed by the stress caused by the large lattice mismatch between the well and the barrier and the lower atomic mobility of the material surface at low growth temperature are the possible factors leading to the formation of V-type pits [125,126,127,128]. In the study of the luminescence characteristics of the V-type pit wall quantum well, it is found that the luminescence peak is blue-shifted compared with the c-plane quantum well, and the blue-shift increases with the increase in temperature [129,130,131]. The reason is that the stress of the quantum well near the V-type pit is smaller than that of the c-plane quantum well far away from the pit, which is beneficial to the mitigation of the QCSE and the proportion of transition carriers as the high energy level increases with the increase in temperature [132,133,134]. In addition, due to the low Mg doping concentration of the p-type group-III nitride material grown in the V-type pit, this region becomes a high-resistance and high-barrier region for dislocations, where the carriers injected into the non-radiative recombination centers formed by dislocations here are reduced, the luminous efficiency of the LEDs is improved, and the sidewall quantum well has a lower polarization charge density with low In content, making it easier for holes to be injected from the V-type pit into the active region than that from the c-plane, which further improves the quantum efficiency of LEDs [135,136,137]. V-shaped pits also make outstanding contributions toward suppressing LED reverse leakage. As the size of the V-shaped pit becomes larger, the surrounding potential barrier becomes higher, the energy cost of deep-level electrons to convert into free electrons becomes larger, and the effect of suppressing leakage increases [138]. Based on the above, the related regulation of the V-shaped pit has been carried out to enhance the optoelectronic performance of LED. Within a certain range, the number of quantum well (or superlattice) periods and the thickness of the buffer layer are positively related to the size of the V-type pit. As the number of cycles and the thickness of the buffer layer increase, which leads to an increase in the height of the sidewall quantum well barrier, holes whose area and depth are injected from the sidewall quantum well increase, resulting in a decrease in the c-plane quantum well current density and a decrease in the thermal breakdown probability. Ultimately, the electrostatic discharge yield increased, the optoelectronic performance improved, and the performance reached its best at ten cycles [139,140,141]. However, by continuing to increase the number of periods (V-type pit size), the optoelectronic performance of the device will decrease, which is explained by the worsening of the uniformity of the sidewall quantum wells and the reduction in the light-emitting area of the c-plane quantum wells. Additionally, it is found that in the device grown at a high temperature and then cooled at a low temperature, the growth is the same as the surface of the device grown at a low temperature, where there are V-type pits. However, the device surface is flat under high-temperature growth conditions [142]. By contrast, the p-GaN LED grown under variable temperature conditions has high output power and a high LEE, enhancing the LED’s electrostatic discharge characteristics [143].

The mechanism by which carrier localization reduces the efficiency of InGaN/GaN MQW LEDs has been briefly described. Its origin is currently believed to be mainly due to indium component fluctuations, which are thought to be due to metallic thermodynamic incompatibility and indium segregation, and more detailed studies are needed on this basis. Only by better understanding the origin and mechanism of carrier localization can we better study how to alleviate carrier localization in MQWs. For this reason, Sauty et al. pointed out that the localism of carriers caused by the disorder of the alloy froze the photoelectron transport, which ultimately led to a decrease in the optical emission efficiency. The indium component is positively correlated with the disorder of the alloy [144]. The present research results show that the indium content, indium component fluctuation, alloy disorder, and carrier localization of InGaN material seem to have a strong correlation. It can be inferred that with the increase in indium content, indium component fluctuation and alloy disorder in InGaN materials will be stronger. As a result, carrier localization is enhanced, which eventually leads to auger recombination enhancement, and some of them do not even participate in recombination, which shows the freezing phenomenon of carrier transport. Finally, the efficiency droop appears in LEDs. In recent years, some research has focused on alleviating carrier localization in InGaN/GaN MQWs. Carrier localization is strongly correlated with material growth conditions. Kazazis et al. confirmed this view and indicated that the selection of appropriate substrate temperature and growth conditions by the growth method of MBE is conducive to the weakening of carrier localization. Especially in the N-rich environment, when the substrate temperature is below a certain threshold, the inhibition effect of carrier localization in InGaN material is obvious [145]. Subsequent exploration of growth condition optimization has been reported. For example, when growing materials using MOCVD, special thermal annealing to redistribute carriers, GaN quantum barriers incorporating silicon, and controlling pre-epitaxial metal adsorption atoms to improve thermal stability have all been shown to reduce carrier localization to enhance luminescence [146,147,148]. In terms of the device structure, planting red InGaN quantum wells on blue InGaN quantum wells has also been shown to be effective in alleviating carrier localization [149]. However, the current research is far from enough, and more related research on the material and device structures is urgently needed.

##### Ultraviolet LEDs with AlGaN/GaN MQWs

As mentioned above, the InGaN MQWs structure is the core luminescence region of visible LEDs; for UV LEDs, its core luminescence region is AlGaN MQWs. At present, the UV-emitting quantum well is more and more inclined toward the scheme of multi-material combination with different Al components, either gradually or non-gradually. Usually, two or more materials are selected from GaN, AlGaN, AlN, and even InGaN (usually near ultraviolet) [150,151,152,153]. According to the wavelength range, the UV band is divided into UVA (400–320 nm), UVB (320–280 nm), UVC (280–200 nm), and VUV (200–10 nm). The wavelength emission coverage range based on AlGaN MQWs UV LEDs ranges from 200 nm to 400 nm by adjusting the aluminum composition, reaching most of the UV band coverage, which is widely used in medical treatment, biological detection, communication, and other fields. UV LED based on AlGaN faces many challenges. The current research focuses on the following problems: (1) epitaxial growth of high Al-component AlGaN materials with high quality and few thread dislocations, (2) P-type doping of AlGaN materials with a high Al composition is difficult, (3) AlGaN’s low electrical conductivity causes LED heat generation and dissipation problems, (4) active regions of AlGaN/GaN MQWs enhance radiation recombination to improve IQE, (5) ultraviolet AlGaN-based LED with a high Al composition has a low LEE. The solution to problem (1) can be found in the applications of the beveled substrate, graphical substrate, buffer layer, superlattice, and ELOG mentioned in this article. Applying some epitaxial growth technologies can also further improve the growth quality of AlGaN materials, such as pulsed-atomic layer epitaxy, migration-enhanced epitaxy, and the ammonia pulse-flow method. For more details on the epitaxy of high-quality materials, see the review article by Li and Mondal et al. [154,155]. For the answer to question (2), we can refer to Section 3.4. A high thermal conductivity substrate is one solution for efficient LED heat dissipation, as detailed in Section 2.2 and reference [156]. And the micro-LED that will be introduced in the Section 4.1 positively reduces the joule heat and has excellent heat dissipating capability, which can reduce the vicious cycle of unnecessary heat generation. Given this, the advantages of UV micro-LED arrays in heat dissipation have been demonstrated [157]. In addition to improving the epitaxial material quality to reduce the stress-induced piezoelectric polarization effect, this section will introduce the solution to the problem (4) for the transformation project based on the MQWs structure. As for problem (5), we will introduce it from the following content.

**Figure 10:**
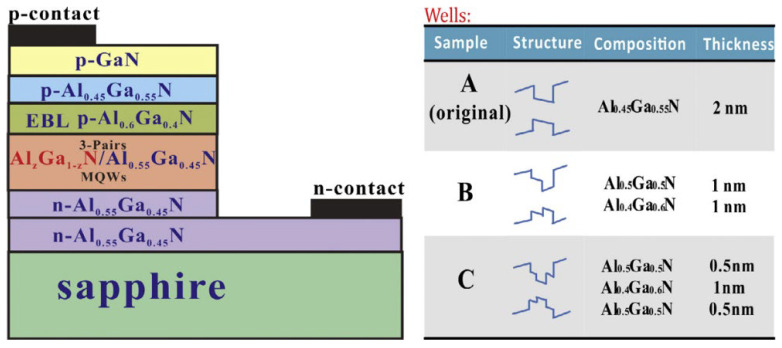
Schematics of conventional UV-LED structure on the left, the staggered quantum wells shown in the right diagram. Reproduced with permission [159]. Copyright 2014, Elsevier Publishing.

**Figure 11:**
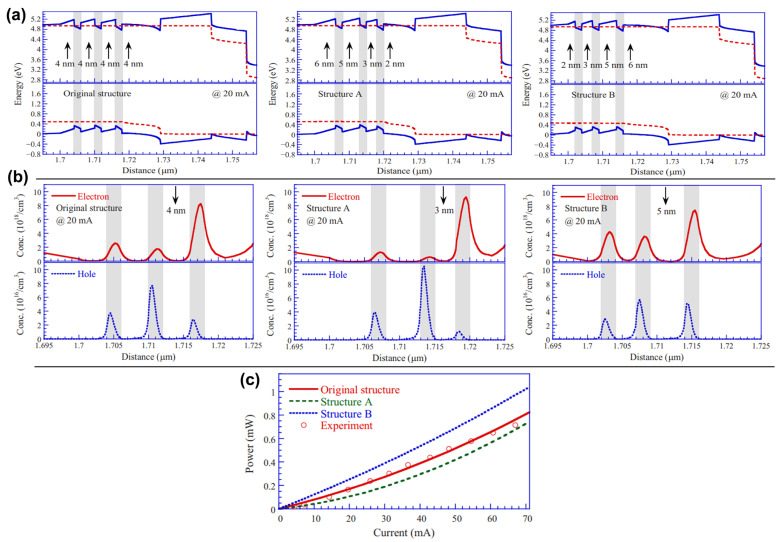
(**a**) The energy band maps of different structures. From top to bottom, they are constant quantum barrier thickness, gradually decreasing quantum barrier thickness, and gradually increasing quantum barrier thickness, which corresponds to the original structure, structure A, and structure B. (**b**) Electron and hole concentrations of the original structure (**left**), structure A (**middle**), and structure B (**right**) around the active region at 20 mA. (**c**) Output power as a function of current for the original structure, structure A, and structure B. The open circles are the data of the original structure obtained from experimental measurements. Reproduced with permission [171]. Copyright 2011, AIP Publishing.

There are many reasons for the unsatisfactory IQE of MQWs, among which the main ones are the non-radiative recombination caused by material defects and the low overlap rate of the electron–hole wave function caused by QCSE. In this section, we will introduce the improvements needed for a higher IQE regarding MQWs’ structural design and the growth of materials. In terms of improving the quality of MQWs’ materials, the relevant solutions to the problem (1) are the foundation. And when introducing visible LEDs as MQWs, we mentioned the modification of quantum wells and quantum barriers to suppress QCSE, and the method used here is similar. By simulation, the quantum well reconstruction project of inducing polarization field regulation to improve the electron–hole coincidence rate as well as alleviate efficiency droop (which will be covered in a later section) under the condition of gradual aluminum composition is considered an exciting method (As shown in Figure 10, several component structures are designed) [158,159,160]. In practice, Wang et al. grew a staggered graded quantum well structure by MOCVD and tested it, and the results were consistent with the simulation predictions [161]. Wu et al. designed a step quantum well emitting at 350 nm and found that, compared with the traditional quantum well, the QCSE inhibition is obvious, the wave function overlap is increased, and the IQE is increased by two times [150]. However, there are few reports on quantum well growth with linear and quadratic component modulation. The quantum barrier doped with Si and Mg has a positive effect on the quantum barrier reconstruction project [162]. It has been reported that Mg doping is better than Si doping in terms of lower electron leakage [163,164]. The linear, triangular, stepped, and sawtooth profiles of the quantum barrier band show the potential to solve the problem, which needs to be turned into reality [165,166,167,168]. In addition to the design of the band profile of the quantum barrier, the average Al component of the quantum barrier, the thickness of the quantum barrier, and the number of quantum wells should also be considered. For the MQWs structure emitting in the 200–280 nm band, about 10 quantum wells are the best [169]. And generally speaking, the average Al component and the thickness of the quantum barrier are positively correlated with the QCSE [170]. However, a low Al component and low barrier thickness are not conducive to carrier confinement, so a case-by-case analysis and creativity are required. For example, Tsai also reports a strategy for the gradient of the quantum barrier thickness to allow more aggressive carrier distribution formation [171] (Figure 11 shows the relevant experimental results). Is the same true for quantum wells? I believe the quantum well reconstruction project has taught us a lot, and in addition, some excellent research performed in recent years deserves attention. Guo et al. reported a method to improve wave function to overlap in AlGaN MQWs by introducing transverse polar structures, that is, integrating Al and N polar regions on the same platform [172]. Sun et al. designed tilted MQWs, which were grown on a misoriented sapphire substrate, resulting in a significant increase in IQE, with a peak value of around 275 nm up to 93% [173].

The essence of improving material quality to enhance IQE is to reduce the non-radiative recombination caused by defects. As for the effect of defects on non-radiation, unlike InGaN/GaN MQWs, threading dislocation is not the main non-recombination center in AlGaN/GaN MQWs but is mainly affected by point defects [174] (this finding may inspire new ideas for improving IQE). Sundaram et al. preferentially grew a layer of h-BN buffer layer before the growth of the AlN template. The characterization results showed that the quality of the obtained AlGaN/AlGaN MQWs material had improved. More importantly, they created a sapphire substrate stripping method without affecting the optical properties of the device [175]. Here, some pioneering ideas in material growth will be introduced. After reducing the growth rate of the AlGaN quantum barrier, Wang et al. found that the surface of the AlGaN/AlGaN MQWs became smooth and the defects were reduced, and finally, the optical output power of the LED was increased by 83% [176]. Recently, Gao et al. reported a scheme for selecting regionally grown n-GaN to enable transverse electron injection into MQWs. Compared with traditional LEDs, the threading dislocation of MQWs is reduced, and the surface morphology is better. What is more noteworthy is that the IQE is up to 95% [177].

In the long run, in terms of improving the IQE of MQWs, it is not only necessary to design a reasonable quantum well structure with breakthroughs but also to make it a reality through material growth. In addition, there may be unexpected gains from subverting the tradition, such as the introduction of new material system coordination, the introduction of new growth processes, and the device material structure of MQWs with transverse electron and hole injection. It is a wild guess that in the future, MQWs with half or even full coverage of electron and hole injection layers may be the direction to enhance IQE.

#### 3.2.3. Epitaxial Lateral Overgrowth Technology

**Figure 12:**
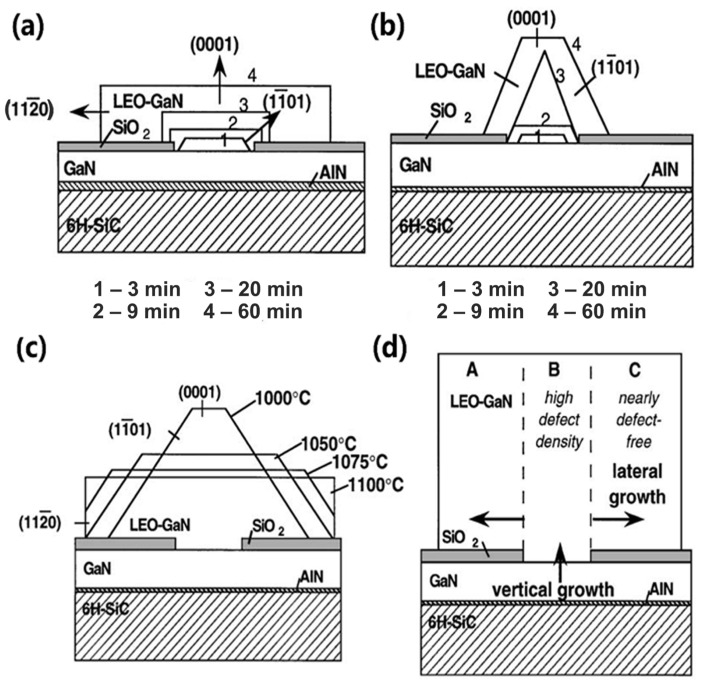
(**a**) SiO_2_ is used as a mask layer to realize the ELOG mechanism and the schematic diagram of material growth changes with time. The observation direction is [1$\bar{1}$00]. (**b**) SiO_2_ is used as a mask layer to realize the ELOG mechanism and the schematic diagram of material growth changes with time. The observation direction is [11$\bar{2}$0]. (**c**) SiO_2_ is used as a mask layer to realize the ELOG mechanism and the schematic diagram of material growth changes with temperature. The observation direction is [1$\bar{1}$00]. (**d**) A schematic diagram of the ELOG in a selectively grown GaN stripe. Reproduced with permission [178]. Copyright 2001, Elsevier Publishing.

Epitaxial lateral overgrowth (ELOG) is a low-defect-density group-III nitride material growth method that effectively suppresses the extension defect density. Relevant studies have shown that its dislocation density can be reduced to a maximum of one ten-thousandth of the initial value [178,179,180]. When the group-III nitride material overgrows transversely, the dislocation will change its direction, reduce the extension to the plane, and even appear as a closed-loop annihilation phenomenon so as to achieve the purpose of restraining defects. In order to realize the ELOG mechanism, some methods have been reported. For example, the AlGaN crack layer is grown on the GaN template, and the ELOG mechanism is realized by burying the crack [181]. Another example is to embed thin silicon dioxide (SiO_2_), antimony nitride (TiN), silicon (Si), or other materials as a mask layer and then etch a periodic window on them to realize the ELOG mechanism [178,179,180], as the relevant schematic diagrams shown in Figure 12. With this method, the mask layer is grown after the conventional growth of several microns of materials, which can additionally block the propagation of dislocations to the surface. Subsequently, the periodic window is etched on the mask layer until the underlying material is exposed. As the material continues to grow, since the nucleation energy of the group-III nitride material on the mask layer is much greater than that of the group-III nitride material, the material preferentially grows in the window region. When the growth thickness of group-III nitride material is greater than the thickness of the mask layer, group-III nitride material starts to grow laterally while growing vertically until it merges with group-III nitride material grown from the adjacent window to form a continuous layer. Finally, the ELOG mechanism is achieved, and the defects are suppressed. In the follow-up research, it was found that the degree of inhibition of this method on defects is affected by the type of mask layer material, the thickness of the mask layer, the shape and position of the window, and the growth environment of the material [61]. However, the adoption of this structural approach also brings increasing challenges, such as the ineffectiveness of large-scale devices and the introduction of impurities in the growth mask layer. In addition, the growth of group-III nitride materials on patterned surfaces to reduce defect density is also based on this principle, such as on PPS. Researchers have grown epitaxial AlGaN materials on striped substrates and other patterned surfaces. Facts have proven that dislocation density is significantly reduced [182]. In order to further explore the principle of the ELOG mechanism growing on patterned surface materials to restrain dislocations, Nie et al. found that, in addition to the critical factor that patterning provides space for lateral epitaxial growth, the edge dislocation density first decreases and then increases with the increase in growth temperature, while the screw dislocation density monotonously decreases [183]. What attracts people’s attention is that Chao et al. combined the masking method with the patterned substrate method to realize the ELOG mechanism, improved the material quality and LEE (which will be discussed in the following chapters), and finally improved the LED performance [184].

### 3.3. Nanowire and QD Structures

Ants do not know how high the sky is, just like humans cannot control time. The quest for different dimensions has always been fascinating.

#### 3.3.1. Nanowire Structure

##### Growth Method

**Figure 13:**
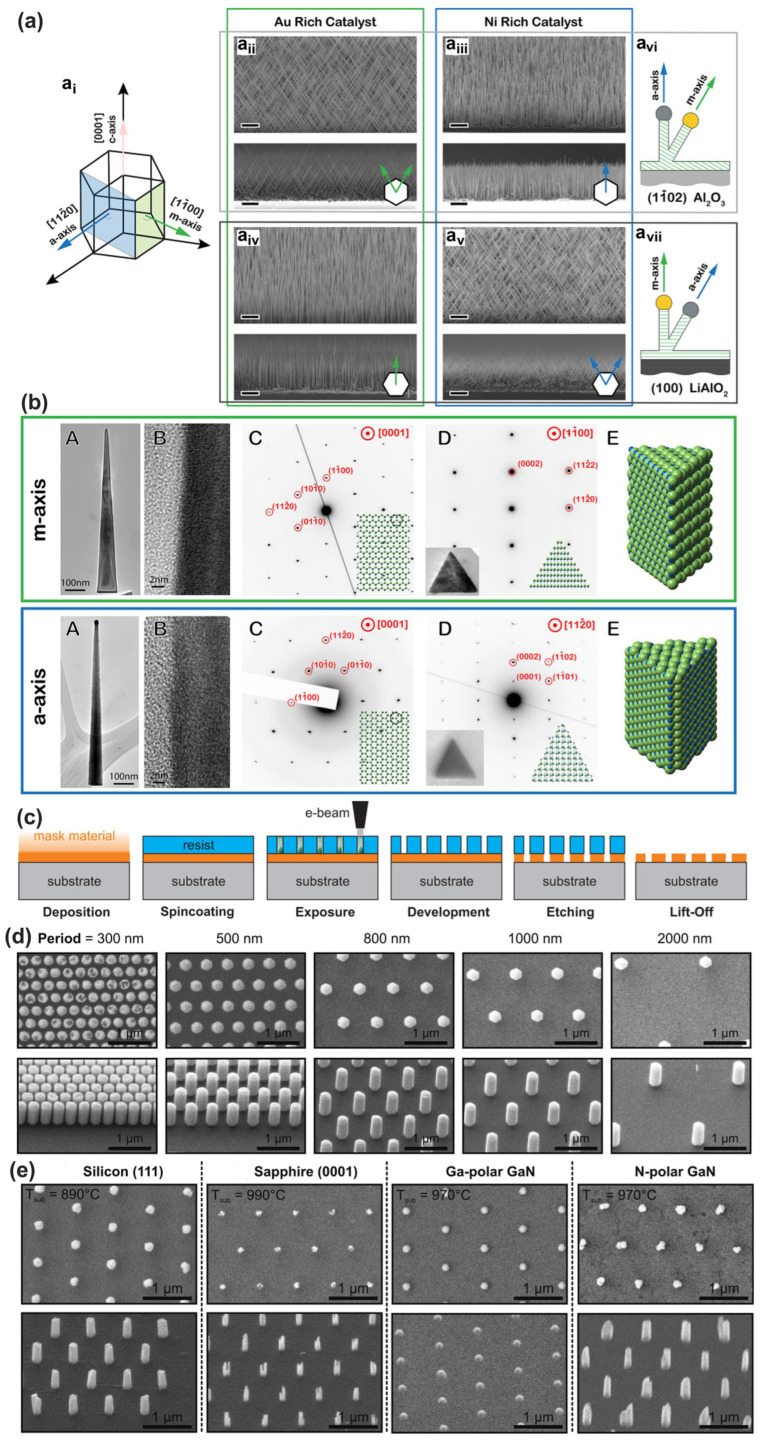
(**a**) Scanning electron microscope (SEM) images of nanowire arrays grown with different substrates and catalyst composition. Among them, (**a_i_**) schematically shows the crystallographic growth directions of a-, m-, and c-axis for hexagonal GaN nanowires. (**a_ii_**,**a_v_**) show SEM images of GaN nanowire arrays: m-axis GaN nanowires on r-plane α-Al_2_O_3_ (**a_ii_**), a-axis GaN nanowires on r-plane α-Al_2_O_3_ (**a_iii_**), m-axis GaN nanowires on (100) γ-LiAlO_2_ (**a_iv_**), a-axis GaN nanowires on (100) γ-LiAlO_2_ (**a_v_**–**a_vii_**) summarize the observed nanowire growth directions. (**b**) Transmission electron microscopy characterization of individual m-axis (**top**) and a-axis (**bottom**) GaN nanowires. (**A**) Low magnification image. (**B**) Lattice-resolved image of GaN nanowires. (**C**) Electron diffraction patterns were taken perpendicular to the growth axis, along the [0001] zone axis. The inset illustrations show the nanowires’ real space crystal structure and orientation with respect to electron diffraction. (**D**) Electron diffraction patterns of the nanowire cross-sections taken with the zone axis along the nanowire growth direction. (**E**) Isometric space-filling model of the nanowire. The c plane is facing left. Reproduced with permission [189]. Copyright 2014, ACS Publishing. (**c**) Illustration of the nano mask fabrication process by electron beam lithography. The mask also allows controlled nitrogen implantation to form nitrogen-vacancy centers in the diamond substrate prior to GaN nanowire selective area growth (SAG). (**d**) Top-view and tilted-view SEM images (45°) of selective area growth nanowire arrays with different periods. (**e**) SAG nanowires grow on different substrates. Reproduced with permission [190]. Copyright 2015, ACS Publishing.

Manufacturing LEDs based on nanowire structures has been a trend for the past decade, with the earliest research on nanowires dating back to the 1960s [185]. Thanks to the researchers’ contributions, the fabrication technology of group-III nitride nanowires is becoming more and more mature. Nowadays, the fabrication methods of III–V nitride nanowires are mainly divided into top-down etching methods and bottom-up growth self-assembled methods. The former is to perform a downward selective etching process on the surface of the existing material structure system to obtain a nanowire structure to enhance device performance. However, it strongly depends on the quality of the starting bulk material and introduces unnecessary etching damage and surface contamination, which is not worth the loss. The bottom-up growth method is affected by the bottom starting material and the growth environment, which has a more flexible operation method to grow nanowire structures that meet the application requirements, arousing the interest of researchers [186]. Bottom-up self-assembled III–V nitride nanowires grown by MBE, MOCVD, and HVPE, including catalyst-assisted growth and catalyst-free growth. The catalyst-assisted growth method mainly relies on the principle of the gas–liquid–solid (VLS) or gas–solid–solid (VSS) nucleation model (the former catalyst is liquid, and the latter is solid); that is, catalyst droplets or particles (Au, Ni, etc.) accelerate the chemical reaction of the gas source, nucleate nanowires at the liquid–solid or solid–solid interface, and promote their growth. The limitations of catalyst-assisted growth are also obvious. One is that the growth temperature of group-III nitrides can only occur within a limited temperature that is thermodynamically stable for additional catalysts, and the other is that impurity metal doping into the nanowires interferes with the radially active region (core–shell structure) reduces the luminous efficiency. In addition, since the orientation of nanowires grown by catalysts is strongly dependent on the host material and catalyst, although the growth position is relatively neat by means of the patterned catalyst arrangement method, the non-vertical growth of the grown nanowires cannot be avoided, and uniform lengths and radii are difficult to control precisely [187,188,189]. Figure 13a,b show the relevant experimental results. As the demand becomes more demanding, the above reasons limit its progress towards the mass production of high-end LED chips. In order to meet the product performance requirements and improve the accuracy of nanowire diameter, density, etc., self-assembled catalyst-free regioselective growth methods have been continuously explored. To put it simply, a patterned dielectric mask layer (the mask material is mainly SiO_2_, Si_3_N_4_ for MOCVD, and Ti, SiO_2_, Si_3_N_4_ for MBE) is deposited on the substrate, and under suitable growth conditions, the nanowires will grow only on the substrate in the window region [190]. Figure 13c–e show the relevant substrate pre-treatment and nanowire growth.

##### The Conditions of Nanowire Growth in a Selective Area

**Figure 14:**
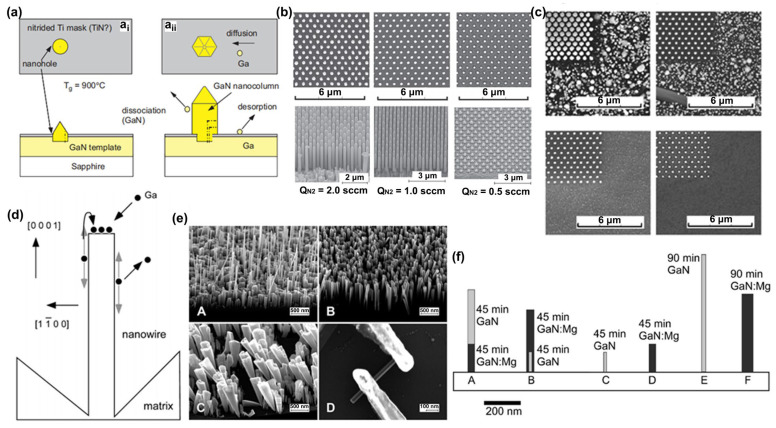
(**a**) Schematic diagram of the growth mechanism of SAG. Key elements of the growth scheme are the surface passivity of the nitrided Ti mask (**a_i_**), the diffusion and the desorption of Ga adatoms, and the dissociation of GaN (**a_ii_**). (**b**) SEM bird’s-eye views and top surface views of GaN nanocolumn arrays with 600 nm period triangular lattices grown with different nitrogen flow rates 2.0 sccm (**left**), 1.0 sccm (**middle**), and 0.5 sccm (**right**), at a temperature of 900 °C. (**c**) SEM views of random and spontaneous nucleations of GaN on nitrided Ti-mask surface outside nanohole patterns with different nitrogen flow rates 3.5 sccm (**above left**), 2.0 sccm (**above right**), 1.0 sccm (**lower left**), and 0.5 sccm (**lower right**) at a temperature of 900 °C. Reproduced with permission [191]. Copyright 2009, Elsevier Publishing. (**d**) Schematic of differential sticking coefficient mechanisms for spontaneous nanowire growth in MBE. Reproduced with permission [195]. Copyright 2008, Elsevier Publishing. (**e**) SEM images of the GaN nanowires. Nominally undoped sample on native Si substrate (**A**). Lightly doped sample on native Si substrate (**B**). Highly doped sample on native Si substrate (**C**). Single nanowire on host substrate, contacted by electron-beam lithography defined contacts (**D**). Reproduced with permission [200]. Copyright 2008, ACS Publishing. (**f**) Schematic diagram of GaN nanowire growth under different Mg doping conditions. Reproduced with permission [203]. Copyright 2006, ACS Publishing.

In terms of a suitable growth condition for nanowires, some important research progress made here needs to be highlighted. For the selective area growth of MBE, the plasma-assisted method pioneered by Kishino et al. has to be mentioned [191]. It successfully realized the selective growth of GaN nanowires in the window area of the patterned Ti dielectric mask layer treated by focused ion beam etching and studied the effect of environmental changes on the nanowires. It is found that adjusting the temperature to a suitable temperature ranging from 880 °C to 900 °C is beneficial in preventing the growth of the material on the mask layer and improving the morphological quality of the nanowire material. By varying the N_2_ flow rate, increasing the diffusion length of Ga atoms at low V/III ratios (by manipulations such as substrate heating) favors selective nucleation because it inhibits the surface nucleation of the Ti mask layer. As shown in the experimental data, it also exhibits anisotropy in the growth rate of nanowires with increasing nitrogen flux (Figure 14a–c show the relevant experimental results). Its pioneering research has greatly promoted the maturity of GaN nanowire growth by the plasma-assisted MBE method.

In essence, the artificial regulation of the growth rate anisotropy exhibited by nanowires in the plasma-assisted MBE method originates from the different growth mechanisms of Ga and N. The growth mode of nanowires is the Volmer–Weber mode, but there is no unified answer for the growth mechanism of plasma-assisted MBE. The currently generally accepted growth mechanism is that during the growth of nanowires, there is an additional Ga atomic diffusion behavior that occurs on the surface of the mask layer and the sidewalls of the nanowires. When the critical size is reached, Ga atoms in nanowires can either originate from direct collisional merger behavior or be collected from around the nanowire, while N atoms originate only from their merging growth behavior and proceed in the nanowire region where they collide [192,193]. Further, Debnath et al. pointed out that the adsorption–diffusion effect of sidewall atoms in thinner nanowires promotes their growth, while in thicker nanowires, the diffusion behavior of sidewall atoms has little effect, and instead, the top atoms directly merge and grow [194]. Bertness et al. also proposed their growth model, in which the difference in the adhesion coefficient of Ga atoms on the top and sidewalls of the nanowire is responsible for the growth of the nanowire. Since the nanowire top adhesion coefficient is greater than the sidewall adhesion coefficient, Ga atoms within the top collision and diffusion lengths will be incorporated into the nanowire, while most of the sidewall Ga atoms will not participate in the nanowire growth. The growth of nanowires is affected by both the adhesion coefficient and the atomic diffusion length, which can be regulated by adjusting the growth environment, such as temperature and source flux [195], as Figure 14d shows the relevant schematic diagram.

Regardless of the specific growth mechanism of plasma-assisted MBE, its growth is usually affected by the following aspects. The first is the choice of the nucleation temperature, which affects the Ga atomic diffusion length as described earlier. The specific temperature conditions for the specific selective nucleation also depend on factors such as the mask layer and the substrate type, as well as the V/III ratio. The conditions required for selective nucleation are not fixed, but generally, the optimal selective nucleation temperature increases with increasing N flux at Ga-rich [196,197]. Unfortunately, there is a need for more literature on the effect of Ga flux on the selection of suitable nucleation temperatures for N-rich. Second, for the V/III ratio of nanowire growth, there are two cases when the growth time and temperature are constant. The related data showed that under N-limited conditions, the increase in N flux promoted both radial and axial growth, but the promotion was gradually weakened. Under N-rich conditions, with the increase in Ga flux, the radial growth of nanowires is promoted, the nanowire density and coverage are also improved, and the axial growth rate increases rapidly and then tends to be saturated [198,199]. The third is the effect of the period of the nanowire array, which shows that a considerable number of nanotubes are generated when the period is short, and the nanotube structure gradually disappears with the increase in the period. Finally, dopants also have a great influence on the growth morphology of nanowires. For MBE growth, a high concentration of Si n-type doping leads to a decrease in nanowire density, an increase in slanted nanopillars, and a progressively larger diameter during the growth of individual nanowires. Correspondingly, with the increase in p-type Mg doping, the aspect ratio of the nanowires becomes smaller, their diameter becomes larger, and the number of triplets twinned domains increases [200,201,202], as Figure 14e shows.

**Figure 15:**
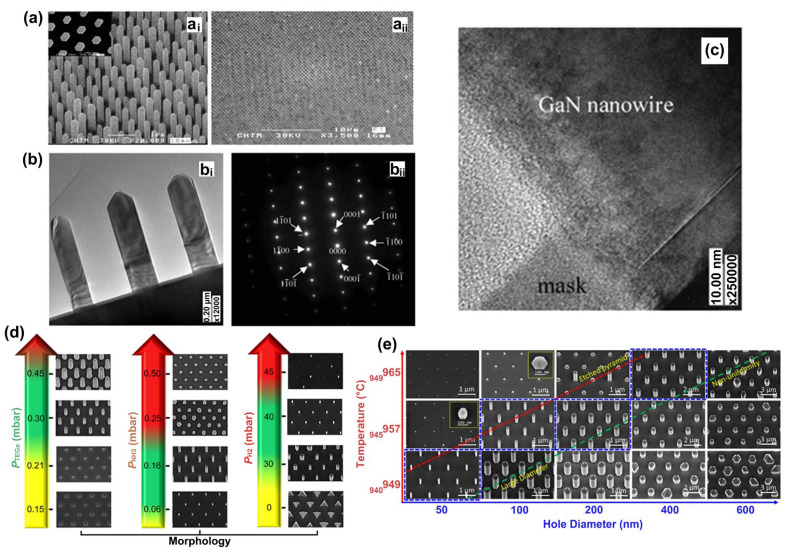
(**a**) SEM of a GaN nanowire array consisting of 1 µm GaN nanowires grown by MOCVD (**a_i_**). A lower-magnification SEM image reveals the long-range order of the GaN nanowire arrays (**a_ii_**). (**b**) The TEM image shows the nanowires’ vertical sidewall and top crystallographic facets (**b_i_**). The electron diffraction pattern confirms the GaN nanowires’ single-crystal nature (**b_ii_**). (**c**) The TEM image shows one side of a single GaN nanowire as it emerges (**bottom-right** to **upper-left**) from the growth mask. The growth mask is at the lower left of the image. Reproduced with permission [201]. Copyright 2008, AIP Publishing. (**d**) Morphology of GaN nanostructures with respect to different pressures (P), including P_TEGa_, P_NH3_, and P_H2_, highlighting the insufficient (yellow), optimal (green), and excessive (red) values of P_TEGa_, P_NH3_, and P_H2_. (**e**) Morphology of Ga-polar GaN nanostructures at different growth temperatures for different hole diameters grown by MOCVD. Reproduced with permission [208]. Copyright 2022, ACS Publishing.

In order to meet the requirements of large-scale production, MOCVD is the main productive force for the growth of group-III nitride materials. To this end, researchers have made unremitting efforts to explore the application of MOCVD to fabricate GaN nanowires successfully and have carried out many experiments on the growth mechanism and growth control (Figure 15a–c show GaN nanowires grown by MOCVD). Hersee et al. pioneered the pulsed MOCVD growth mode, which was successfully applied to fabricating GaN nanowire structures [203] (Figure 14f shows the relevant diagrams). In this mode, one pulse period can be divided into four stages, namely the nitrogen source injection period, the nitrogen source interruption period, the gallium source injection period, and the gallium source interruption period. During the nitrogen source implantation, the nitrogen source reacts with the gallium source of the previous cycle to grow the nanowires. Subsequent interruption of the nitrogen source can avoid the occurrence of the film growth mode, and the H_2_ generated by the nitrogen source reaction can be evacuated to reduce the probability of H-N passivation bonds, which promotes the growth of nanowires. The turn-off time of the Ga source after the Ga source implantation and before the nitrogen source implantation determines the lateral growth of the nanowires. During this period, due to the difference in the adhesion coefficient of Ga atoms on the top and sidewalls, proper manipulation can change the nanowire aspect ratio. Lin et al. systematically investigated the effects of off-time, temperature, and NH_3_ flow rate on GaN nanowires under pulsed-mode MOCVD growth conditions [204]. The relevant conclusions can be summarized into the following four points: a short turn-off time of the Ga source is beneficial to improve the lateral to vertical ratio; a long Ga source interruption time is conducive to the formation of narrow and long nanorods; with the increase in temperature, vertical growth is promoted, and lateral growth is suppressed, which is because the difference between the desorption rates of Ga atoms on the m-plane and the c-plane gradually increases during the process; the increase in NH_3_ flow rate promotes lateral growth and inhibits vertical growth in N-enriched conditions. However, the higher H_2_/N_2_ ratio does not further promote the growth of nanowires; the uniformity of the nanowire arrangement will deteriorate, and the diameter will be narrowed.

Pioneeringly, Bergbauer et al. applied continuous flux mode MOCVD to realize the selective growth of GaN nanowires on a sapphire substrate with SiO_2_ as the mask layer, which opened another door for the growth of GaN nanowires [205,206]. In the continuous flux growth mode with the carrier gas H_2_/N_2_ ratio as the decisive factor, when the H_2_/N_2_ ratio was changed from 0 to 2, the nanowire growth morphology transitioned from only observed pyramidal structures to good quality and progressively taller nanowires. Additionally, nanowires with different polarities exhibit different surface morphologies. This can be explained by the profoundly different effects of H_2_ on the growth morphology of nanowires with different polarities [207]. Due to the difference between the r-plane and c-plane terminal atoms at the top of the N-polar and Ga-polar nanowires, taking N-polarity as an example, the c-plane is N atoms, and the r-plane is Ga. Due to the N-H passivation of the c-plane bond and the r-plane dangling bond being larger than the m-plane, H_2_ will corrode the top r-plane of N-polar nanowires and eventually appear in a flat-topped shape. Similarly, the tops of Ga-polar nanowires are mostly gold tower structures. Deeper thinking here would also explain why a low V/III ratio favors the vertical (c-plane) growth of N-polar nanowires and why a very high H_2_/N_2_ ratio shortens the nanowire diameter without further promoting nanowires’ growth. Recently, Adhikari et al. investigated the effects of temperature and pressure parameters on GaN nanowires growing in a selected area by MOCVD, which provides a valuable reference for the uniform and controllable growth of nanowire arrays [208] (Figure 15d,e show the relevant experimental results).

##### Advantages and Challenges of Nanowire LEDs

**Figure 16:**
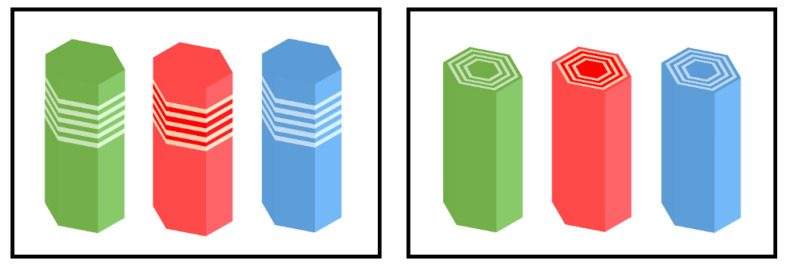
Diagrams of vertical structure nanowires (**left**) and core–shell structure nanowires (**right**).

At present, there are two main types of LEDs based on nanowire structures: one is a core–shell structure, which uses n-type GaN as the core and p-type GaN as the shell and grows an active region in the middle; the second is a vertical structure in which different material structures are stacked layer by layer in a columnar shape, as Figure 16 shows [209]. It is not difficult to understand the growth methods and differences between the two structures of nanowire LEDs through the above introduction to the regulation of nanowire growth. However, it is worth noting that in the core–shell structure, since p-type doped Mg promotes lateral growth and n-type doped Si inhibits lateral growth and enhances vertical growth, the positions of p-type and n-type materials are relatively fixed [210]. In addition, the surface morphology of the core–shell structures of GaN nanowires with different polarities is also very different. The top of the N-polar core–shell structure is flat, while the top of the Ga-polar core–shell structure has an accumulation of heterogeneous materials [211].

More advancement must be driving new things to be applied. The LED of the nanowire structure has some advantages compared with the LED of the planar structure [212,213,214,215]. These advantages are summarized as follows: (1) The strain transmitted from the substrate gradually disappears with the growth of the height of the nanowire (usually, the height equals the diameter of the nanowire when the strain disappears). (2) Minor strains in nanowires can dramatically reduce QCSE (vertical structure nanowire LED). More than that, the sides of the nanowires are non-polar, which can almost eliminate the influence of QCSE (core–shell structure nanowire LED). (3) The heterogeneous structure of nanowires makes them more resistant to losing matches. It is specifically reflected in the thermal expansion coefficient of the lattice constant, and its phenomenon can be interpreted as the generated stress being easy to relax on the surface. (4) Since the nanowires are more adaptable to strain, this means that there are few defects in the MQWs structure even with high indium doping, which promotes the extension of the emission wavelength of group-III nitride LEDs to longer (vertical structure nanowire LED). (5) The light-emitting area of the LED with a core–shell structure nanowire is about ten times larger than that of the LED with a planar structure, which can effectively improve the efficiency of light extraction and decrease the local current density. (6) The LED droop effect of the nanowire structure is weaker, and its advantage is more obvious with the increase in the indium composition in the quantum well. (7) P-doped nanowires have a higher hole concentration [216].

However, nothing is absolutely perfect in the world, and operating at the nanoscale range naturally brings some challenges. (1) The large surface area has a high density of surface states, and the pinning of the Fermi level on the surface will cause the nanowire band to bend, which leads to the separation of electron–hole pairs and reduces the luminous efficiency [217]. SRH-induced nonradiative recombination reduces quantum efficiency [218]. (2) Also, sidewall defects are prone to occur when coalescence occurs during nanowire growth [219]. (3) What is more, improving the indium or aluminum composition of m-plane InGaN/GaN or AlGaN/GaN quantum wells and inhibiting non-radiative recombination in the active region for high efficiency is also a challenge [220]. (4) In addition, the most important challenge is the lack of understanding of its physical mechanism, which is mainly due to the lack of relevant characterization methods, leading to a lack of systematic research.

Unfortunately, persuasive research solutions to some of these challenges are still missing. Here some compelling solutions will be presented. The key to solving challenge (1) is the development of correlated passivation processes to inhibit the surface state as well as other surfaces’ non-radiative recombination. At present, relevant studies have shown that the correlated passivation processes with octadecyl thiol or KOH as passivating agents have proved effective [221,222]. Challenge (2) brings the problem of non-radiative recombination at the surface and electron leakage, resulting in efficiency droop. At present, there is a lack of breakthrough research on how to prevent coalescence from the root. Therefore, at present, ideas on how to reduce the surface of non-radiative recombination and reduce the sidewalls’ leakage have mainly been reported. Grenier et al. achieved a crack-free core–shell LED emitting at 280 nm by growing an AlGaN gradient layer and a cladding layer on the sidewall of nanowires and reducing the thickness of the quantum wells and found that the threshold strain energy density is about 4 J/m^2^ [223]. Along the way, they continued to refine the previous idea by growing gradient layers and cladding layers on both sides of the sidewall MQWs instead of just on one side. Relevant works have greatly promoted the application of UV core–shell LEDs [224]. And similar approaches have proven effective in the field of visible core–shell LEDs by adding an AlGaN layer [225] (refer to Kapoor et al.’s research article for the mechanisms involved [226]). For reducing non-radiative surface recombination, we can refer to the relevant solutions in Challenge (1). The solution for (3) should be similar in relevance to the relevant solution for planar MQWs. And the red luminescence cases of nanowire LEDs with a high indium component can be found in Section 4.4.1. In theory, nanowires have inherent advantages in solving (3) compared with planar layer structures. However, contrary to one’s wishes, its efficiency is still far from that of the planar layer structure. It is worth noting that Ra et al. fabricated an ultraviolet-emitting core–shell structure nanowire LED with an IQE of 74% at a wavelength of 318 nm, which is close to the efficiency of ultraviolet LEDs near this wavelength of planar structures [227]. Does this imply that nanowire structures are more useful for UV luminescence than visible light luminescence? Continuous research is necessary. By increasing the growth temperature of the GaN barrier layer and adding an AlGaN spacer between the quantum well and the quantum barrier in each period, Lu et al. found that the indium component of the InGaN quantum well becomes uniform and the diffusion of point defects to MQWs decreases [228].

It is believed that Challenges (1), (2), and (3) can be solved easily after mastering relevant physical mechanisms and laws. After realizing the urgency of the Challenge (4), some mechanisms and theories have been explored in recent years. Hasan et al. explored the effect of quantum well thickness on the optical properties of GaN nanowires in vertically structured nanowires [229]. Park et al. explored the c-plane and a-plane luminescence properties of nanowires. It is found that the luminescence intensity of the a-plane is positively correlated with indium content in the range of 0.2 to 0.3. It is stronger than that of the c-plane, for which it is negatively correlated with indium content. The luminescence intensities of both the c-plane and a-plane are negatively correlated with the size of nanowires [230]. Adhikari et al. studied the mechanisms of incorporation of the aluminum component into core–shell nanowires and the relationship between the aluminum component and defects. It is found that N_2_ can reduce the diffusion-length difference between Al and Ga atoms and smooth the surface of the m-plane. With the increase in gas-phase Al sources, the Al incorporation rate decreases, defects increase, and the dislocation proportion increases [231]. This study helps to solve the difficulty of the related Al component increase in Challenge (3). Ries et al. investigated the spectral redshift origin and polarization patterns of InGaN/GaN MQWs in GaN nanowires. It is found that indium component inhomogeneity may be the main cause of redshift and polarization anisotropy [232]. It can be said that the development of and improvement in characterization methods are the keys to new physical discoveries. Segura-Ruiz et al. developed a characterization technique known as time-resolved X-ray excited optical luminescence microscopy, which can be successfully used to explore charge carrier dynamics in core–shell nanowire LEDs [233]. Furthermore, Park et al. obtained the strain distribution images of nanowires using scanning transmission electron microscopy and dark-field inline holography [234]. Although some physical mechanisms and laws have been explored, relatively speaking, it is difficult to form a systematic theory. There are still some doubts about some mechanisms, such as the three-dimensional transfer mechanism and distribution of charge carriers in nanowire LEDs, the loss mechanism of charge carriers, the three-dimensional internal heat distribution, and the p-type doping enhancement mechanism. Nowadays, light-emitting diodes based on nanowire structures have become an important part of nanolight emitting diodes to achieve multi-color luminescence and others with the application of innovative structures such as QDs, nanodisks, composition gradient layers, and graphene substrates. At the nanoscale, it is believed that more pioneering structures, such as GaN nanotubes and nanorings, will be created in the near future.

#### 3.3.2. QDs

In the past two decades, research on QDs has gradually risen and become increasingly popular. In general, the application of QDs is mainly aimed at improving light emission efficiency, such as by providing color conversion functions. Taking the research focus of group-III nitride QDs in visible and invisible light as an example, the properties of QDs are introduced in this chapter. Of course, they are not separate, but they are connected.

##### QDs for Ultraviolet and Infrared Luminescence

**Figure 17:**
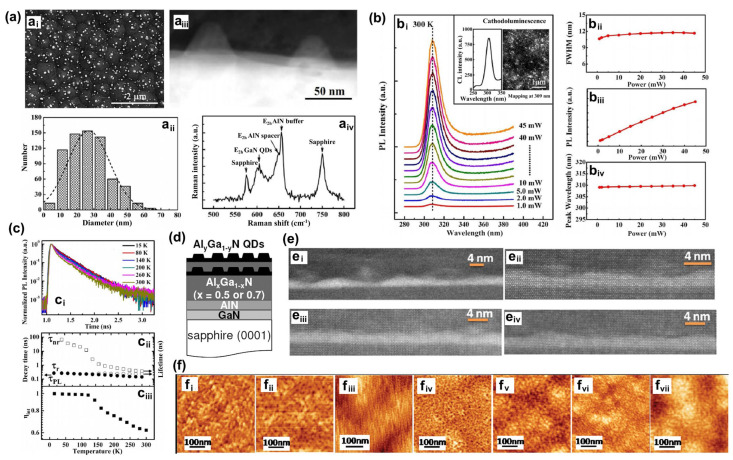
(**a**) SEM (**a_i_**), size distribution (**a_ii_**), cross-sectional TEM image (**a_iii_**), and Raman spectrum (**a_iv_**) of the self-assembled GaN/AlN QDs grown on n-Al_0.7_Ga_0.3_N, respectively. (**b**) Power–dependent PL was taken at room temperature. Power-dependent PL spectra of the GaN/AlN QDs at 300 K. Inset show the cathodoluminescence (CL) spectrum and monochromatic CL mapping at a wavelength of 309 nm at 300 K (**b**_i_). Spectral width (**b_ii_**), integrated intensity (**b_iii_**), and the peak wavelength of the PL spectrum (**b_iv_**) as a function of excitation power, respectively (**right**). (**c**) Temperature-dependent time-resolved photoluminescence (TRPL) taken in temperatures ranging from 15 K to 300 K. TRPL spectra for GaN/AlN QDs as a function of temperature (15–300 K) (**c_i_**). Temperature-dependent decay time (closed circles), radiative (open circles), and nonradiative lifetimes (open squares) (**c_ii_**). Temperature-dependent PL efficiency (closed squares) (**c_iii_**). Reproduced with permission [244]. Copyright 2014, Springer Nature Publishing. (**d**) Schematics of the sample structure with different Al components of QDs and cladding layers. (**e**) High-angle annular dark-field imaging in scanning TEM mode images of Al_y_Ga_1−y_N QDs grown in an Al_0.7_Ga_0.3_N (0001) cladding layer for an Al composition y equal to 0.1 ((**e_i_**), sample C), 0.2 ((**e_ii_**), sample D), 0.3 ((**e_iii_**), sample E), and 0.4 ((**e_iv_**), sample F). (**f**) AFM images (500 × 500 nm^2^) of Al_y_Ga_1−y_N QDs for the complete sample series (**f_i_**–**f_vii_**). Reproduced with permission [245]. Copyright 2019, AIP Publishing.

The earliest theory of QDs dates back to the 1980s, and reports of self-growing group-III nitride QDs following the Stranki–Krastanov (S-K) model have been made since the 1990s [235,236]. A growth method to control the size of GaN/AlN QDs was proposed by Ramvall et al., which is weaker than the traditional S-K growth mode, and they systematically explain the quantum confinement mechanism in GaN QDs [237]. UV LED based on GaN/AlGaN/AlN QDs S-K growth mode has been widely studied, which plays an influential role in medical and environmental engineering. Here, several UV LED QDs growth methods based on planar and nanowire structures are introduced for references [238,239,240,241,242,243]. Improving quantum efficiency has always been the focus of research for the ultraviolet luminescence of QDs. To this end, researchers have carried out a series of studies. Yang et al. achieved an IQE of 62% at room temperature at 300 nm wavelength by growing GaN/AlN high-density QDs using MOCVD [244] (Figure 17a–c show the relevant experimental results). However, the quantum efficiency based on AlGaN QDs is at a low level in the shorter wavelength segment. Currently, it is reported that 5% IQE can be achieved at 276 nm at room temperature [245] (Figure 17d–f show the relevant experimental results). In terms of infrared luminescence, it has been dominated by phosphide and arsenide LEDs for a long time. However, it has problems in a high-temperature environment. In order to overcome this obstacle, InN QDs that overcome the huge lattice mismatch between GaN and InN materials can be qualified. The growth method of InN QDs was reported in detail nearly 20 years ago [246,247]. For articles on the growth, properties, and applications of InN QDs, especially LEDs, please refer to the review and research articles by Reilly et al. and Ahmad et al. [248,249,250,251].

##### QDs for Visible Luminescence

The luminescent wavelength of the InGaN/GaN QD LED is mainly in the visible region, and its application in lighting, display, VLC, and so on has brought a qualitative leap. InGaN QDs grown based on the S-K growth mode have been widely used and reported, which will not be described in detail here. Please refer to the review article by Wang et al. [252]. QDs growth, considered novel and impressive here, will be introduced. Chen et al. proposed a feature that the growth of low-temperature GaN on passivated high-temperature GaN with high surface transition energy can induce a dot material growth pattern, which opened up a new growth method for InGaN/GaN QDs that had some differences from the previous ones [253]. Of course, the combination with some novel processes, such as photochemical etching to precisely control the size of QDs to achieve the 10 nm size range, has also been reported [254].

**Figure 18:**
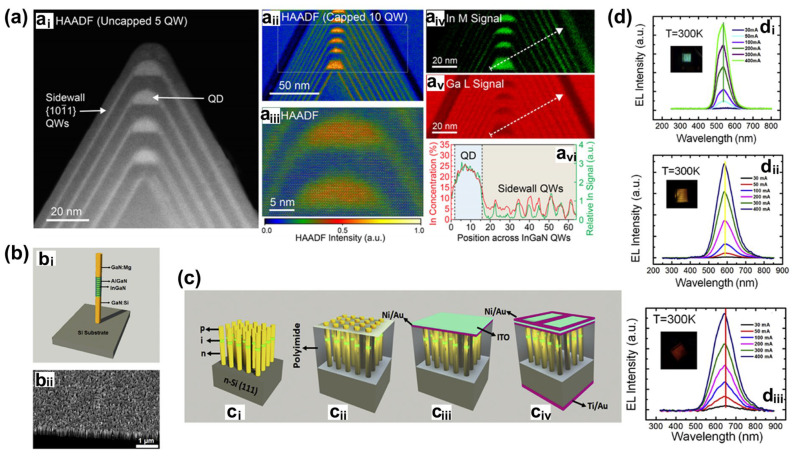
(**a**) Cross-sectional high-angle annular dark-field (HAADF) scanning TEM image of the spatially confined dot-in-pyramid structure in (**a_i_**) the uncapped 5 QW sample and (**a_ii_**) the capped 10 QW sample. (**a_iii_**) High magnification HAADF image of the dot-like features. Simultaneously acquired (**a_iv_**) In and (**a_v_**) Ga electron energy loss spectroscopy (EELS) elemental maps of the boxed region in (**a_ii_**) indicating high In-content at regions of V-pit intersections. (**a_vi_**) The relative In signals extracted across the line scan from the In map in (**a_iv_**) and the quantitative In compositions calculated across the line scan from the Ga map in (**a_v_**). Reproduced with permission [257]. Copyright 2021, ACS Publishing. (**b**) Schematic illustration of an InGaN/AlGaN dot-in-a-wire LED heterostructure (**b_i_**). An SEM image showing the morphology of the InGaN/AlGaN dot-in-a-wire heterostructures grown on a Si substrate by MBE (**b_ii_**). (**c**) Fabrication flow of InGaN/AlGaN nanowire LEDs. The nanowire LED sample (**c_i_**) was fully covered with polyimide resist, followed by oxygen plasma dry etching to expose the top of nanowires for metallization (**c_ii_**). Top p-contact with Ni/Au/ITO was deposited on the nanowire surface (**c_iii_**). Ni/Au and Ti/Au were deposited on top of ITO and backside (**c_iv_**). (**d**) Room-temperature electroluminescence spectra under different injection currents for green (**d_i_**), yellow (**d_ii_**), and red (**d_iii_**) nanowire LEDs. Reproduced with permission [263]. Copyright 2017, Elsevier Publishing.

To achieve red light emission from GaN-based LEDs, InGaN QDs with indium-rich components are one of the essential solutions. An In-rich InGaN QD growth method was reported in 2004 to improve the luminescence efficiency and shift the luminescence peak to a long wavelength [255]. The development of InGaN QD growth based on the planar LED to achieve phosphor-free indium-rich red luminescence has always attracted people’s attention. In recent years, indium-rich InGaN QD planar LEDs have made continuous breakthroughs. Yang et al. realized white light emission without phosphor by combining green-yellow QDs with blue quantum wells to form an integral LED [256]. Although the luminescence of QDs did not reach the standard of red light, this was of enlightening significance. Chung et al. demonstrated the feasibility of growing indium-rich QDs using V-shaped pits generated by epitaxial material on a Si substrate to achieve longer wavelength emission at the apex of the pyramid structure they formed [257] (Figure 18a shows the relevant experimental images). The formation of indium-rich QDs induced by high-density V-shaped pits and their carrier localized effects have also been explored [258]. Zhang et al. used plasma-assisted MBE to explore the growth of red QDs by adjusting the growth parameters and obtained InGaN QDs from 460 nm to 622 nm, from green light to red light [259]. However, it was still difficult for QDs grown by planar LEDs to achieve highly efficient red luminescence.

**Figure 19:**
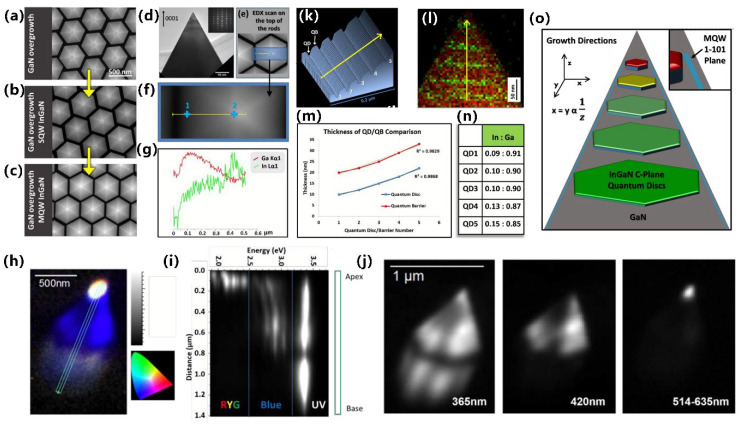
SEM imaging of (**a**) GaN nanorod overgrowth with truncated apex tips, (**b**) InGaN/GaN SQW growth on (**a**) with a smaller c-plane diameter top, (**c**) InGaN/GaN MQW growth on (**a**) with a sharp apex tip, (**d**) TEM of the sharp tip on an individual nanorod with selective area electron diffraction (SAED) inset showing the wurtzite hexagonal structure with the [0002] growth direction, (**e**,**f**) energy-dispersive X-ray (EDX) line and point scan in SEM mode, (**g**) spectra from the line scan in (**e**,**f**), (**h**) real color representation of the spatially resolved cathodoluminescence (CL) emission, (**i**) spectral information extracted along the line in (**h**), (**j**) spatially resolved CL of the nanorod at increasing wavelength range, (**k**) surface area plot of the 5 × MQW/quantum barriers (QBs) along the yellow line, (**l**) the EDX mapping of the 5 MQWs, (**m**) graphical plot of the QD/QW thickness data obtained from (**k**), (**n**) table of the In: Ga ratio data at each MQD, (**o**) schematic illustration of the changing thickness for the InGaN wells in all three x, y, z directions at the c-plane. Reproduced with permission [260]. Copyright 2016, RSC Publishing.

With the improvement in nanowire manufacturing levels, the idea of combining epitaxially grown InGaN QDs with nanowires to improve indium doping has been widely accepted. Therefore, researchers are constantly seeking performance breakthroughs in this method. Conroy et al. grew a quantum disk structure similar to QDs at the end of the tip of the nanowires and manipulated the limiting size of the quantum disk by position selection to achieve red luminescence without indium component increase [260], as shown in Figure 19. InGaN/GaN QDs based on the Volmer–Weber growth mode of vertical structure nanowires have also been reported in the last decade [261]. Subsequently, the successful self-growth of InGaN/GaN QDs on the m-plane and the exciting increase in indium composition greatly stimulated the development of single photon emission diodes [262]. Philip et al. introduced an InGaN/AlGaN dot-in-a-wire red luminescence scheme, and their research results showed high IQE and strong resistance to efficiency droop [263]. Figure 18b–d show the corresponding electroluminescence and nanowire structure diagrams. Recently, Um et al. reported the growth of InGaN QDs on GaN nanowires in pulsed MOCVD mode [264]. Compared with core–shell multi-quantum well nanowires, this method increases the indium content and successfully makes the FWHM of long-wavelength luminescence below 100 nm. One of the most notable is that they observed an increase in the indium component in nanowires and QD structures, which is most likely a way to achieve color conversion even though its emission range is in the infrared band. Unfortunately, these approaches have not yet met people’s expectations in industrial production currently. Instead, colloidal QDs of other materials have been combined with planar LED quantum well structures or nanowire LEDs to achieve phosphor-free red-light emission. The application of colloidal QDs and other materials in the LED field can be referred to in the review article by Liu et al. [265,266]. Nonetheless, epitaxy-grown InGaN QDs are still highly anticipated as one of the most efficient implementations of the ideal full-color scheme, awaiting a technological breakthrough.

##### Properties-Based: Why We Chose QDs

The reason why scientists are so persistent with group-III nitride QDs can be attributed to the fact that QDs are so advanced. Methods as to how one might further self-growing group-III nitride QDs can improve LED performance in other ways will be described here. (1) The nanosize of QDs results in complete surface relaxation and strain release, which can further effectively reduce QCSE compared with other large-size group-III nitride material structures [267]. (2) QDs help reduce non-radiative recombination and thus improve IQE. The growth position of QDs is not affected by dislocation and has a strong carrier localization effect, which means that QDs can still grow at the dislocation, and they will compete for carrier-trapping behavior in the non-radiative recombination center at the dislocation so as to reduce the non-radiative recombination of carriers. In the same way, QDs at non-dislocation sites can also play a role in trapping prevention [268,269,270]. (3) Group-III nitride QDs have large exciton binding energies, increasing the probability of direct carrier recombination and reducing energy loss [271,272]. (4) The application of group-III nitride QDs has shown great potential to effectively solve the problems of GaN-based LED efficiency degradation and green gap [273,274]. (5) There is less concern about the quality of the QD material itself, and its properties mainly depend on the geometric size, so compared with other material structures, it is easy to operate to improve the photoelectric performance.

There are aspects that need to be known when we choose QDs to make LEDs based on other people’s research. Related studies show that the smaller the QD size, the greater the optical output power as well as the coupling between excitons and longitudinal phonons decreases [237,275]. The carrier recombination rate is negatively correlated with the height (thickness) and positively correlated with the radius. Exciton binding energy is negatively related to height and radius. The luminescence wavelength of QDs increases with height and radius [276].

### 3.4. P-Type Doping Technology

For a long time in the past, the p-type high-efficiency doping problem of group-III nitrides has plagued the fabrication of high-efficiency LEDs due to the high activation energy of Mg and the existence of electro-inactive Mg-H complexes in the doping process. In fact, research on GaN-based LEDs began as early as the 1970s, but practical GaN-based LEDs did not appear until the 1990s due to the barriers to the p-type doping technology of group-III nitrides [277]. Prior to this, Amano et al. obtained p-GaN by electron beam irradiation of doped Zn, which is far from practical application [278]. Finally, in 1992, Nakamura et al. used Cp_2_Mg as the dopant to explore the effect of annealing temperature on the activation of the dopant by high-temperature rapid annealing. Finally, they achieved high-efficiency p-type GaN doping, which became the cornerstone technology for GaN LEDs to become practical [279]. After continuous improvement, on the basis of traditional high-temperature thermal annealing, Sakurai et al. reported an ultra-high-pressure annealing scheme, which made the ratio of hole concentration in p-GaN to Mg dopant concentration more than 70%, greatly improving the activation rate [280]. But that is not enough, especially in the field of UV AlGaN-based LEDs; with the deepening of Mg activation energy in high-Al composition AlGaN materials, the average p-type Mg doping is difficult to meet the requirements of efficient and practical carrier doping concentration, so it is urgent to propose a new p-type doping technology.

#### 3.4.1. Semiconductor Superlattice Doping

**Figure 20:**
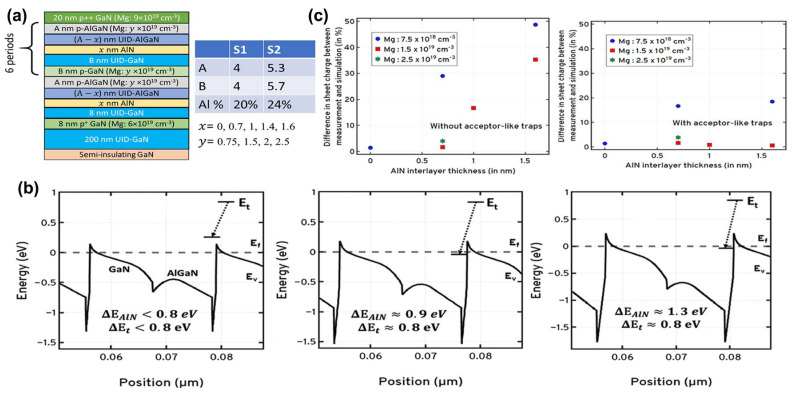
(**a**) Schematic epitaxial structure of the grown N-polar modulation-doped GaN/AlN/AlGaN SLs. (**b**) Actually, the simulated valence band diagram accounting for acceptor-like traps at positive polarization interfaces with different AlN interlayer thicknesses of 0.7 nm, 1 nm, and 1.6 nm from left to right, respectively (Mg-doped). (**c**) The difference in the sheet charge between measured and simulated values (in percentage) as a function of AlN interlayer thickness for samples with different Mg doping values, without invoking the acceptor-like trap hypothesis (left) and invoking the acceptor-like trap hypothesis. Introducing acceptor-like traps of 1 × 10^13^ cm^−2^ at the positive polarization interfaces allows for a substantial agreement of simulated and measured values. Reproduced with permission [284]. Copyright 2020, AIP Publishing.

**Figure 21:**
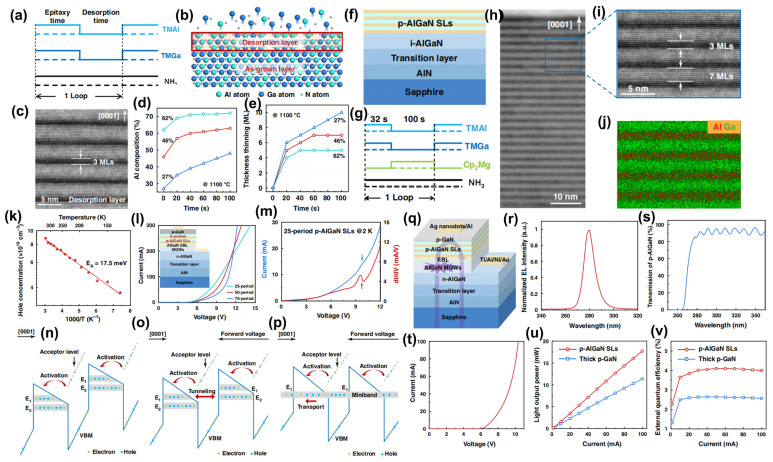
(**a**) Schematic process of the periodically interrupted epitaxy. (**b**) Schematic illustration of the self-assembled bilayer structure by desorption-tailoring. (**c**) Z-contrast image of the self-assembled bilayer structures in AlGaN with the desorption time and initial Al composition of 80 s (at 1100 °C) and 46%, respectively. (**d**) Desorption time dependence of Al composition in the desorption layer. (**e**) Desorption time dependence of the thinning thickness in the as-grown layer. (**f**) Schematic illustration of the sample with desorption-tailored p-AlGaN SLs. (**g**) Schematic process of desorption-tailoring for p-AlGaN SLs. (**h**) HRTEM image of the p-AlGaN SLs. (**i**) Z-contrast image of the p-AlGaN SLs. (**j**) Distribution of Al and Ga atoms by EDS mapping corresponding to the region in panel (**i**). (**k**) Temperature dependence of the hole concentration in the desorption-tailored p-AlGaN SLs. (**l**) I–V curves at room temperature of DUV-LED structures (inset) with p-AlGaN SLs period of 25, 50, and 75, respectively. (**m**) I–V and corresponding dI/dV curves at 2 K of DUV-LED structure with 25-period p-AlGaN SLs. (**n**) The upward inclining of the p-AlGaN SLs energy band profile along [0001] direction at equilibrium. (**o**) Resonant tunneling between E_1_ and adjacent E_2_ when flattening the p-AlGaN SLs energy band profile along [0001] direction by applying a forward voltage. (**p**) Formation of minibands when applying a higher forward voltage. (**q**) Schematic illustration of DUV-LEDs with desorption-tailored p-AlGaN SLs. (**r**) EL spectrum (at 100 mA) of DUV-LEDs. (**s**) Transmission spectrum of the desorption-tailored p-AlGaN SLs (without the p-GaN contact layer). (**t**) I–V curve of DUV-LEDs with the p-electrode of complex Ag nanodots/Al. (**u**,**v**) Dependence of the LOP and EQE on the injection current for DUV-LEDs with desorption-tailored p-AlGaN SLs and thick p-GaN, respectively. Reproduced with permission [287]. Copyright 2022, Springer Nature Publishing.

In order to pursue a higher doping concentration, modulation-doped AlGaN/GaN semiconductor SLs have been studied and proven to enhance the activation of acceptors. Its piezoelectric and spontaneous polarization fields promote the deeper Mg acceptors in the doped AlGaN barrier to transfer to the GaN potential well to form a high-density two-dimensional hole gas [281,282]. In 1997, Nakamura et al. successfully grew LEDs, including p-type modulation doped superlattice structure on an ELOG substrate, and achieved ideal results to prove its feasibility [283]. Not long ago, Krishna et al. found an acceptor trap of about 0.8 eV on the positively polarized interface of the GaN/AlN/AlGaN superlattice, which can be regarded as a hole source [284] (Figure 20a–c show the relevant experimental results). Then, by systematically studying the effects of doping concentration, AlN thickness, and Al composition on the ionization and hole mobility of the trap, a scheme to further increase the hole concentration is obtained, which provides a new idea for p-type group-III nitride doping [285]. Although modulation doping brings us many surprises, the challenge of low conductivity caused by its discontinuous high resistance region is still worth noting. To solve this problem, it is easy to think of doping the entire superlattice. In this direction, Ebata et al. reported superlattice doping with a hole concentration of 3.4 × 10^18^/cm^3^ [286]. Recently, Wang et al. proposed a superlattice doping method for the ultra-thin epitaxial layer desorption-tailoring strategy. This phenomenon can give a good balance to the carrier concentration and transport. The effective activation energy of Mg can be reduced to reach a hole concentration as high as 8.1 × 10^18^/cm^3^ [287]. (Figure 21 shows the relevant experimental results).

#### 3.4.2. Delta Doping

**Figure 22:**
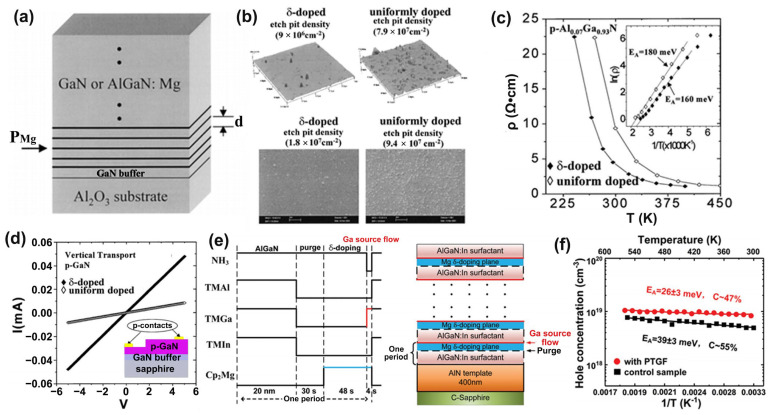
(**a**) Schematic diagram of Mg-delta-doped GaN or AlGaN, where *d* (=15 nm) and *P*_Mg_ denote, respectively, the distance between two delta-planes and the two-dimensional Mg doping concentration. (**b**) AFM and SEM morphologies of etched surfaces of p-type AlGaN epilayers after a 0.5 μm removal by inductively coupled plasma (ICP) etching. (**c**) The resistivity of representative uniformly Mg-doped and Mg-delta-doped p-AlGaN epilayers as functions of temperature. The inset shows the Arrhenius plots of the resistivity, which indicate that delta-doping reduces the activation energy of Mg acceptors in AlGaN. (**d**) Comparison of “quasi” vertical transport properties of uniformly Mg-doped and Mg-delta-doped p-type GaN. Etching depth (0.5 μm) and p-type ohmic-contact geometry were nominally identical for the two samples, as accomplished by ICP etching and photolithography patterning. Reproduced with permission [288]. Copyright 2003, AIP Publishing. (**e**) Flow sequence of the modified Mg-d doping process with indium as a surfactant (**left**) and the schematic side view of the epitaxial structure of the p-AlGaN grown with the pulsed TMGa flow (**right**). (**f**) Temperature-dependent hole concentration for the pulsed TMGa flow and control samples. The fitting curves are shown as solid lines. Reproduced with permission [295]. Copyright 2020, RSC Publishing.

The so-called delta doping means that the doping concentration in the material is distributed as a delta function. Nakarmi and Kim et al. introduced the Mg source during the switching off of the Al source and Ga source to distribute the concentration of impurity atoms formed into a delta function distribution. As a result, the longitudinal and transverse conductivity of the device was enhanced by two and three times, respectively (Figure 22a–d show the relevant experimental results). In addition, the material quality of the AlGaN epitaxial layer was improved, and its defect density was reduced [288,289]. The decrease in dislocation density can be explained as delta doping can inhibit the formation of pyramidal defects caused by the migration of excess Mg atoms to doping-induced GaNMgNGa stacking faults in the rapid annealing average doping technology [290,291,292]. Subsequently, Wang et al. obtained the same results by using delta doping to prepare p-GaN [293]. An indium-surfactant-assisted method has been introduced on the basis of conventional delta doping, and the hole concentration has been improved by more than three times [294]. Around a method of indium-surfactant-assisted delta doping, Qiu et al. have modified the Ga source into pulse mode and proved that the hole concentration had been improved nearly twice to 8.3 × 10^18^/cm^3^ [295] (the growth diagram and results are shown in Figure 22e,f). In addition, the application of delta Mg doping with the superlattice modulation doping can also be used to reduce the impact of the drawbacks of its discontinuous high-resistance region and improve its conductivity. Fan et al. have proved this method’s feasibility and applied the indium-surfactant method to obtain semiconductor superlattice structures with smooth surfaces, which have higher hole mobility [296]. In order to further understand the mechanism of this method, Zhao et al. carried out relevant research, whose realization method and mechanism are to increase the average hole concentration of the superlattice by delta doping at the superlattice interface, where the strong electric field generated by the energy band of periodic violent oscillation increases the ionization rate of Mg. They also used APSYS software to perform simulations, and the results showed that the LED had low voltage, high optical output power, and wall plug efficiency characteristics [297].

#### 3.4.3. Polarization-Induced Doping

**Figure 23:**
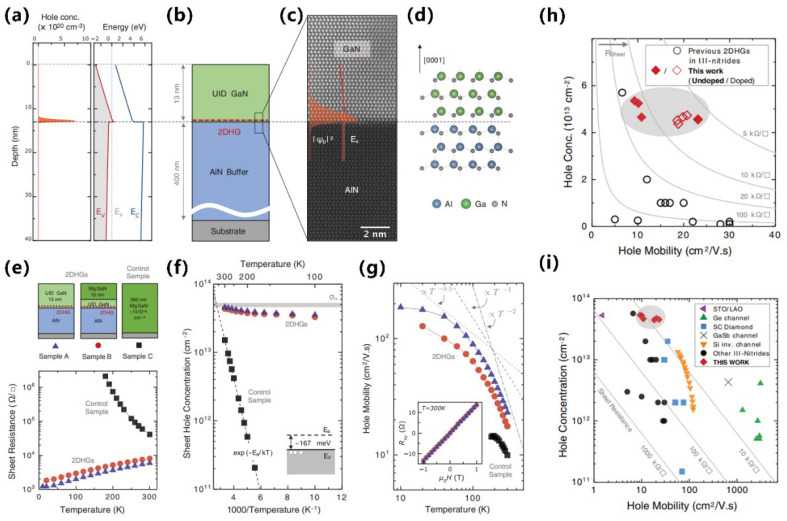
(**a**) The energy-band diagram of a 13 nm undoped GaN on AlN heterostructure shows the formation of a quantum well in the valence band and the high-density confined holes accumulated at the GaN/AlN interface. (**b**) Schematic of the epitaxially grown layer structure. (**c**) High-resolution STEM image showing the metal-polar wurtzite crystalline lattice of the heterointerface. The valence band edge and probability density of the holes from (A) are overlaid on the interface. (**d**) Schematic of the metal-polar GaN/AlN heterointerface, corresponding to the STEM image in (**c**). (**e**) The 2DHG samples A and B exhibit a metallic behavior of decreasing sheet resistance with decreasing temperature, whereas the control sample C is insulating in behavior, becoming too resistive below 180 K for measurement. (**f**) The measured mobile hole concentrations over a range of temperatures in samples A, B, and C. In the Mg-doped GaN (sample C), holes freeze out below 180 K. The density in the 2DHG of samples A and B shows almost no change in the hole concentration down to cryogenic temperatures. (**g**) The measured hole mobilities in samples A, B, and C for a range of temperatures. (**h**) Comparison with previously reported 2DHGs in nitride heterostructures (open symbols indicate Mg-typed doping). The doped, as well as undoped structures reported in this work, have much higher hole densities and decent mobilities, enabling record high p-type conductivity of 6 kilohms/square. (**i**) Comparing across other semiconductor material systems, such as oxides SrTiO_3_/LaAlO_3_, surface conducting diamond, Ge channels, Si inversion channels, and GaSb channels, this work has the highest room-temperature hole density and the highest conductivities among wide-bandgap semiconductors (III-nitrides, oxides, and diamond), the latter of which is critical for high-performing lateral power devices. The 2DHG in samples A and B show higher mobilities than that of C. (Inset) Hall resistance versus magnetic field measured at room temperature indicates a positive Hall coefficient (holes) in both samples A and B. Reproduced with permission [300]. Copyright 2019, AAAS Publishing.

Due to the need for the application, a solution to the low-temperature freezing and ionized impurity scattering phenomena in impurity doping is urgently needed to improve the hole mobility and bulk conductivity in p-type materials induced by impurity doping. A polarization-induced doping method based on the gradient layer of Al components was proposed by Simon et al., who successfully solved the above problems. They explained the polarization-induced doping mechanism as follows: with the increase in the Al component in the Al_x_Ga_1−x_N layer, the unit dipole strength linearly increases with the appearance of an unbalanced net polarization charge that produces a built-in electric field that ionizes the deep Mg atoms to form a high-density hole gas; in addition, the smooth valence band edges further facilitate the spatial transport of electron gas [298]. Following the above ideas, Yan et al. successfully used MOCVD to accurately control the thickness and composition of the N-polar Al_x_Ga_1−x_N layer and obtained effective polarization-induced doping of blue-purple LED with a gradual Al component epitaxial layer to also regulate the stress, which greatly enhanced the electrogenic spectral density [299]. In recent years, Chaudhuri et al. proposed and implemented a pioneering idea by growing non-doped GaN quantum wells on the AlN layer to further unlock the potential of polarization-induced doping in group-III nitride devices and achieve the required two-dimensional hole gas (2DHG) concentration without doping Mg [300] (Figure 23 shows the relevant experimental results). It provides a scheme for the application of non-Mg-doped polarization induction in LEDs. The research on polarization-induced doping is making progress. Huang et al. applied polarization-induced doping to a green LED and achieved an increase of nearly one order of magnitude in hole concentration [301]. Gu et al. proposed a strain-compensated B_0.14_Al_0.86_N/Al_0.5_Ga_0.5_N polarization-induced superlattice doping idea. The enhanced hole injection and reduced electron leakage were verified by simulation, and the hole concentration was 8 × 10^18^/cm^3^ [302].

#### 3.4.4. Future-Oriented Doping

The emergence of new things is bound to bring about breakthroughs and updates in related technologies. Will the emergence of nanonitriding LEDs, such as nanowires, bring us surprises in P-type doping? In order to answer this question, researchers conducted related studies on p-type doping. Nguyen et al. carried out p-type modulation doping of the InGaN/GaN QD structure on self-growing vertical nanowires without a catalyst based on a Si substrate, where the GaN layer is doped. It realized and confirmed the enhancement of the hole transport mechanism and the high luminous efficiency of phosphor-free white LEDs [303]. Zhao et al. demonstrated hole concentrations up to 1.3 × 10^19^/cm^3^ in AlGaN nanowires using measurements of photoelectrochemical techniques [304]. Crucially, it does not use extra-special doping methods, which leaves the imagination open to what the nanowire’s hole concentration limit is. It is believed that the efficient p-type doping mode facing the future will be dominated by nanowire and QD structures. Finally, Table 4 summarizes the p-type doping data from this chapter.

## 4. Micro-LED

The LED material structure is obtained by epitaxial growth through photomask deposition and insulation deposition (such as the use of plasma-enhanced chemical vapor deposition method to form SiO_2_ layer), lithography, etching (wet etching, inductively coupled plasma etching, reactive ion etching), insulation deposition, lift-off process, electrode fabrication (by sputtering, thermal evaporation, or electron beam evaporation), annealing (ohmic contact is formed between the p-type material and the electrode), and other optoelectronic processing processes such as indium tin oxide (ITO) layer manufacturing (if necessary), has completed the transformation from millimeters to micrometers with a size of 100 µm to 1 µm, in which each luminescent unit can be driven separately. Finally, through substrate stripping (laser stripping or chemical stripping) and metal interconnection, the micro-LED array and the driver circuit are integrated into one to realize the application. The changes brought by the size reduction are not only reflected in the process parameters and methods but also in the performance and application. The former is attributed to the size effect of micro-LEDs, including the influence of size change on electrical and photoelectric response characteristics. The innovation in the application is mainly reflected in display technology, light communication, and other applications, such as biomedical applications. These will be introduced in this chapter.

### 4.1. The Size Effect of Micro-LED

**Figure 24:**
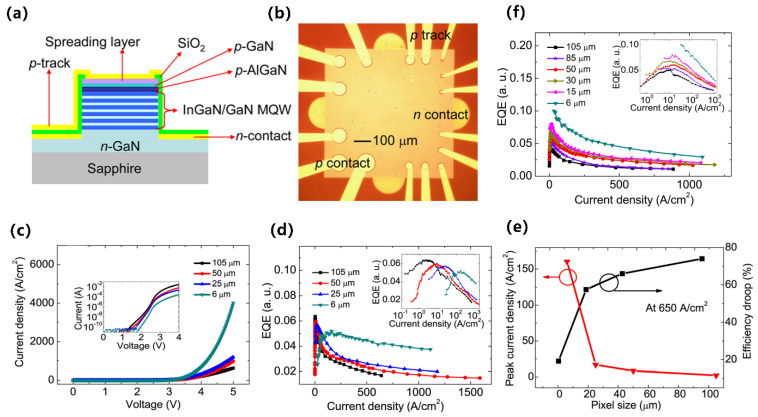
(**a**) Schematic structure and (**b**) optical micrograph of the micro-LEDs with diameters from 6 μm to 105 μm. Size-dependent characteristics of (**c**) current injection density versus voltage (inset: log (current) versus voltage) and (**d**) EQEs versus injection current density (inset: EQEs versus log(J)) for sample A. The thermal annealing time of sample A is 2 min. (**e**) EQE peak current density and efficiency droop at 650 A/cm^2^ as a function of pixel size. (**f**) Size-dependent characteristics of EQEs versus injection current density (inset: EQEs versus log(J)) for sample B. The thermal annealing time of sample B is 3 min. Reproduced with permission [216]. Copyright 2012, AIP Publishing.

**Figure 25:**
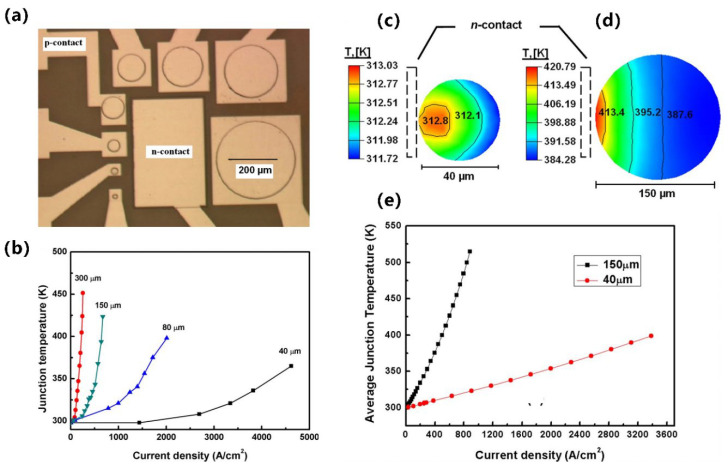
(**a**) (Color online) Optical micrograph of the fabricated LEDs with dimensions ranging from 10 to 300 μm. (**b**) (Color online) Measured junction temperature as a function of the current density for different pixel sizes. (Color online) Simulated junction-temperature distribution in the active area of (**c**) the 40 μm pixel and (**d**) 150 μm pixel under the same current density of 500 A/cm^2^ [picture sizes are not scaled; in the simulation, the n-contact is fixed at the left side, as indicated by the dashed rectangles in (**a**,**b**)]. (**e**) Simulated average junction temperature against current density for two different pixels. Note that in (**c**,**d**), the absolute temperature variation across the device is 28 times larger for the larger device. Reproduced with permission [305]. Copyright 2011, AIP Publishing.

Compared with LED, the micro-LED current density increases with the decrease in size, and the ratio of center current density to the edge (local) current density increases; that is, micro-LED with a small size has a high and uniform current density [216]. (Figure 24 shows the relevant experimental results). In addition, with the decrease in size, the self-heating effect is weakened, and the junction temperature rises more slowly [305]. (Figure 25 shows the relevant experimental results). Firstly, this can be attributed to the increase in the ratio of the sidewall surface area to the active area with the decrease in size, which contributes to heat loss. Secondly, the more uniform current distribution reduces local (near the n electrode) unnecessary heat production. Another way of thinking about it is that this also suggests that smaller micro-LEDs can maintain higher current densities. Related studies also show that the optical power density of micro-LEDs increases with the reduction in size, which can be attributed to the reduction in thermal power generated by the self-heating effect, as well as the shorter optical path, reduced light loss, and higher optical extraction efficiency with the smaller size. It is positive that the efficiency droop phenomenon is alleviated in micro-LEDs compared with large-size LEDs, and the smaller the size, the slighter the decrease [216] (Figure 24 shows the relevant experimental results). This is due to the uniform current distribution, which makes the edge electron leak and the local non-radiative recombination probability decrease. However, the maximum quantum efficiency of micro-LEDs with a smaller size is lower, which is interpreted to mean that micro-LEDs with a smaller size are more affected by surface non-radiative recombination due to sidewall defects [306,307]. Efforts in sidewall defect suppression, sidewall passivation by atomic layer deposition, sidewall passivation by hydrogen plasma, and sidewall passivation by combining tetramethyl ammonium hydroxide with SiO_2_ have been developed, all of which have significantly improved the EQE of micro-LEDs [308,309,310].

### 4.2. Quantum Efficiency

#### 4.2.1. Efficiency Droop

Efficiency droop is a phenomenon in which an LED’s (internal) quantum efficiency decreases as the injected current density increases. The physical mechanism of LED efficiency droop mainly stems from non-radiative compound losses and charge leakage. The IQE formula can be expressed by means of the *ABC + f*(*n*) model, as shown in Equation (5), [311]:

(5) $$IQE=Bn^{2}/(An+Bn^{2}+Cn^{3}+f(n))$$

where n represents the carrier concentration, and *A*, *B*, and *C* represent the Shockley–Read–Hall (SRH), radiative, and Auger coefficient, respectively. An and $Cn^{3}$ represent the Shockley–Read–Hall and Auger contributions in non-radiative recombination, $Bn^{2}$ represents radiative recombination, and f(n) represents carrier leakage out of the active region. Furthermore, an alternative explanation for the existence of other mechanisms can be found in the work of David, Fu, and Ryu et al. [312,313,314].

**Figure 26:**
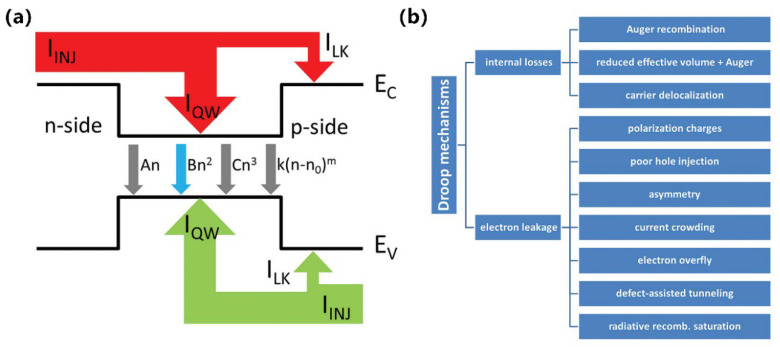
(**a**) Sketch of recombination and leakage terms in a single-QW or double-heterostructure LED. (**b**) Classification of droop mechanisms. Reproduced with permission [315]. Copyright 2013, AIP Publishing.

Focusing on the physical causes, as shown in Figure 26, Verzellesi et al. summarize some remedies [315]. In view of the reasons, some remedial measures are summarized and explained in combination with the research report. From Equation (1), we can see that the most intuitive way to reduce the contribution of non-radiative recombination, especially Auger recombination, to alleviate the efficiency droop is to reduce the carrier density in the quantum well. Simple measures can be thought of as thick QWs, increasing the number of QWs and making the chip area larger. However, this operation on the polar plane will reduce the quality of the material, which is not worth the loss. If epitaxial material quality can be further improved, perhaps it is a desirable idea to grow device structures on semi-polar or even non-polar surfaces to alleviate droop efficiency [316]. In fact, in terms of ease of operation, effective electronic leakage measures will be more cost-effective than reducing nonradiative compounding effects.

Some of the techniques and structures that have been mentioned in the previous section for minimizing electronic leakage to mitigate efficiency droop will be systematically reviewed, and new approaches will be introduced. Reducing material defects and strain induces polarizing charges that promote electron de-leakage to the p-doping area, which is always fundamental to reducing electron leakage, and the solutions mentioned above, such as diagonal substrates, graphic substrates, buffer layers, and superlattice structure are all designed to solve this problem (defect reduction is a fundamental principle not only for electronic leakage but also for non-radiative recombination). Additionally, p-type doped AlGaN EBL containing Al components is considered to be an effective structure to prevent electron leakage. On this basis, to realize the hole injection reinforcement to improve the condition of unbalanced electron–hole in an active area to reduce electron leak, gradient component EBL, superlattice EBL, and the superlattice structure EBL with gradient components were put forward (References [91,92] for details). What is more, the AlGaN/InAlN heterojunction with the ultra-thin InAlN insertion layer is also a new starting point for EBL [317]. The modification of the quantum barrier makes it better able to limit the hole electrons in the quantum well to reduce the overflow and enhance the hole injection. Due to the demand, the component gradient quantum barrier and the doping quantum barrier came into being [318,319]. The transverse current is also an important source of electron leakage. In addition, reducing the local current can also reduce the contribution of unnecessary local Auger recombination to the efficiency droop. In this regard, the application of micro-LED has been proven to reduce the local current and improve the current uniformity, as detailed in Section 4.1. Not only that, as discussed in the previous section, the nanowire LED can be considered in many ways so that the efficiency droop is inhibited (refer to [263,320] and Section 3.3.1 for details). And the relevant methods for the visible and ultraviolet light LEDs in Section 3.2.2 should not be forgotten.

#### 4.2.2. Light Extraction Efficiency (LEE)

As an important index to measure the performance of LEDs, EQE is defined as the ratio of the number of photons produced by radiation recombination and the number of electron holes injected per unit of time. From another perspective, EQE can be expressed as a formula:

(6) $$EQE=IQE\times LEE\times \eta_{ING}$$

**Figure 27:**
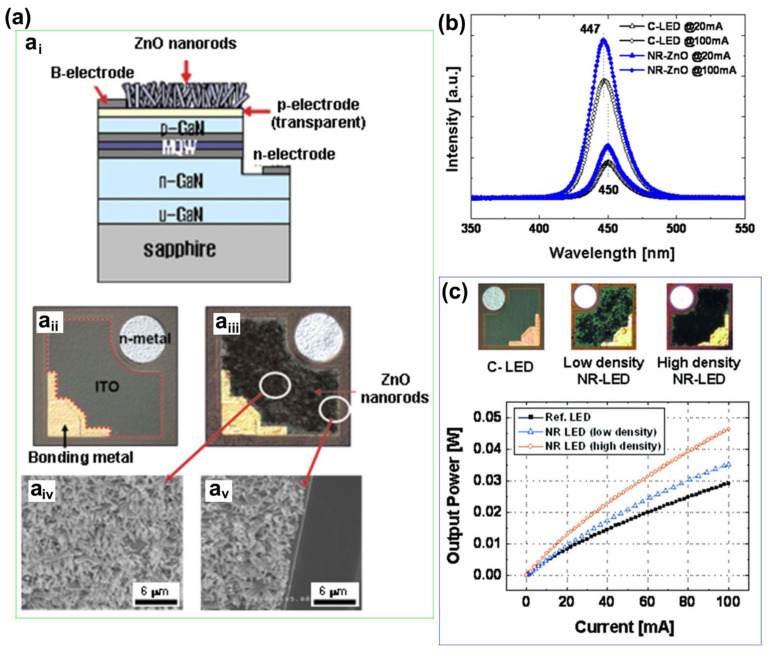
(**a**) Schematic diagram of GaN LED structure with ZnO nanorod arrays (**a_i_**). Top-view micrograph image of LEDs with conventional planar ITO (C-LED) (**a_ii_**) and top-view micrograph image of LED with ZnO nanorod array/ITO (NR-LED) (**a_iii_**). FE-SEM images of ZnO nanorods on the center (**a_iv_**) and the edge (**a_v_**) of planar ITO of NR-LED. (**b**) EL spectra of C-LED and NR-LED at 20 and 100 mA. (**c**) Micrograph images of C-LED, low-density NR-LED, and high-density NR-LED. The light output power of individual LEDs as a function of injection currents. Reproduced with permission [322]. Copyright 2009, AIP Publishing.

$\eta_{ING}$ is the carrier injection efficiency, which is the ratio of the number of electrons injected into the LED to the number of electrons provided by the power supply. Therefore, EQE is strongly related to IQE and LEE. IQE has been analyzed in the previous section, and this chapter will focus on how to improve LEE. Due to the absorption effect of the device material, the shielding of the electrode, and the Fresnel reflection or even total reflection between the two media with different refractive indices, some photons generated in the source region cannot escape successfully, which affects the efficiency of light extraction. Examples of ways to enhance LEE are presented here, including patterned substrates, surface roughness, and smaller-scale periodic micro-nano processes. As mentioned above, selecting substrate materials with an appropriate refractive index and high transmittance will improve the LEE of LEDs or the use of etching and other technological means to make the patterned substrate. Furthermore, other methods will be introduced here. Similar to the principle that improved the efficiency of light extraction by the patterned sapphire substrate, a rough or patterned surface is of great help in improving the efficiency of light extraction. Fujii et al. reported a rough surface LED with a micro-pyramid distributed on the n-type GaN layer with the sapphire substrate removed by laser stripping and etching, which improved the surface LEE by two to three times with a simple process [321]. Kim et al. prepared ZnO nanorods on a smooth ITO surface, and the results showed that LED light output efficiency with a rough surface was improved by 57% [322] (Figure 27 shows the relevant experimental results). As discussed above, V-pits are also effective for improving LEE. In this regard, Koike et al. studied a combination of LED structures that could not only improve the efficiency of light extraction but also give consideration to low leakage [323]. Finally, the LED with a rough surface distribution of V-shaped pits won out. Song et al. used the dry etching process of nanosilver to create an antireflective subwavelength rough surface structure on the ITO that reduces Fresnel reflection, which improves the LEE and increases the optical output power by 30.2% [324]. In addition to the above two methods, smaller-scale periodic micro-nano processes, such as manufacturing microlens (micro-dome) arrays, photonic crystal, micropore arrays on LED, and nanowire structure LED, have a positive effect on enhancing LEE. Ee et al. described a method for increasing LEE by fabricating SiO_2_/polystyrene concave microlens arrays with effective photon escape cones [325]. The optimized microlens parameters and LEE of different luminescence wavelengths were calculated by Monte Carlo ray tracing simulation. Finally, the real LEE improvement was consistent with the simulation. Zhao et al. studied the effect of a thin-film flip-chip LED with a micro-dome surface on improving LEE and increasing the effective photon escape cone [326]. FTDT calculation shows that for LED structures with high reflectance, adjusting the thickness of the p-type GaN layer can minimize the interference effect of reflected waves, and combining this with optimizing the diameter and height of the micro-dome can maximize the LEE. By optimizing the calculation to reduce the influence of Fresnel reflection further, the LEE at 460 nm and 550 nm peak wavelengths is more than 2.5 times that of a traditional inverted LED. In addition to the above two methods, applying a photonic crystal layer to an LED can effectively couple and extract the guided modes to reduce total reflection and improve LEE. Wierer Jr et al. transformed n-type GaN into a photonic crystal by the dry etching method and proved the superior optical control mode of the photonic crystal [327]. The best unpackaged LED LEE obtained was 73%, which broke the record at that time. Lin used ICP dry etching and chemical wet etching to manufacture LEDs with both a microporous array and textured sidewalls, which significantly increased the probability of photon scattering and effectively suppressed the total reflection phenomenon [328]. As previously described, the nanowire structure has a larger effective luminescence area. In addition, optimizing the parameters of the nanowire array to the couple and extracting its guided modes will effectively further improve LEE. Yue et al. simulated the parameters of a nanowire array by FDTD; optimized the height, diameter, shape, and spacing of nanowires; and summarized the relationship between these parameters and LEE [329]. Terazawa et al. successfully simulated the LEE of a core–shell nanowire LED by using FDTD, the rigorous coupled wave analysis method, and the ray tracing method, indicating that the N-type GaN layer can be used to bury the nanowire structure instead of ITO to further exploit the advantage of high LEE of nanowires [330].

Due to their small size, micro-LEDs enable the photons emitted from the source area to escape to the outside as soon as possible, thus reducing the light loss in the device to improve LEE, and that is one reason why micro-LEDs are becoming more popular. There is no difference between large-size LEDs and micro-LEDs in nature, only the size difference, so the above used to raise LEE also applies to micro-LEDs. Ryu et al. used the FDTD method to study the LEE of micro-LEDs with different shapes and sizes, and the results showed that the LEE of the square mesa is larger than that of the circle one at the same size, and the LEE decreases as the size of the micro-LEDs increases under the same shape [331]. Essentially, the only change here is the area proportion of the sidewall, which also confirms the importance of the sidewall light for LEE. Due to the size reduction, the sidewall cannot be ignored as an effective photon escape area, and the increase in effective escape area means another way to improve LEE, so optimizing the sidewall of micro-LEDs is essential. Current methods for improving sidewall LEE can be summarized as increasing the substrate thickness of inverted LEDs and preparing inclined sidewalls with air cavities [332,333,334]. It is predictable that the use of sidewall graphics for micro-LEDs will be highly anticipated in the future [328,335].

When the LED works, it will emit transverse-electric (TE) polarized light whose propagation direction is mainly parallel to the c-axis and transverse-magnetic (TM) polarized light whose propagation direction is primarily perpendicular to the c-axis. For InGaN-based visible or infrared LEDs, TE-polarized light is the leading light, while for AlGaN-based UV LEDs, the proportion of TM-polarized light increases with the increase in the Al component [336,337]. This means that for the AlGaN-based UV LED growing along the c-axis, compared with the visible and infrared LED, its effective luminous area decreases, and the change in the incident angle of light emitted from the plane perpendicular to the c-axis will result in the occurrence of the total reflection phenomenon more seriously, which will further lead to the reduction in LEE. Therefore, it is necessary to separately explain the LEE improvement methods of AlGaN-based LED. Patterned substrates [338], surface roughness [339,340], and smaller-scale periodic micro-nano processes [341,342,343,344,345] are also applicable to AlGaN-based LEDs, and the examples collected will be listed sequentially in the references, which are available for self-review and will not be repeated here. In particular, UV light absorption exists in the electrode and GaN layer of AlGaN-based LEDs, so additional measures must be taken to eliminate this effect. At present, electrodes or reflective layers with Al as the core that give consideration to good ohmic contacts and high-UV reflection, such as the V/Al, Cr/Al, and ITO/Al combinations, have been developed [346,347,348,349]. Alternative Ni/Au low-UV absorption electrodes, such as graphene, have also been discovered, and the application of these to eliminate the effect of electrode absorption of UV light has proved effective [350]. Schemes to replace p-GaN with highly transparent p-type AlGaN or M-doped short-period gradual SLs have also been validated to prevent the GaN layer from absorbing UV light [351,352]. Takano et al. used PPS, Rh electrodes, and resin coatings to enhance LEE and finally achieved a peak EQE of 20.3% at 275 nm [353]. Pandey et al. achieved 11% EQE at 265 nm by optimizing the material structure of the device without extra process improvement regarding LEE. This opens up the possibility of further enhancing EQE by enhancing LEE [354].

#### 4.2.3. The Effect of Temperature on Quantum Efficiency

**Figure 28:**
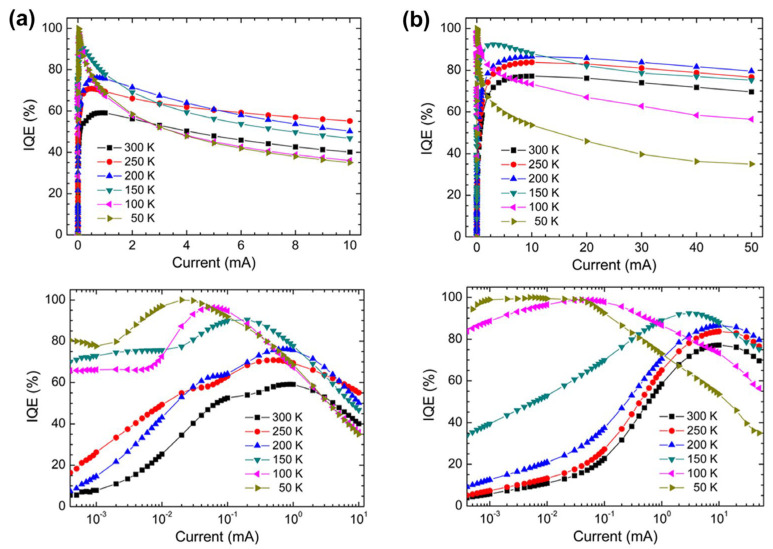
(**a**) Estimated IQEs for the blue LED plotted in linear (**top**) and log (**bottom**) scales for current. (**b**) Estimated IQEs for the green LED plotted in linear (**top**) and log (**bottom**) scales for current. Reproduced with permission [356]. Copyright 2012, AIP Publishing.

For LEDs, Shin et al. studied the variation of the IQE of LEDs with the current at different temperatures [355]. Figure 28 demonstrates the IQE as a function of current and temperature. The temperature range was from 50 K to 300 K, the current varied from about 10^−3^ mA to 10^2^ mA, and six control experiments were set up at 50 K intervals. In general, the changing trend of IQE first increases and then decreases. With the increase in temperature, the increasing trend of IQE becomes apparent, the maximum IQE decreases slightly, and the droop phenomenon of LED efficiency is evident at low temperatures (below 200 K). The obvious increasing trend can be attributed to the fact that, with the increase in temperature, the number of ionized holes increases, and more electrons need to be injected to meet the saturation of electron–hole recombination. The strong efficiency droop phenomenon at low temperatures can be explained by the electron leakage caused by the low number of holes injected for radiative recombination. The reduction in the maximum IQE can be explained by the increase in nonradiative recombination and electron leakage caused by the increase in carrier concentration at low temperatures (although the radiative recombination coefficient is reduced, the carrier concentration is increased, so it is not certain whether the radiative recombination is weakened and whether it is the reason for the decrease in IQE [356]). The experiments show that the variation of EQE related to temperature is similar to that of IQE.

From the essence, it is not difficult to conclude that the influence of temperature on LEDs and micro-LEDs is connected. Tian et al. studied the EQE of micro-LEDs at temperatures ranging from 300 K to 500 K as the injected current density increased [357]. Similar to the changing trend of LEDs mentioned in the previous paragraph, the EQE of micro-LEDs increases first and then decreases with the gradual increase in current density. It shows more details of the efficiency droop phenomenon at high temperatures than the Shin et al. experiment. At high temperatures, with the increase in temperature and injection current density, the carrier concentration in both cases shows an increasing state. This means that in the context of the electron–hole radiation recombination saturation, the higher the temperature, the greater the electron leakage. In addition, under high temperatures, it is found in the study of each recombination coefficient that, with the increase in temperature, the SRH recombination coefficient increases and the radiation recombination coefficient decreases, which is consistent with the law of LEDs at low temperatures (The SRH coefficient increases with increasing temperature at low temperatures). However, different from the low-temperature case, the Auger recombination coefficient decreases with increasing temperature at high temperatures (the Auger recombination coefficient increases with increasing temperature at low temperatures [358]). In summary, with the increase in temperature, we can be sure that the decrease in EQE at maximum saturation recombination results from the enhancement of electron leakage and SRH recombination (the effects of Auger and radiation recombination should be further investigated). It can be seen that temperature greatly impacts LEDs’ performance, and an understanding of how to effectively dissipate heat to make LEDs work at a constant and appropriate temperature is crucial. Please refer to Liu et al.’s review article for detailed research progress on LED heat dissipation [359].

### 4.3. Spectral Characteristics

According to semiconductor physics knowledge, theoretically, LED peak luminescence wavelength meets the following equation:

(7) $$\lambda =1240/E_{g}(eV)(nm)$$

λ is the peak luminescence wavelength in nanometers, and $E_{g}$ is the bandgap width of semiconductor materials in eV. However, in practice, the luminescence wavelength will sometimes deviate from its theoretical value with the change in the injection current. This phenomenon occurs because LEDs are affected by QCSE, band filling, and thermal effects [360,361]. The so-called QCSE refers to the phenomenon of a blue shift of the peak wavelength of LED luminescence with the increase in injection current, and its mechanism is introduced in Section 3.2.2. The band-filling effect refers to the fact that, with the rise in the injected current density, and when the number of carrier recombination is less than the number of carrier injections, the low energy level is filled first so that the carriers can only transition to a higher energy level before recombination, which is equivalent to the widening of the band gap, finally leading to the blueshift of the peak emission wavelength of LEDs. The thermal effect refers to the phenomenon that with the increase in the injection current, LED self-heating causes the junction temperature to rise, which leads to the narrowing of the band gap width and, finally, to the redshift of the peak luminous wavelength. In detail, under the joint influence of the above three mechanisms, the spectral characteristics of large-size LED and micro-LED are slightly different. With the increase in injected current density, a large-size LED generally shows a redshift, while a micro-LED shows a blueshift first and then a redshift and a large-size LED has a stronger degree of redshift than a micro-LED. With the increase in injected current density, a large-size LED generally shows a redshift, while micro-LED shows a blue shift first and then a redshift and a large-size LED has a stronger degree of redshift than micro-LED.

### 4.4. The Application of Micro-LED

#### 4.4.1. Full-Color Display Technologies Based on Group-III Nitride Micro-LEDs

##### Color Conversion Technology Based on Group-III Nitride Materials

Taking LED devices down to the micron scale has revolutionized display technology. On the one hand, compared with traditional liquid crystal display (LCD) and organic light-emitting diode (OLED) display technologies, the advantages of the high pixels per inch of micro-LED make up for the shortcomings of conventional display technologies in virtual reality, augmented reality, and other fields that provide support for future metaverse implementation. On the other hand, micro-LEDs have high brightness, low power consumption, and lightweight features, making them more suitable for small display devices such as smartwatches and mobile phones. In addition, the high physical and chemical stability of nitriding micro-LEDs makes them more compatible with the application environment, such as the display of some special equipment in a high-temperature environment. However, group-III nitride micro-LED is not without challenges in the field of display technology. At present, it mainly exists in full-color realization. Relevant research has been carried out in the fields of color conversion of group-III nitride materials.

Most red LEDs on the market today are made of AlGaInP materials, but as their size is reduced, their efficiency will droop [362]. Its poor thermal stability and mechanical properties limit its application scenarios, and its compatibility is poor when used with blue and green InGaN LEDs. In this regard, the application of InGaN material can be completely solved, so the development of all-color LEDs based on InGaN color conversion material is very important. The so-called color conversion refers to the phosphor-free luminescence conversion from short-wave blue light to long-wave red light to realize full-color display and white light illumination based on InGaN LEDs. The main difficulties in achieving color conversion are due to the low quality of high-indium component materials, and the QCSE becomes stronger with the increase in indium components, which will seriously affect the photoelectric performance of LEDs. LEDs’ color conversion cannot be completely solved with a single structure or technology. Focusing on solving this problem is the current research hotspot of InGaN LEDs. The issue was touched upon somewhat in the previous review, and we will introduce it in detail in this section.

**Figure 29:**
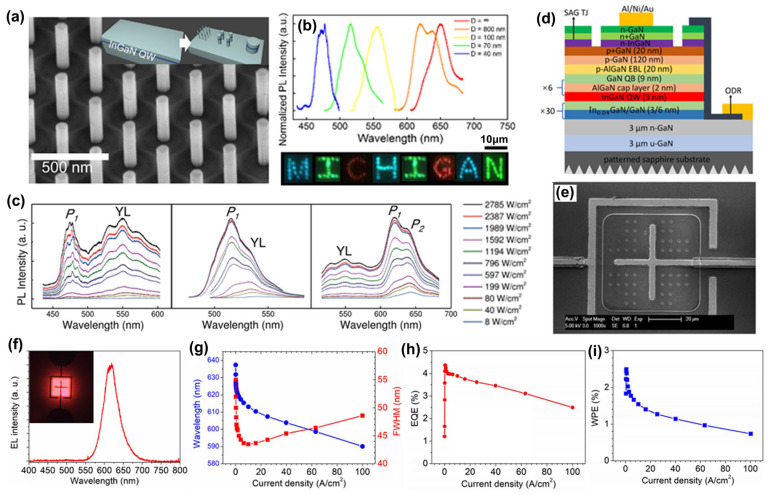
(**a**) The bird’s-eye view of the blue-emitting nanopillar structures consisting of a single InGaN quantum well. The inset shows the schematic of how different diameters of nanopillar structures were fabricated from a standard InGaN quantum well epitaxial wafer. (**b**) Normalized photoluminescence spectra of InGaN nanopillars of various diameters (D). The optical image “MICHIGAN” letters comprising nanopillars of three different diameters (40, 70, and 800 nm) were taken by a CCD camera. (**c**) The normalized photoluminescence spectra at different excitation intensities (shown in the legend) for three different nanopillar diameters: 40 (**left**), 70 (**middle**), and 800 nm (**right**). The peak P_1_ is attributed to the main emission from the quantum well. YL is attributed to the yellow band luminescence, and P_2_ is attributed to the emission from localized states. Reproduced with permission [363]. Copyright 2016, AIP Publishing. (**d**) Schematic epitaxial stacks and devices structure of InGaN red micro-LEDs. (**e**) SEM image of the 60 × 60 μm^2^ InGaN red micro-LEDs. (**f**) EL spectrum at 1 A/cm^2^. The inset shows the micro-LEDs EL image under a microscope. (**g**) Peak wavelength and FWHM vs. injection current density. (**h**) EQE and (**i**) WPE vs. current density of the InGaN red tunnel junction micro-LEDs measuring in an integrating sphere. Reproduced with permission [368]. Copyright 2022, AIP Publishing.

Through the above reading, I think we have our own ideas on how to improve the quality of materials, curb QCSE, and alleviate quantum localization in InGaN materials with high indium content. Is it substrate modification, quantum well modification, or nanowire structure? Or something entirely new? Do not worry if you do not have an idea; paths to progress based on group-III nitride material color conversion will herein be introduced. Progress has been made in this area, as described here. Teng et al. etched InGaN MQWs from top to bottom to form nanocolumns (nanowires) of different diameters with different indium components to cause strain differences. The wavelength tuning of 178 nm was achieved by adjusting the strain size, and the LED was induced to emit red light to achieve full color [363]. Figure 29a–c show their structure and luminescence. Meng et al. found indium-rich clusters like QDs in quantum wells by growing LEDs of InGaN/GaN MQWs without additional processing to achieve red luminescence [364]. Zhang et al. combined two orange quantum wells and seven yellow quantum wells into a multi-quantum well structure as the active region, in which V-shaped pits were used to shield dislocations to increase hole injection and reduce compressive strain. A series of LEDs with peak values from 594 nm to 621 nm have been successfully obtained, which is very close to achieving efficient red luminescence [365]. Following this idea, Wang et al. achieved an approximate full-color display by growing two quantum wells with different indium components, changing the injection current, and co-operating with pulse width modulation technology [366]. Recently, Li et al. reported a red-light micro-LED with a stress-regulated superlattice structure grown on a graphically sapphire substrate and contacted via an epitaxial tunnel junction, with a peak luminescence wavelength of 623 nm and an EQE of 4.5%, the maximum known to date [367]. Figure 29d–i show the relevant structure luminescence and efficiency.

**Figure 30:**
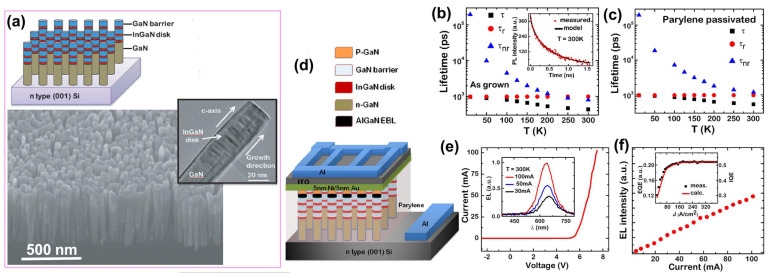
(**a**) Schematic and 45° tilted scanning electron microscopy image of InGaN/GaN disks-in-nanowires grown on (001) Si substrate. Inset to (**b**) shows a high-resolution transmission electron microscopy image of InGaN disks in a single GaN nanowire. (**b**) Variation of radiative, non-radiative, and total carrier lifetime with temperature in (**b**) as-grown, (**c**) parylene passivated disks-in-nanowires. The inset in (**b**) shows a typical TRPL transient of an as-grown sample measured at 300 K with 405 nm excitation. The solid curve is calculated with the stretched exponential model. (**d**) Characteristics of disk-in-nanowire LED on silicon with parylene passivation: (**d**) schematic representation; (**e**) measured current–voltage characteristics. Inset shows injection current-dependent electroluminescence spectra measured at room temperature; (**f**) light-current characteristics measured with pulsed bias. Inset shows the corresponding variation of EQE with injection current density. Reproduced with permission [367,369]. Copyright 2013, AIP Publishing.

**Figure 31:**
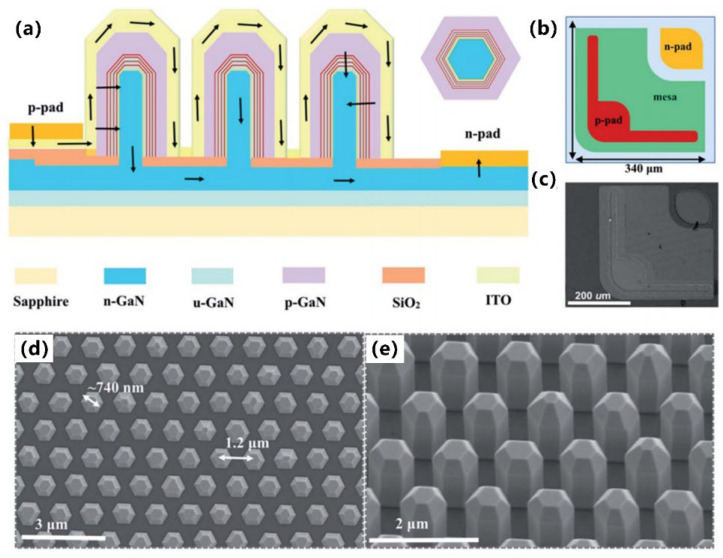
(**a**) Schematic diagram of the nanowire-based LED device structure. The hexagonal shape of the cross-sectional view of one nanowire is illustrated on the right, involving the n-type core, GaInN/GaN multiple-quantum-shell (MQS) active layer, and p-type GaN shell. (**b**) The designed LED chip (340 mm × 340 mm) and the corresponding (**c**) scanning electron microscopy (SEM) image after the process. (**d**) The planar-view SEM image of an as-grown nanowire sample, and (**e**) the 30°-tilted view SEM image. Reproduced with permission [374]. Copyright 2022, RSC Publishing.

**Figure 32:**
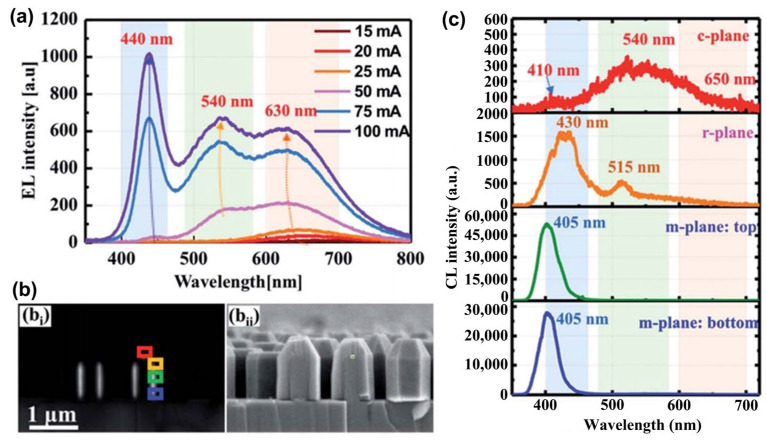
(**a**) Electroluminescence spectra of sample A at different injection currents. Three peaks located at 440, 540, and 630 nm are identified. (**b**) The cross-sectional view: CL panchromatic mapping (**b_i_**) and SEM images (**b_ii_**) of the nanowires in sample A. Panels in (**c**) show the CL spectra acquired in the c-plane apex region, r-plane, and top and bottom areas on the m-plane. Reproduced with permission [374]. Copyright 2022, RSC Publishing.

On the basis of InGaN, structures such as nanowire and QDs structures LEDs are also making breakthroughs. Jahangir et al. used plasma-assisted self-growth mode to grow GaN-based nanowires, in which InGaN/GaN nanodisk was the active region on the Si substrate, and surface nitriding was performed using either parylene or Si_3_N_4_ to reduce the surface state density. The 650 nm red emitting LED with an IQE of 52% and the 610 nm red emitting LED with an IQE of 55% under the condition of high current density injection were successively obtained [367,369] (Figure 30 shows the relevant experimental results). However, the EQE is lower under high current density injection. Zhao et al., based on the InGaN/GaN disks-in-nanowires structure, LEDs grow on the TiN/Ti/Mo metal layer, which significantly improves the EQE. In addition, research has proved that the metal layer can effectively dissipate heat and keep the junction temperature constant. The high EQE of 710 nm emitting droop-free red light with stable output becomes a reality [370]. Of course, the growth of nanowires on transparent amorphous layers also needs attention. However, the problem of how to grow large-scale, high-quality nanowires on transparent amorphous layers has been around for a long time. With the successful growth of GaN nanowires on SiO_x_ layers by Zhao et al., the solution to such issues has gradually become clear. It is found that compared with the Si substrate, the red luminescence of the nanowires grown on SiO_x_ is more efficient at 650 nm [371]. Recently, Pandey et al. performed in situ annealing in the active region of InGaN to suppress the defect, resulting in an increase in the luminescence intensity of more than one order of magnitude and an adjustable peak color wavelength from 550 nm to 650 nm with an appropriate increase in the diameter of the nanowires. Its EQE is 1.2% at 620 nm, which is the highest known EQE of nanowire-structure LEDs [372]. In addition to the vertically structured nanowire LEDs described above, core–shell structured nanowire LEDs have also been widely reported to be dedicated to red light emission in recent years. Lu et al. reported a method to increase the growth temperature of the barrier layer and insert the AlGaN spacer layer when growing in the active region of InGaN/GaN based on core–shell nanowire LED. The results show that this method can improve the luminescence intensity, indium incorporation, and crystallization quality, which promotes the transition of the emission peak to a long wavelength [373]. Subsequently, the red light-emitting LED based on core–shell nanowires were successfully reported by Ito et al. using a similar method, confirming this scheme’s feasibility [374]. Figure 31 and Figure 32 show their structure and luminescence. Red luminescence not only depends on increasing indium content but can also be achieved by injecting rare earth ions (such as Eu^3+^ and Pr^3+^) into the nanowire structure, as reported by Cardoso et al. and Lorenz et al. [375,376,377]. Additionally, as mentioned above, group-III nitride QDs based on planar or nanowire structures also have an essential component in color conversion, so we can refer to the previous article here. Nanowire LEDs were once considered to be the most competitive solution for high-efficiency and high-indium component red light emission, but their performance has not met expectations compared to planar structure LEDs. Although its IQE for red emissions has exceeded 50% in the past few years, its EQE has yet to break by 2%. This can be attributed to the fact that the physical mechanisms of nanowire group-III nitride structure and planar thin film structure may be different and less understood, resulting in the relevant experience of a planar structure not being ideally applied to a nanowire structure, which leads to the technical blind spot in the further improvement of IQE. In addition, the large non-radiative recombination caused by SRH, which is difficult to inhibit effectively, and inappropriate optical extraction methods are also reasons for the low EQE of nanowire-structured LEDs.

In addition to making the group-III nitride material itself glow red, strategies based on blue and purple InGaN LEDs to excite phosphors and hetero-colloidal QDs to glow red are currently mature solutions. However, for phosphors, the large size can cause low color conversion efficiency, and the small size can cause efficiency droop. For heterogeneous colloidal QDs, their instability will shorten the device life, limit the application, and even cause destruction by photons, which will further reduce their low quantum efficiency. Moreover, heterogeneous colloidal QDs require additional or even complex operations to integrate them into LEDs, and the gap between them and quantum wells will limit color conversion efficiency. Therefore, in summary, it is significant to develop red light LED/micro-LED based on group-III nitride material itself.

##### Mass Transfer and RGB Integration Technologies

For now, it is easier to assemble and transfer different-colored LEDs to the same substrate in a mass arrangement than to grow red, green, and blue (RGB) nitride LEDs on the same substrate simultaneously. And this process of transfer, arrangement, and assembly is called mass transfer. One of the challenges hindering the commercialization of low-cost micro-LEDs is that inefficient large-scale chip transfers cannot meet production demand, leading to higher costs. To this end, some mass transfer techniques have been developed. At present, mass transfer technology can be divided into pick-up transfer and self-assembly transfer. For information on how they work, current difficulties, and research progress, please refer to the review articles by Ryu et al. and Anwar et al. [378,379].

The so-called RGB integration is to connect the three-color LED chip array with the driving circuit after the massive transfer, then selectively control the luminescence of the LEDs through the input signals, and finally realize the pattern display. At present, RGB integration is mainly divided into two main research directions: one is the hybrid integration of silicon-based complementary metal oxide semiconductor (CMOS) readout circuits and LEDs, and the other is the integration of thin film transistors (TFT). The technologies related to the hybrid integration of group-III nitride chips and Si-based CMOS readout circuits to complete imaging and display have been developed as early as this century. Compared with silicon-based CMOS readout circuits, TFT-integrated RGB micro-LED arrays have advantages in terms of high drive current and cost-effectiveness, which are divided into Si-base TFT, indium gallium zinc oxide TFT, and MoS_2_ TFT from the material perspective [380]. In general, in optimizing RGB three-dimensional integrated manufacture, it is mainly reflected in the array manufacturing and bonding processes. Currently, based on standard optoelectronic machining processes, array manufacturing processes have traditionally relied on etching processes to manufacture array mesa, among which wet etching has been shown to have advantages in improving electron mobility [381]. The flip-weld bonding process is currently being used commercially. Among them, the photoelectric chips are welded through metals such as indium, tin, and gold, which are manufactured in the form of metal bumps on metal electrodes for electrical connection [382,383,384]. However, this is limited by the alignment accuracy. To solve this problem, monolithic hybrid integration processes, as well as COB processes, which rely on layers of conductive material for electrical connections, were developed [385,386]. In addition, no additional electrical interconnect process is required; using the etching processes and selective region growth, the scheme of fabricating LED chips on the same Si-based wafer with readout circuitry can eliminate the consideration of alignment accuracy, but it is clear that it can be affected by lattice mismatches [387]. To mitigate this effect, the Si_x_Ge_1−x_ gradient buffer layer scheme is proposed [388]. In recent years, with the in-depth research on two- dimensional materials and the development of related technologies, micro-LEDs have greatly promoted the development of the display field, especially for vertically stacked three-dimensional RGB integration, which is beneficial for significantly increasing pixel density and has great prospects [13].

#### 4.4.2. Light Communication

**Figure 33:**
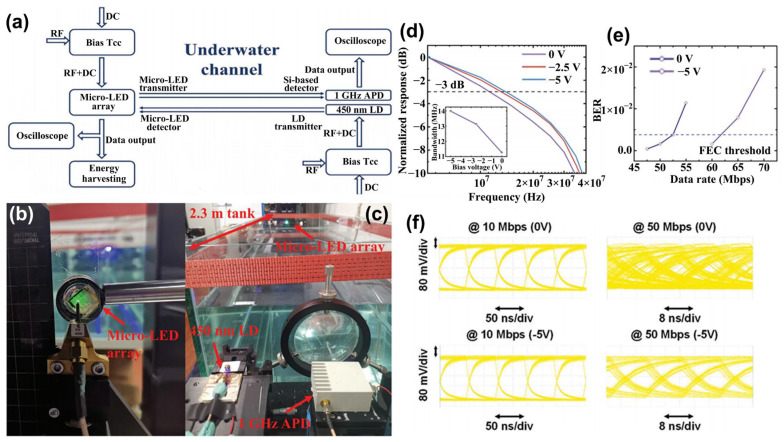
(**a**) Schematic diagram of duplex underwater wireless optical communication (UWOC) system based on a micro-LED array. (**b**) The photographs of the micro-LED collection as a transmitter and (**c**) a photodetector of the duplex UWOC system. (**d**) Frequency response curves of micro-LED-based photodetector under different bias conditions, inset: the extracted −3 dB modulation bandwidth of microLED photodetector. (**e**) Bit error rate (BER) versus data rate curves of micro-LED-based photodetector at different bias conditions in the 2.3 m duplex UWOC system. (**f**) Eye diagrams were captured at data rates of 10 and 50 Mbps under 0 and −5 V bias voltage, respectively. Reproduced with permission [404]. Copyright 2021, Wiley Publishing.

VLC can combine solid-state lighting (SSL) and optical communications, with advantages including high data rates, energy efficiency, enhanced security, and no radio frequency interference. VLC, which has been studied for decades, is a promising solution to the limited availability of the radio frequency spectrum since the visible light spectrum offers sufficient bandwidth that is unlicensed and free to use. LEDs are used for light communications in both free space and fiber-based systems due to their high efficiency. There are two main types of VLC: fiber-based and free-space. As for fiber-based VLC, plastic optical fiber (POF) is widely used as a transmission medium for in-building data networks. On the other hand, free-space VLC has attracted great attention for using white-emitting LEDs for both illumination and data transmission. The benefits of using white-emitting LEDs for VLC include the availability of unlicensed bandwidth, compatibility with existing radio-based transmitters, and the potential to implement wireless communications in environments where radio communications are not possible, like within aircraft. In addition, the blue-green light communication based on 400–550 nm has low attenuation underwater, which is considered to be the best solution to replace high-delay underwater acoustic communication and high-attenuation radio frequency communication in the future. In the invisible band, solar blind UV communication based on 200–280 nm has great application prospects, such as non-line-of-sight communication, because it has no natural UV interference. However, LED-based optical communications are limited by their modulation bandwidth and reduced efficiency, so micro-LED is expected to be very promising. It is well known that the shorter the carrier lifetime, the shorter the LED’s response time, ultimately resulting in increased modulation bandwidth. The carrier lifetime is strongly influenced by current density; the greater the current density, the shorter the carrier lifetime, and the greater the modulation bandwidth. Therefore, as the LED size decreases, the current density increases and the modulation bandwidth increases [389,390]. To take it a step further, the EQE decreases at a high injection current due to Auger recombination, electron leakage, and defect-assisted recombination, while the EQE of the micro-LEDs can keep up at high current densities and only with a small efficiency droop problem, which makes micro-LEDs offer higher bandwidth than large-size LEDs. In addition, compared with large-size LEDs, micro-LEDs have the following advantages in VLC: (1) The micro-LED is more cost-effective than the large-size LED. (2) The micro-LEDs may offer potential advantages of parallel data communication using multiple micro-LED pixels to further increase the data rate. As a consequence, micro-LEDs are the preferred option for light communication, with their high bandwidth and transmission rate, which is considered to be the efficient solution for high-speed communication as well as 6G. A typical report on a full-duplex underwater wireless optical communication (UWOC) system based on a micro-LED array (Figure 33) vividly demonstrates the application potential of micro-LEDs in the VLC field. Recent advances in light communication are summarized in Table 5. From the table, it can be seen that the modulation bandwidth of UV-oriented optical communication decreases obviously, which is due to the longer response time of UV LEDs. However, it is worth noting that Sun’s research team recently has succeeded in developing a single LED device that can be used to send and receive signals. It has a response speed of 3.7 ns in detection mode at 265 nm and an optical bandwidth of more than 585 MHz in emission mode; these results are all record-breaking [391]. Recently, they also designed a triangular micro-LED with high light extraction and light absorption, which is very novel for full-duplex wireless optical communications [392]. Device design that facilitates on-chip integration of a single chip capable of realizing transceiver signals seems to be the mainstream of the future; another example is the ring strategy proposed by Chen et al. [393]. Recently, Li et al. achieved dual-band UV luminescence by designing a special single-layer quantum well structure with stable spectral controllability, which is of great significance for solar-blind UV optical communication [394]. Yang et al. improved the LEE by etching the GaN absorption layer and adding a full-spatial omnidirectional reflector, resulting in a data rate of up to 3.819 Gbps in the solar-blind UV band [395]. Relevant studies have found that AlGaN UV QDs have very short response times up to the picosecond level, which may be a future direction [396].

**Table 5:** Recent advances in light communication.

Wavelength [nm]	Modulation Bandwidth [MHz]	Highest Transmission Rate [Gpbs]	Type	Single Pixel Size [µm]	Distance [m]	BER [10^−3^]	Modulation Format	Application	Reference
480	1000	4	Single pixel µLED	75	3	3.2	QPSK-OFDM	Free space	[397]
277	497.58	1.6	Single pixel µLED	40	1	3.4	16-QAM- OFDM	Free space	[398]
475	1530	5.27	Single pixel µLED	60	1	3.1	OFDM	Free space	[399]
400	1200	5.71	3 × 3 µLED array	20	13	3.7	DCO-OFDM	Free space	[400]
451	1200	4.86	3 × 3 µLED array	20	13	3.3	DCO-OFDM	Free space	[400]
509	1050	4.39	3 × 3 µLED array	20	13	3.7	DCO-OFDM	Free space	[400]
556	500	0.82	3 × 3 µLED array	20	13	3.3	DCO-OFDM	Free space	[400]
276	439.5	2.28	Single pixel µLED	100	0.25	3.0	OFDM	Free space	[401]
276.8	452.53	2	Single pixel µLED	100	0.5	2.86	16-QAM-OFDM	Free space	[402]
276.8	452.53	0.82	Single pixel µLED	100	3		16-QAM-OFDM	Free space	[402]
279	380	0.667	16 × 16 µLED array	25	0.7	20	OOK	Free space	[403]
279	380	0.557	16 × 16 µLED array	25	0.7	2	OOK	Free space	[403]
279	380	1.087	16 × 16 µLED array	25	0.7	13	16-QAM-OFDM	Free space	[403]
279	380	0.97	16 × 16 pixel array	25	0.7	2	16-QAM-OFDM	Free space	[403]
450	251.3	0.66	Single pixel µLED with LD	80	2.3	3.3	NRZ-OOK	Underwater	[404]
Green light emission	131	1.11	Single pixel µLED	80	3.4	<3.8	16-QAM-OFDM	Underwater	[405]
623/521/457/562/486	535/537.5/525/525/525	14.81	Five-primary-color LED	600	1.2	<3.8	64-QAM-DMT	Underwater	[406]
623/521/457/562/486	600/600/575/600/602.5	15.17	Five-primary-color LED	600	1.2	<3.8	Bit-loading-DMT	Underwater	[406]

#### 4.4.3. Charge Management Applications for Gravitational Wave Detection

In the spatial environment, UV LEDs can be used for charge management of laser space interference gravitational wave detection, reducing the charge accumulation on the inspection mass to reduce their additional Lorentz and Coulomb force interference [407]. Compared with large-size LEDs, micro-LED has great improvements in power density, current distribution, self-heating effect, modulation bandwidth, LEE, self-heating effect, and so on [408,409]. For UV LEDs, besides high-speed UV communication, especially for charge management of gravitational wave detection, it is of great significance. Non-contact charge management for gravitational wave detection in the spatial environment using high-efficiency ultraviolet micro-LEDs can reduce the influence of mechanical coupling on the sensor compared with contact charge management. In addition, an ultraviolet LED has the advantages of smaller size, lighter weight, lower power consumption, higher stability, and a longer lifespan than mercury lamps, as well as low electromagnetic interference, which means that UV LED applications can save power and further increase the laser power of interferometry to enhance sensitivity [410]. Therefore, the performance of AlGaN-based UV LEDs is very important, and UV LEDs have been extensively studied. However, the efficiency of UVB LEDs still needs to be improved, and the relevant parameters of its research progress are summarized in Table 6.

#### 4.4.4. Biomedicine

##### Public Health

**Figure 34:**
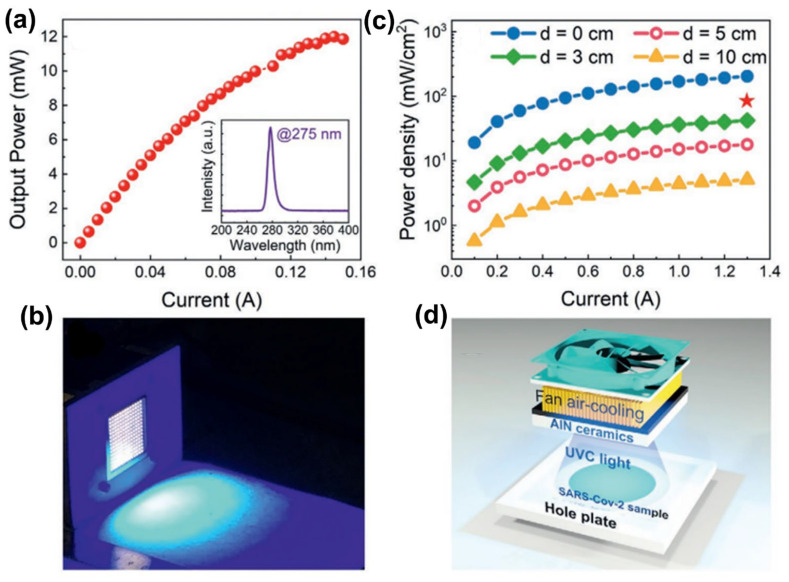
(**a**) Output power versus current curve of a single packaged UVC LED (the electroluminescence spectrum is given in the inset). (**b**) Demonstration of integrated sterilization light source fabricated by UVC-LEDs (without SARS-CoV-2 sample). This integrated array is composed of 13 parallel connected units, and each unit includes 15 UVC LEDs in a series connection. (**c**) Current dependent power density at different distances from the irradiation source. The output power density obeys an inverse-square law as a dependence of irradiation distance, suggesting the importance of choosing a suitable working distance. Considering the working efficiency of every single LED, a working current of 1.3 A is chosen for the integrated source, in which condition each device works at 100 mA. The output power density adjacent to the array (d = 0) is 192 mW cm^−2^ at 1.3 A. The working point in the virus-eliminating experiment is marked by a red star (94 mW cm^−2^). (**d**) The schematic image of the virus eliminating experiments. Reproduced with permission [416]. Copyright 2021, Wiley Publishing.

As is known to all, ultraviolet light can kill bacteria and viruses, and the application of ultraviolet micro-LEDs can better achieve this goal. Especially in the global epidemic of COVID-19, public health issues have become particularly important, and large-scale ultraviolet disinfection devices in public places are very necessary. High-power ultraviolet irradiation is needed to achieve efficient sterilization. To meet this desire, high-power micro-LEDs based on AlGaN material have been developed, which can kill all COVID-19 viruses in one second [416] (Figure 34 shows the relevant schematic and experimental results). Currently, the global epidemic has leveled off, but we should not take it lightly. No one knows what new viruses will emerge in the future. This requires continuous progress in the research of UV LEDs so as to gradually improve their quantum efficiency and output power.

##### Optogenetics

**Figure 35:**
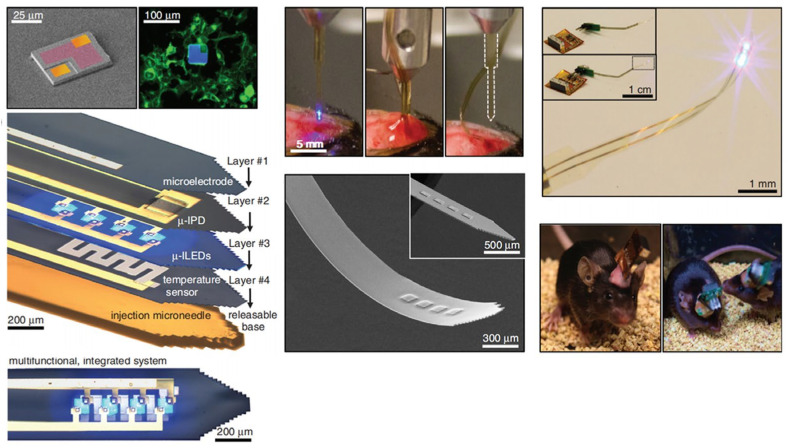
Injectable, cellular-scale semiconductor devices with multifunctional operation in stimulation, sensing, and actuation. Reproduced with permission [417]. Copyright 2013, AAAS Publishing.

Compared with large-size LEDs, micro-LEDs have the characteristics of fast response and high brightness, which makes them more suitable for photoelectric conversion. By developing small, flexible micro-LEDs based on flexible electronics to interact with biological tissues, the development of optogenetics has been greatly promoted [417] (Figure 35 shows the relevant schematic). The theory behind so-called optogenetics is that by applying fluorescent dye to the cell membrane, the electrical signals generated by nerve cells will be transformed into light signals of different colors, and then the information of neurons will be reflected through the detection of light signals of different colors. In addition, micro-LEDs implanted in the body can also stimulate and control nerve cells by releasing different colored light signals to convert electrical signals. So far, researchers have successfully used micro-LED to capture and study the neural circuits in mice, control the neurons, and even manipulate complex behaviors [418,419]. This suggests the possibility of treating some diseases by manipulating the nerves. Recent advances in optogenetics in primates, where researchers successfully implanted LED arrays into the brains of large primates, have enabled safe and repeatable optogenetic experiments with improved light transmission methods that lead to the development of brain implant devices [420].

##### Optoelectronic Tweezers

**Figure 36:**
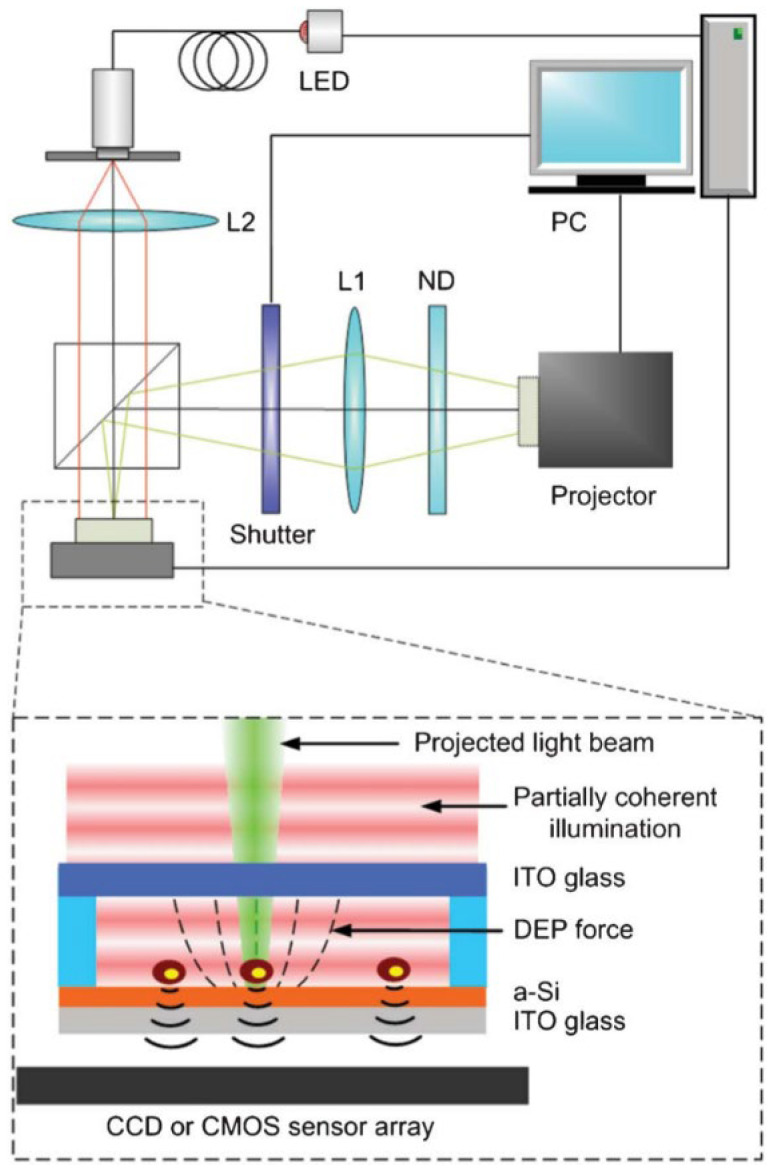
A schematic diagram of the experimental setup of an optoelectronic tweezer (OET) integrated with lens-free holographic microscopy. An OET device is directly placed on a CCD or CMOS sensor array. The sensor array, coupled with a partially coherent light source, creates an on-chip holographic microscope with a field of view that is equal to the sensor array’s active area. With the microscopic information of the particles, interactive OET manipulation of the target objects is achieved by projecting light beams next to the targets. A computer with a customized LabView program is used to control and synchronize different modules. Reproduced with permission [425]. Copyright 2013, RSC Publishing.

Optoelectronic tweezers have been widely used and studied as a tool with micromanipulation ability. When the semiconductor photoconductive material is illuminated by light, there is a difference in the conductivity between the illuminated region and the non-illuminated region. The illuminated region is used as the conductive electrode, and the non-illuminated region is the insulator. The non-uniform electric field applied in the illuminated region can achieve micromanipulation using dielectric electrophoresis force. Traditional photoelectric tweezers require a light modulator, such as an LCD or digital micro-mirror device (DMD) with a light source component, to project the modulation light pattern onto the photoconductive material in order to achieve the function of electrodes [421]. However, the large size of the LCD and DMD limits its operational accuracy, and the external light source limits its micro-integration. Fortunately, the micro-LED application can replace the light modulator and light source assembly, solving the difficulties. At present, optoelectronic tweezers with micro-LED have successfully completed the manipulation of polystyrene particles, hamster cells, lymphocytes, and so on [422,423]. For more research progress on photoelectric tweezers, please refer to the review article by Zhang et al. [424]. Figure 36 shows the relevant schematic for the optoelectronic tweezer.

##### Time-Resolved Fluorescence Measurement

Fluorescence analysis in the field of biomedicine can be used for imaging biomolecules, DNA sequencing, and so on. Effective biological information can be obtained by measuring the fluorescence signal generated by photoexcitation. The measurement of fluorescence signals consists mainly of steady-state measurement and time-resolved measurement. Compared with steady-state measurement, time-resolved measurement has the advantages of high sensitivity and selectivity and can obtain more information, such as molecular interactions. The time resolution measurement mainly relies on the narrow external band excitation light source to generate the fluorescence signal and extracts useful information by measuring the attenuation life of the fluorescence signal. Traditionally, lasers and mercury lamps, which are large and require filters, are used as excitation sources, which often increases the cost. In recent years, the most popular method has been the pulse diode coupled with a radiator as the excitation light source. This method can make up for the shortcomings of mercury lamps and lasers, but it is difficult to meet the integration standard. In order to further miniaturize and reduce the cost to meet the integration standard, a measurement system with micro-LED as the exciting light source has been developed. More than a decade ago, the shortest pulse excitation time had reached the picosecond order [426].

#### 4.4.5. White Light LEDs Illumination

Torches, candles, incandescent lamps, fluorescent lamps, LEDs—changes in the way humans illuminate themselves reflect the progress of the entirety of human civilization. Since the beginning of the new century, saving resources has been one of the main themes of development. For this reason, LEDs have been developed for lighting applications. At present, there are mainly three ways to use LEDs to generate white light, one of which directly uses three primary color (red, green, and blue) LEDs to synthesize white light. This way has the advantages of high luminous efficiency, controllable color temperature, and good color rendering, but it also has the disadvantages of unstable color temperature due to different light decay of three primary colors and high cost. The second is blue LEDs exciting phosphors (such as yellow phosphors or yellow phosphors with a small amount of red and green phosphors as the additions) to synthesize white light. It has the advantages of high efficiency, simple preparation, good temperature stability, and relatively good color rendering, but it also has the disadvantages of poor consistency and color temperature changing with the emission angle. The third is UV LEDs, exciting red, green, and blue phosphors to synthesize white light. It has the advantages of good color rendering and simple preparation, but it has the disadvantages of low luminous efficiency of UV LEDs and UV light leakage.

Most of the commercial white LEDs on the market use blue LEDs and exciting phosphors to obtain white light. Although they have a simple structure and good control, their excess blue light inevitably causes damage to the human eye, and the energy loss when using phosphors for light conversion is not negligible. Therefore, in the field of white LEDs, a great deal of research has been devoted to reducing the proportion of blue light, such as by using the LEDs themselves to produce blue and green light, the blue LEDs with phosphors to produce red light or the three-color light produced by the LEDs themselves. Then, LEDs with high efficiency became the theme, which is consistent with the theme of this review. Another idea is to develop efficient phosphors simply to improve blue light absorption efficiency and phosphor luminous efficiency. For relevant research progress, we can refer to the review articles of Xiang et al. and Cho et al. [427,428].

## 5. Will Nano-LED Be the Future after Micro-LED?

As the potential of micro-LED began to emerge in many fields, its research value was recognized by people. Although there are still some difficulties, this does not obscure the temptation brought by the advantages. It is worth considering whether such a strong incentive exists to continue shrinking devices to the nanometer scale to develop so-called nano-LEDs. Will nano-LED be the future after micro-LED? In this chapter, we will discuss this topic from three aspects: device structure, integration, and application.

### 5.1. Device Structure

Research on nano-LEDs is still in its infancy, but it is not unknown. Presently, relevant research has been carried out on some nanostructures, such as nanowires and QDs, and relevant experience has been accumulated. Core–shell nanowires, vertical nanowires, QD clusters, QD-nanowire binding structures, and other nanocrystalline structures are all feasible device structure schemes. It is predictable that the individual drives of these nanostructures are the directions of the nano-LEDs. Compared with traditional thin-film planar structures, these nanowires and QD structures have advantages in releasing strain, reducing QCSE, and increasing the effective active area. However, taking nanowires as an example, the performance of LEDs based on nanowires has not reached expectations. In self-growing epitaxial nanostructures, the effect of surface non-radiative recombination mechanisms increases as the size decreases and material quality improves, in addition to the inevitable surface damage caused by nanostructures that rely on additional semiconductor process steps, which is a challenge. To solve these problems, studies have been reported on ways to suppress the SRH of nanowires and material growth methods to increase the internal quantum (references [221,222] for details), but this is far from enough. In terms of the process fabrication of nanostructures, in order to avoid excessive surface damage caused by ICP etching, related wet etching processes and wet and dry etching methods were developed and proved effective [429,430,431]. And in order to improve the luminescence intensity, the passivation layer elimination etching process of core–shell nanowires that were grown on selected areas was explored [432]. Additionally, nano-printing transfer technology is also a solution for large-scale manufacturing. However, in addition to these, there are other specific mechanisms that we do not know about in the nanowire and QD structures that differ from the planar structure, limit the performance of nanostructures, and need more research to find them. For example, the effect of superlattice structure on the morphology and luminescence of core–shell nanowires has recently been investigated [433]. In order to understand the special physical mechanisms of nanostructures such as QDs and nanowire junctions, it is essential to improve the relevant characterization methods. For example, a method for characterizing the stress distribution of nanowires has been reported recently, and more such reports are needed [234].

### 5.2. Integration

The main challenges in the integration of nano-LED include the manufacturing and transfer of large-scale nano-LED arrays and the problem of individual driving. In terms of self-growing nanowire arrays, there has been a great improvement from low yield and uneven alignment at the beginning to large-scale, neat growth. Fabricating large-scale, neat nanowire arrays using top-down etching methods has been reported and is not difficult. With the rise of flexible electronics, the transfer technology of nanowires has attracted much attention, and relevant studies have been reported. For details, please refer to the review of Guan et al. [434]. The large-scale manufacturing and transfer technology of colloidal QDs has become more and more mature, and the growth of QDs in GaN material systems is no problem at present, but the large-scale transfer technology of group-III nitride QDs is still lacking. Perhaps it can refer to nanowire-related methods.

**Figure 37:**
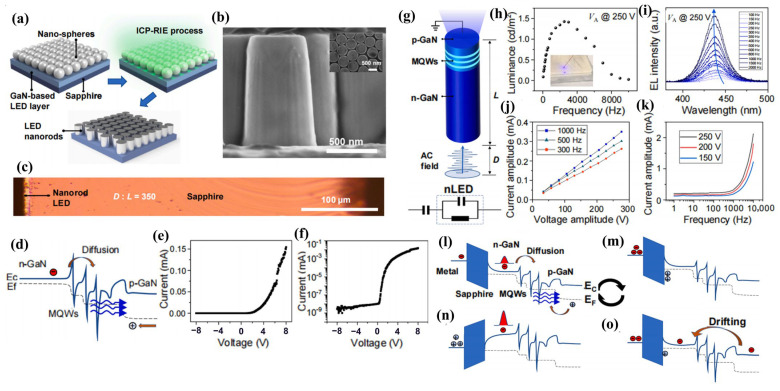
(**a**) Schematic of the fabrication of nanorod-LEDs. (**b**) Cross-section SEM image of nanorod LEDs. Inset: Top-view SEM image of the nanorod-LEDs. (**c**) Cross-section optical microscope image of nanorod-LEDs on a sapphire substrate. (**d**) Schematic band diagram of an LED in DC drive mode. (**e**,**f**) I–V curve of nanorod LED in DC drive mode. (**g**) Schematic of the single-dielectric AC-nanorod LED. Bottom panel: Equivalent circuit. (**h**) Peak luminescence-frequency relationship. Inset: Photograph of the single-dielectric ACnanorod LED. (**i**) EL spectra of the device at different driving frequencies. (**j**) Peak current–peak voltage relationship at different driving frequencies. (**k**) Peak current–frequency relationship at different voltages. Schematic band diagram of the single-dielectric ACnanorod LED. (**l**) Schematic of electron transfer when a forward bias is applied. (**m**) Steady-state band diagram of the device under forward bias. (**n**) Schematic of electron transfer when a reverse bias is applied. (**o**) Steady-state band diagram of the device under reverse bias. Reproduced with permission [439]. Copyright 2021, Elsevier Publishing.

Whether these nanostructures can be independently driven is a measure of whether they are nano-LEDs. We have introduced LEDs based on nanowires and QDs, but they cannot be called “nano-LEDs” because they do not single-drive the nanostructure alone. There are higher barriers to single-drive technology than mass manufacturing and transfer. Signal crosstalk, hybrid integration, and ohmic contact with the electrode are the problems faced by a nano-LED single drive [435]. To eliminate electrical and optical signal crosstalk, a ground electrode can be introduced between the electrodes, and a black matrix can be used [436]. In addition, appropriate electrode size and material are effective in reducing optical signal crosstalk [437]. However, the current technology level cannot achieve an error of a few nanometers in the alignment of the CMOS driver circuit for hybrid integration, for which the same substrate manufacturing process for the drive transistor and nano-LED is an ideal alternative. Wu et al. proposed to use in-plane solid–liquid–solid growth technology to manufacture thin field transistors and nano-LEDs on the same substrate so as to realize the single-driver integration scheme [381]. In addition to the improvements of the traditional method, the non-contact mode provides a new way of thinking about ohmic contact [438,439]. Figure 37 shows a schematic of the non-contact method and the associated experimental results. Recently, a small-scale array fabrication scheme for nano-LED that can be directly addressed and single-driven has been reported by Bezshlyakh et al., and more programs and practices are urgently needed to break through the barriers [440]. Surprisingly and remarkably, Shin et al. stacked a high-density micro-LED array of only 9 μm in height with 5100 pixels per inch and only 4 μm in pixels using a two-dimensional material layer transfer technique and achieved full-color display operation of the array through vertical integration. It can be said that the success of this study has accumulated experience and increased confidence for LEDs to move to the nanoscale [13].

### 5.3. Applications

For the display field, as the pixels are reduced to hundreds of nanometers or even lower, the minimum limit that human eyes can distinguish has been broken, and the visual experience is no different from that of micro-LED at the micron level [441]. However, due to their reduced size and proven high extraction efficiency, nano-LEDs have significantly impacted the development of smaller, low-power displays. Moreover, high extraction efficiency and the elimination of the TM mode make it very attractive to achieve high efficiency and luminescence in UV [442]. In the field of microscopy, because its nano-LED-based photodetectors can achieve direct imaging of objects without dependence on complex optical systems and break the limit of optical resolution, nano-LED has a promising future in the development of super-resolution microscopes [441]. For high-speed light communication, it has been reported that the response time of LEDs based on nanowire arrays has been reduced to the level of several hundred picoseconds [443]. Further development on this basis will have epochal significance for high-speed visible-light communication, especially high-speed ultraviolet-light communication. And, of course, smaller sizes and a better cell fit mean more accurate results in photogenetic studies.

We have discussed the prospect of nano-LED from different angles. The potential of nano-LEDs in some areas is surprising, and if they work, it will be a fundamental leap forward, especially in the application of detection, such as a super-resolution microscope. As we all know, the premise of human scientific and technological progress is the progress of detection means, so that people can understand unknown things. We hope that nano-LED can undertake this task in the future and promote the development of other disciplines. But at present, nano-LED still has some shortcomings in its basic theory that need further exploration. In some aspects, the challenge is relatively large; some programs have been proposed, but the practice is less common. In general, the lack of relevant research on nano-LED is one of the main reasons restricting its development. Will nano-LED be the future after micro-LED? The answer to this question should be gradually clarified after the theoretical mechanisms of nanostructured group-III nitride materials are perfect.

## 6. Conclusions and Prospect

After more than 30 years of development, group-III nitride LEDs continue to make breakthroughs and benefit humanity. The road to advancement is only sometimes smooth, and continuous progress is inseparable from the efforts and dedication of researchers, which is worth learning and commemorating. So, in group-III nitride, we reviewed the development from large-size LEDs to micro-LEDs and then prospected nano-LEDs in group-III nitride that may emerge in the future, which covers a longer time frame compared to other similar reviews.

The scenery along the way is beautiful, but crossing the chasm is even more exciting. Therefore, this review focuses on the challenges and solutions encountered in the research process. In a nutshell, these challenges range from the initial difficulty of efficient blue luminescence to the current problem of efficient UV and red luminescence. The solutions are reflected in the device structure and technology. Device structures include novel designs of superlattice and multiple quantum well structures applied to planar-layer structures, low-dimensional QDs, nanowires, and other nanostructures, resulting in positive feedback physical mechanisms. Technologies include improved material growth methods such as epitaxial layer growth on patterned substrates, beveled substrates, and multi-step growth on AlN templates. In addition, p-type doping techniques, such as semiconductor superlattice doping, δ-doping, polarization-induced doping, and potential doping modes, have also improved p-type characteristics. In addition, the application of ELOG technology and color conversion technology further improves the material growth quality and extension of the luminescence wavelength to longer wavelengths. However, these methods still need further research to meet people’s needs for higher-performance LEDs. On the other hand, it has been demonstrated that nitride LED downscaling to the micron order has advantages in mitigating efficiency droop, dissipating heat, improving LEE, etc. Similarly, when the micron is reduced to the nanometer size, the potential of nanostructures represented by nanowires and QDs, etc., in p-type doping, QCSE inhibition, defect suppression, and red-light emission has been discovered.

As we face the future, developing group-III nitride nano-LEDs is a critical way to improve the luminous efficiency of ultraviolet and red. However, new challenges in developing nano-LEDs also arise, when the LED size is reduced to the nanoscale, the high surface area share increases the non-radiative recombination share of the material surface, thereby reducing the luminous efficiency. The reduction in size brings significant quantum effects that enhance the coupling probability of neighboring luminous units and ultimately introduce the problem of signal crosstalk; in addition, the increase in the difficulty of electrical injection also imparts luminous efficiency and integration problems in the context of LEDs. Subsequently, relevant methods were proposed by researchers to solve these problems. For example, the solution treats the material’s surface to reduce its surface states; the black matrix method reduces signal crosstalk; the non-contact method solves the driving problem, etc. However, more is needed. The physical mechanisms associated with nanostructures should be studied in depth to understand the source of the problems, from nature to change, and solve the problems encountered regarding materials and structure. In addition, it is necessary to develop stacked processes to solve problems such as signal crosstalk, driving, and integration.

**Figure 38:**
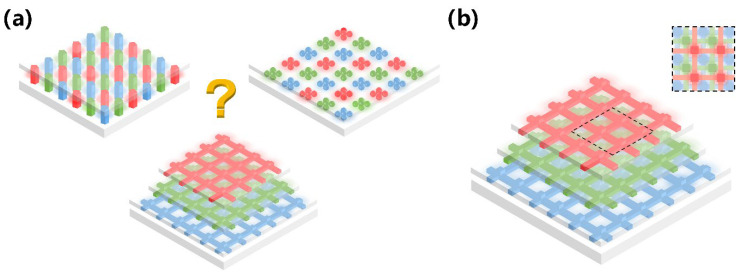
(**a**) Three possible nano-LED structures may exist in the future, including nanowire structures, QD structures, and nano-grid stacking structures. (**b**) A nano-grid stacking structure and its bird’s-eye view of possible local pixel alignment.

Here, a new full-color nano-grid stacking scheme with nodes as the active region is proposed (as shown in Figure 38). For the same pixel density, stacked nano-grid solutions require fewer driver ports for individual driving compared with independent addressing to drive full-color nanowires or QDs in the same plane. And the wider port spacing dramatically reduces the difficulty of adopting a more mature contact drive method. A large-scale transfer is simpler for interconnected grid structures, and the ample grid space reduces signal crosstalk and alignment difficulties. As for material growth, nanoscale device fabrication can be achieved with a grid diameter of less than about 3 μm. Although group-III nitride nano-grid materials’ growth is rarely reported, there are relevant technical accumulations, such as vertical growth in selected areas. In terms of device material layer structure design, a monolithic integrated design with a GaN-based transistor structure can maximize its advantages. Practice begets actual knowledge; challenges will eventually be overcome after continuous improvement. Expect this kind of green, energy-saving display technology to make the future more colorful.

## Figures and Tables

**Table 1:** A summary of group-III nitride materials grown on conventional substrates by different growth methods in recent years.

Material	Substrate/Buffer Layer	Growth Method	(0002) Full Width at Half Maximum (FWHM) [arcsec]	$\left(10\bar{1}2\right)$ FWHM [arcsec]	Others	Ref.
GaN	Sapphire/GaN	MOCVD and HVPE	507	463	---	[34]
GaN	Sapphire/GaN	MOCVD and HVPE	256	286	Treatment by laser decomposition technique	[34]
GaN	Sapphire/---	HVPE	300	600	Beveled substrate	[35]
GaN	Si/AlN and AlGaN	MOCVD and HVPE	62	---	Threading dislocation density: 10^6^/cm^2^.	[36]
GaN	6H-SiC/GaN	HVPE	261	272	---	[37]
GaN	Sapphire/AlN	MOCVD	410	---	N-polar GaN	[38]
GaN	Sapphire/AlN and AlGaN	MOCVD	35	220	Patterned sapphire substrate	[39]
GaN	Si/AlN and AlGaN	MOCVD	298	324	---	[40]
GaN	4H-SiC/AlN and GaN	MOCVD	144	306	AlN interlayer	[41]
GaN	Sapphire/Graphene	MBE	720	792	Non-polar substrate/N-polar GaN	[42]
GaN	Si/GaN	MBE	450	---	GaN nanowire buffer layer	[43]
GaN	4H-SiC/---	Plasma-assisted MBE	159	848	---	[44]
AlN	Sapphire/---	HVPE	70	395	---	[45]
AlN	Sapphire/---	HVPE	102	219	Patterned sapphire substrate	[45]
AlN	Si/---	HVPE	1292	---	Semi-polar AlN	[46]
AlN	6H-SiC/---	HVPE	228	766	---	[47]
AlN	Sapphire/---	MOCVD	9	334	Face-to-face annealed sputtered AlN	[48]
AlN	Sapphire/---	MOCVD	13	123	Face-to-face annealed sputtered AlN/Beveled sapphire substrate	[49]
AlN	Si/---	MOCVD	409	677	---	[50]
AlN	6H-SiC/---	MOCVD	17	246	Face-to-face annealed sputtered AlN	[51]
AlN	4H-SiC/---	MOCVD	355	840	---	[52]
AlN	Sapphire/---	MBE	103.6	353.1	Face-to-face annealed sputtered AlN	[53]
AlN	Sapphire/AlN	MBE	14	380	N-polar AlN	[54]
AlN	Si/GaN	MBE	972	---	GaN nanowire buffer layer/N-polar AlN	[35]
Al_0.17_Ga_0.83_N	Sapphire and GaN/---	HVPE	---	600	Beveled substrate	[49]
Al_0.67_Ga_0.33_N	Sapphire and AlN/AlGaN	HVPE	---	300	Beveled substrate	[49]
Al_0.68_Ga_0.32_N	Sapphire and AlN/AlGaN	HVPE	---	300	Patterned sapphire substrate	[49]
Al_0.21_Ga_0.79_N	Sapphire and AlN/GaN	MOCVD	167.3	240.4	Patterned sapphire substrate	[55]
Al_0.74_Ga_0.26_N	Sapphire and AlN/---	MOCVD	50	210–230	Face-to-face annealed sputtered AlN/Beveled sapphire substrate	[56]
Al_0.53_Ga_0.43_N	Si and AlN/---	MOCVD	149	---	Pulsed co-doping	[57]
Al_0.50_Ga_0.50_N	6H-SiC and AlN/AlGaN	MOCVD	280	1028	---	[58]
Al_0.50_Ga_0.50_N	6H-SiC and AlN/AlGaN	MOCVD	235–240	766	SiN_X_ interlayer	[58]
Al_0.23_Ga_0.77_N	Sapphire and AlN/Graded Al_X_Ga_1−X_N	MBE	1569	---	Non-polar a-plane Al_0.23_Ga_0.77_N	[59]
Al_0.6_Ga_0.4_N	Sapphire and AlN/---	MBE	432	---	---	[60]

**Table 2:** Summary of related research on different types of PPS.

Shape	Size [µm]	Depth/Height [µm]	Slanted Angle [°]	Density [cm^−2^]	(0002) FWHM [arcsec]	$\left(10\bar{1}2\right)$ FWHM [arcsec]	Output Power [mW]	Quantum Efficiency [%]	Ref.
---	---	---	---	---	---	---	8.6	11.6 (External)	[64]
Round hole	3	1.5	90	---	---	---	10.4	14.1 (External)	[64]
Pyramid	---	1.2	57.4	4.31 × 10^7^	269.3	410.3	15.2	56.5 (Internal)	[65]
Pyramid	---	1.2	45.3	1.11 × 10^7^	264.1	356.6	17.2	60.7 (Internal)	[65]
Pyramid	---	1.2	38.3	0.87 × 10^7^	251.1	312.6	18.5	61.6 (Internal)	[65]
Pyramid	---	1.2	36.1	0.52 × 10^7^	243.4	301.2	20.8	66.1 (Internal)	[65]
---	---	---	---	---	---	338	5.4	---	[66]
Cone	3	1.5	45	---	---	225	7.3	---	[66]
---	---	---	---	---	410	560	7.93	12.59 (External)	[67]
---	3	1.5	---	1 × 10^8^	300	305	9.27	14.97 (External)	[67]
---	2	1.5	---	1 × 10^8^	300	305	9.53	15.28 (External)	[67]
---	0.45	0.15	---	3.5 × 10^8^	330	380	10.27	16.39 (External)	[67]

**Table 3:** Summary of data from AlN template-controlled trials of two- and three-step growth methods.

AlN Sample	Substrate	AlN Template Layer Structure Thickness [nm]				(0002) FWHM [arcsec]	$\left(10\bar{1}2\right)$ FWHM [arcsec]	Root Means Square (RMS) [nm]	Ref.
		Low-Temperature Nucleation Layer	High-Temperature Growth Layer	Intermediate Epitaxial Layer	High-Temperature Growth Layer				
Two-step growth method	Conventional sapphire substrate	35 (950 °C)	1010 (1280 °C)	---	---	109	751	0.463	[72]
Three-step growth method	Conventional sapphire substrate	35 (950 °C)	20 (1280 °C)	60 (1050 °C)	750 (1280 °C)	100	738	0.121	[72]
Two-step growth method	Patterned sapphire substrate	3 (870 °C)	700 (1110 °C)	---	---	795	2160	0.3	[73]
Two-step growth method	Patterned sapphire substrate	3 (870 °C)	700 (1250 °C)			65	1150	1.2	[73]
Three-step growth method	Patterned sapphire substrate	3	---	150 (1250 °C)	3200 (1110 °C)	50	250	0.5	[73]
Three-step growth method	Conventional sapphire substrate	3	---	150 (1250 °C)	3200 (1110 °C)	400	800	0.2	[73]

**Table 4:** Relevant p-type doping data are in this chapter.

Doped Material	Doping Method	Hole Concentration [10^17^/cm^3^]	Hole Mobility [cm^2^/V·s]	Others	Year	Ref.
GaN	Thermal annealing	3	10	---	1992	[279]
Al_0.4_Ga_0.6_N	Thermal annealing	3.26	--	---	2015	[294]
Al_0.4_Ga_0.6_N	Delta doping	15.9	---	---	2015	[294]
Al_0.4_Ga_0.6_N	Delta doping	47.5	---	Indium-surfactant-assisted	2015	[294]
Al_0.42_Ga_0.58_N	Delta doping	49.7	1.35	Indium-surfactant-assisted	2020	[295]
Al_0.42_Ga_0.58_N	Delta doping	4.61	2.52	Indium-surfactant-assisted/Pulsed TMAl flow	2020	[295]
Al_0.42_Ga_0.58_N	Delta doping	83	1.47	Indium-surfactant-assisted/Pulsed TMGa flow	2020	[295]
Al_0.46_Ga_0.54_N/Al_0.63_Ga_0.37_N	Superlattice doping	81	---	Desorption-tailoring strategy	2022	[287]
B_0.14_Al_0.86_N/Al_0.5_Ga_0.5_N	Strain-compensated B_0.14_Al_0.86_N/Al_0.5_Ga_0.5_N polarization-induced superlattice doping	80.7	---	Simulated result	2022	[302]
Al_0.6_Ga_0.4_N	Delta doping with the Al_0.6_Ga_0.4_N/GaN superlattice modulation doping	5.1	4.7	Indium-surfactant-assisted	2019	[296]
Al_0.75_Ga_0.25_N/AlN	Superlattice doping	34	--	--	2018	[286]
AlN/GaN	Polarization-induced doping	5 × 10^−4^	25	Dopant free	2019	[300]
Al_x_Ga_1−x_N (x:0 to 0.3)	Polarization-induced doping	19	---	---	2022	[301]
Al_0.3_Ga_0.7_N/Al_0.15_Ga_0.85_N	Nanowire doping	130	---	---	2018	[304]

**Table 6:** Summary of related research progress on UVB LED.

Peak Wavelength [nm]	Active Region	EQE [%]	WPE [%]	Single Pixel Size [μm]	Ligh Output Power [mW]	Power Density [W/cm^2^]	Ref.
228	Al_0.82_Ga_0.18_N/Al_0.94_Ga_0.06_N MQW	---	---	400	1.4 (at 150 mA)	Around 0.88	[411]
232	---	0.57	---	---	4.2 (at 200 mA)	---	[412]
254	Al_0.64_Ga_0.36_N/Al_0.78_Ga_0.22_N MQW	1.2	---	350	3.2 (at 75 mA)	Around 2.61	[413]
258	Al_0.6_Ga_0.4_N/Al_0.74_Ga_0.26_N MQW	2.2	---	350	7 (at 75 mA)	Around 5.71	[414]
265	Al_0.6_Ga_0.4_N/Al_x_Ga_1−x_N (x > 0.6) MQW	11	7.6	---	---	---	[354]
275	Al_0.4_Ga_0.6_N/Al_0.55_Ga_0.45_N MQW	20.3	---	---	18.3 (at 20 mA)	---	[353]
275	Al_0.4_Ga_0.6_N/Al_0.55_Ga_0.45_N MQW	---	---	---	44.2 (at 50 mA)	---	[353]
275	Al_0.45_Ga_0.55_N/Al_0.6_Ga_0.4_N MQW	1.2	---	20	Around 0.15 (at around 6 mA)	86	[414]
278	Al_0.39_Ga_0.61_N/Al_0.6_Ga_0.4_N MQW	---	---	---	8 (at around 100 mA)	8.2	[415]
